# Supplementary material for: Towards universal health coverage for people with stroke in South Africa: a scoping review
Source: BMJ Open. 2021 Nov 24;11(11):e049988. doi: 10.1136/bmjopen-2021-049988 (PMC8627414; doi:10.1136/bmjopen-2021-049988)
Supplement: Supplementary data [file bmjopen-2021-049988supp001.pdf]

BMJ Publishing Group Limited (BMJ) disclaims all liability and responsibility arising from any reliance placed on this supplemental material which has been supplied by the author(s)

Supplemantary File - 1

Achieving Universal Health Coverage for people with stroke in South Africa:

OVID Search

Database: Embase <1980 to 2020 Week 24>, Global Health <1910 to 2020 Week 23>, Journals@Ovid Full Text <June 17, 2020>, APA PsycExtra <1908 to June 08, 2020>, APA PsycInfo <1806 to June Week 2 2020>, LSHTM Journals@Ovid, Econlit <1886 to June 11, 2020>, Ovid MEDLINE(R) and Epub Ahead of Print, In-Process & Other Non-Indexed Citations, Daily and Versions(R) <1946 to June 16, 2020>, Social Policy and Practice <202004>
Search Strategy:
----

1 Stroke.mp. [mp=ti, ab, hw, tn, ot, dm, mf, dv, kw, fx, dq, bt, id, cc, tx, sh, ct, tc, tm, mh, nm, kf, ox, px, rx, an, ui, sy, pt] (1263546)
2 cerebro vascular accident.mp. [mp=ti, ab, hw, tn, ot, dm, mf, dv, kw, fx, dq, bt, id, cc, tx, sh, ct, tc, tm, mh, nm, kf, ox, px, rx, an, ui, sy, pt] (560)
3 ischaemia.mp. [mp=ti, ab, hw, tn, ot, dm, mf, dv, kw, fx, dq, bt, id, cc, tx, sh, ct, tc, tm, mh, nm, kf, ox, px, rx, an, ui, sy, pt] (140097)
4 hemorrhage.mp. [mp=ti, ab, hw, tn, ot, dm, mf, dv, kw, fx, dq, bt, id, cc, tx, sh, ct, tc, tm, mh, nm, kf, ox, px, rx, an, ui, sy, pt] (923222)
5 Universal health coverage.mp. [mp=ti, ab, hw, tn, ot, dm, mf, dv, kw, fx, dq, bt, id, cc, tx, sh, ct, tc, tm, mh, nm, kf, ox, px, rx, an, ui, sy, pt] (9467)
6 universal access.mp. [mp=ti, ab, hw, tn, ot, dm, mf, dv, kw, fx, dq, bt, id, cc, tx, sh, ct, tc, tm, mh, nm, kf, ox, px, rx, an, ui, sy, pt] (12850)
7 Universal health care.mp. [mp=ti, ab, hw, tn, ot, dm, mf, dv, kw, fx, dq, bt, id, cc, tx, sh, ct, tc, tm, mh, nm, kf, ox, px, rx, an, ui, sy, pt] (7370)
8 universal health access.mp. [mp=ti, ab, hw, tn, ot, dm, mf, dv, kw, fx, dq, bt, id, cc, tx, sh, ct, tc, tm, mh, nm, kf, ox, px, rx, an, ui, sy, pt] (64)
9 stroke disability.mp. [mp=ti, ab, hw, tn, ot, dm, mf, dv, kw, fx, dq, bt, id, cc, tx, sh, ct, tc, tm, mh, nm, kf, ox, px, rx, an, ui, sy, pt] (1581)
10 stroke rehabilitation.mp. [mp=ti, ab, hw, tn, ot, dm, mf, dv, kw, fx, dq, bt, id, cc, tx, sh, ct, tc, tm, mh, nm, kf, ox, px, rx, an, ui, sy, pt] (30385)
11 stroke care.mp. [mp=ti, ab, hw, tn, ot, dm, mf, dv, kw, fx, dq, bt, id, cc, tx, sh, ct, tc, tm, mh, nm, kf, ox, px, rx, an, ui, sy, pt] (18395)
12 treatment access.mp. [mp=ti, ab, hw, tn, ot, dm, mf, dv, kw, fx, dq, bt, id, cc, tx, sh, ct, tc, tm, mh, nm, kf, ox, px, rx, an, ui, sy, pt] (6626)
13 health systems.mp. [mp=ti, ab, hw, tn, ot, dm, mf, dv, kw, fx, dq, bt, id, cc, tx, sh, ct, tc, tm, mh, nm, kf, ox, px, rx, an, ui, sy, pt] (111350)
14 South Africa.mp. [mp=ti, ab, hw, tn, ot, dm, mf, dv, kw, fx, dq, bt, id, cc, tx, sh, ct, tc, tm, mh, nm, kf, ox, px, rx, an, ui, sy, pt] (235927)
15 Eastern Cape.mp. [mp=ti, ab, hw, tn, ot, dm, mf, dv, kw, fx, dq, bt, id, cc, tx, sh, ct, tc, tm, mh, nm, kf, ox, px, rx, an, ui, sy, pt] (4355)
16 Free State.mp. [mp=ti, ab, hw, tn, ot, dm, mf, dv, kw, fx, dq, bt, id, cc, tx, sh, ct, tc, tm, mh, nm, kf, ox, px, rx, an, ui, sy, pt] (14110)
17 Gauteng.mp. [mp=ti, ab, hw, tn, ot, dm, mf, dv, kw, fx, dq, bt, id, cc, tx, sh, ct, tc, tm, mh, nm, kf, ox, px, rx, an, ui, sy, pt] (4356)
18 KwaZulu-Natal.mp. [mp=ti, ab, hw, tn, ot, dm, mf, dv, kw, fx, dq, bt, id, cc, tx, sh, ct, tc, tm, mh, nm, kf, ox, px, rx, an, ui, sy, pt] (11901)

19 Limpopo.mp. [mp=ti, ab, hw, tn, ot, dm, mf, dv, kw, fx, dq, bt, id, cc, tx, sh, ct, tc, tm, mh, nm, kf, ox, px, rx, an, ui, sy, pt] (3368)
20 Mpumalanga.mp. [mp=ti, ab, hw, tn, ot, dm, mf, dv, kw, fx, dq, bt, id, cc, tx, sh, ct, tc, tm, mh, nm, kf, ox, px, rx, an, ui, sy, pt] (1992)
21 Northern Cape.mp. [mp=ti, ab, hw, tn, ot, dm, mf, dv, kw, fx, dq, bt, id, cc, tx, sh, ct, tc, tm, mh, nm, kf, ox, px, rx, an, ui, sy, pt] (997)
22 North West.mp. [mp=ti, ab, hw, tn, ot, dm, mf, dv, kw, fx, dq, bt, id, cc, tx, sh, ct, tc, tm, mh, nm, kf, ox, px, rx, an, ui, sy, pt] (39969)
23 Western Cape.mp. [mp=ti, ab, hw, tn, ot, dm, mf, dv, kw, fx, dq, bt, id, cc, tx, sh, ct, tc, tm, mh, nm, kf, ox, px, rx, an, ui, sy, pt] (8316)
24 1 or 2 or 3 or 4 (2162628)
25 5 or 6 or 7 or 8 (28371)
26 9 or 10 or 11 or 12 (54898)
27 15 or 16 or 17 or 18 or 19 or 20 or 21 or 22 or 23 (83478)
28 14 or 27 (286527)
29 13 or 25 (135636)
30 24 and 29 (7926)
31 24 and 26 and 29 (655)
32 24 and 28 and 29 (433)

****

UHC for Stroke care in SA - Scopus Search results

(Stroke OR Universal health coverage) AND (Health system) AND (LIMIT-TO (AFFILCOUNTRY,"South Africa")) AND (LIMIT-TO (PUBYEAR,2020) OR LIMIT-TO (PUBYEAR,2019) OR LIMIT-TO (PUBYEAR,2018) OR LIMIT-TO (PUBYEAR,2017) OR LIMIT-TO (PUBYEAR,2016) OR LIMIT-TO (PUBYEAR,2015) OR LIMIT-TO (PUBYEAR,2014) OR LIMIT-TO (PUBYEAR,2013) OR LIMIT-TO (PUBYEAR,2012) OR LIMIT-TO (PUBYEAR,2011) OR LIMIT-TO (PUBYEAR,2010) OR LIMIT-TO (PUBYEAR,2009) OR LIMIT-TO (PUBYEAR,2008) OR LIMIT-TO (PUBYEAR,2007) OR LIMIT-TO (PUBYEAR,2006) OR LIMIT-TO (PUBYEAR,2004) OR LIMIT-TO (PUBYEAR,2003) OR LIMIT-TO (PUBYEAR,2002) OR LIMIT-TO (PUBYEAR,2000)) AND (LIMIT-TO (openaccess,1)) AND (LIMIT-TO (SUBJAREA,"MEDI") OR LIMIT-TO (SUBJAREA,"SOCI") OR LIMIT-TO (SUBJAREA,"MULT") OR LIMIT-TO (SUBJAREA,"NURS") OR LIMIT-TO (SUBJAREA,"HEAL") OR LIMIT-TO (SUBJAREA,"ENVI") OR LIMIT-TO (SUBJAREA,"PSYC") OR LIMIT-TO (SUBJAREA,"ECON") OR LIMIT-TO (SUBJAREA,"ARTS") OR LIMIT-TO (SUBJAREA,"NEUR")) AND (LIMIT-TO (PUBSTAGE,"final") OR LIMIT-TO (PUBSTAGE,"aip"))

		Narrow by SubjectThesaurus: - qualitative research<br>Narrow by SubjectThesaurus: - primary care<br>Narrow by SubjectThesaurus: - evaluation research<br>Narrow by SubjectThesaurus: - health behavior<br>Narrow by SubjectThesaurus: - community health services<br>Narrow by SubjectThesaurus: - health outcome assessment<br>Narrow by SubjectThesaurus: - government policy<br>Narrow by SubjectThesaurus: - world health<br>Narrow by SubjectThesaurus: - quality of life<br>Narrow by SubjectThesaurus: - health services accessibility<br>Narrow by SubjectThesaurus: - health promotion<br>Narrow by SubjectThesaurus: - public health<br>Search modes - Boolean/Phrase		
S5	(stroke or cerebrovascular accident or cva or cerebral vascular event or cve or transient ischaemic attack or tia) OR (stroke rehabilitation or stroke recovery) OR stroke	Limiters - Published Date: 20050101-20200631<br>Expanders - Apply related words; Apply equivalent subjects<br>Narrow by Language: - english	Interface - EBSCOhost Research Databases<br>Search Screen - Advanced Search<br>Database - Academic Search Complete	16,739

prevention OR stroke patients AND universal health care OR universal health coverage OR universal health coverage in south africa AND health system OR health systems strengthening OR (health system or health services) OR health systems management AND south africa	Narrow by SubjectThesaurus: - public health surveillance Narrow by SubjectThesaurus: - primary health care Narrow by SubjectThesaurus: - national health services Narrow by SubjectThesaurus: - health insurance Narrow by SubjectThesaurus: - health disparities Narrow by SubjectThesaurus: - health care reform Narrow by SubjectThesaurus: - health programs Narrow by SubjectThesaurus: - chronic diseases Narrow by SubjectThesaurus: - economics Narrow by SubjectThesaurus: - systematic reviews (medical research) Narrow by SubjectThesaurus: - qualitative research Narrow by SubjectThesaurus: - primary care Narrow by SubjectThesaurus: - evaluation research Narrow by SubjectThesaurus: - health behavior Narrow by SubjectThesaurus: - community health services Narrow by SubjectThesaurus: -

		health outcome assessment<br>Narrow by SubjectThesaurus: - government policy<br>Narrow by SubjectThesaurus: - world health<br>Narrow by SubjectThesaurus: - quality of life<br>Narrow by SubjectThesaurus: - health services accessibility<br>Narrow by SubjectThesaurus: - health promotion<br>Narrow by SubjectThesaurus: - public health<br>Search modes - Boolean/Phrase		
S4	(stroke or cerebrovascular accident or cva or cerebral vascular event or cve or transient ischaemic attack or tia) OR (stroke rehabilitation or stroke recovery) OR stroke prevention OR stroke patients AND universal health care OR universal health coverage OR universal health coverage in south africa AND health system OR health systems strengthening OR (health system or health services) OR health systems management AND south africa	Limiters - Published Date: 20050101-20200631<br>Expanders - Apply related words; Apply equivalent subjects<br>Narrow by SubjectThesaurus: - public health surveillance<br>Narrow by SubjectThesaurus: - primary health care<br>Narrow by SubjectThesaurus: - national health services<br>Narrow by SubjectThesaurus: - health insurance<br>Narrow by SubjectThesaurus: - health disparities<br>Narrow by SubjectThesaurus: - health care reform<br>Narrow by SubjectThesaurus: -	Interface - EBSCOhost Research Databases<br>Search Screen - Advanced Search<br>Database - Academic Search Complete	17,251

health programs
Narrow by SubjectThesaurus: - chronic diseases
Narrow by SubjectThesaurus: - economics
Narrow by SubjectThesaurus: - systematic reviews (medical research)
Narrow by SubjectThesaurus: - qualitative research
Narrow by SubjectThesaurus: - primary care
Narrow by SubjectThesaurus: - evaluation research
Narrow by SubjectThesaurus: - health behavior
Narrow by SubjectThesaurus: - community health services
Narrow by SubjectThesaurus: - health outcome assessment
Narrow by SubjectThesaurus: - government policy
Narrow by SubjectThesaurus: - world health
Narrow by SubjectThesaurus: - quality of life
Narrow by SubjectThesaurus: - health services accessibility
Narrow by SubjectThesaurus: - health promotion
Narrow by SubjectThesaurus: -

		public health<br>Search modes - Boolean/Phrase		
S3	(stroke or cerebrovascular accident or cva or cerebral vascular event or cve or transient ischaemic attack or tia) OR (stroke rehabilitation or stroke recovery) OR stroke prevention OR stroke patients AND universal health care OR universal health coverage OR universal health coverage in south africa AND health system OR health systems strengthening OR (health system or health services) OR health systems management AND south africa	Limiters - Published Date: 20050101-20200631<br>Expanders - Apply related words; Apply equivalent subjects<br>Narrow by SubjectThesaurus: - public health<br>Search modes - Boolean/Phrase	Interface - EBSCOhost Research Databases<br>Search Screen - Advanced Search<br>Database - Academic Search Complete	84,912
S2	(stroke or cerebrovascular accident or cva or cerebral vascular event or cve or transient ischaemic attack or tia) OR (stroke rehabilitation or stroke recovery) OR stroke prevention OR stroke patients AND universal health care OR universal health coverage OR universal health coverage in south africa AND health system OR health systems strengthening OR (health system or health services) OR health systems management AND south africa	Limiters - Published Date: 20050101-20200631<br>Expanders - Apply related words; Apply equivalent subjects<br>Search modes - Boolean/Phrase	Interface - EBSCOhost Research Databases<br>Search Screen - Advanced Search<br>Database - Academic Search Complete	878,271
S1	(stroke or cerebrovascular accident or cva or cerebral	Limiters - Published Date: 20050101-20200631	Interface - EBSCOhost Research Databases<br>Search Screen - Advanced	878,271

vascular event or cve or transient ischaemic attack or tia) OR (stroke rehabilitation or stroke recovery) OR stroke prevention OR stroke patients AND universal health care OR universal health coverage OR universal health coverage in south africa AND health system OR health systems strengthening OR (health system or health services) OR health systems management OR health system financing	Expanders - Apply related words; Apply equivalent subjects Search modes - Boolean/Phrase	Search Database - Academic Search Complete
