# Supplementary material for: Towards universal health coverage for people with stroke in South Africa: a scoping review
Source: BMJ Open. 2021 Nov 24;11(11):e049988. doi: 10.1136/bmjopen-2021-049988 (PMC8627414; doi:10.1136/bmjopen-2021-049988)
Supplement: Supplementary data [file bmjopen-2021-049988supp002.pdf]

Supplementary file S2

FRAMEWORK COMPONENT	DESCRIPTION (data items)
**Governance/Regulation**	**Description:** a wide range of steering and rulemaking-related functions carried out by governments/decisions makers as they seek to achieve national and/or provincial health policy objectives that are conducive to UHC<br>**Data Items:** Healthcare policies at national or provincial levels; resource allocation policies; accountability monitoring; coordination and regulations; clinical treatment guidelines.
**Resources**	**Description:** All resources specific to the health care facility – including the physical structure and resources enabling or hindering delivery of health services.<br>**Data Items:**<br>**Infrastructure:** Accessibility of the health care facilities; maintenance of infrastructure; availability of equipment/testing facilities (e.g. CT Scans)<br>**Human resources:** Availability; health workforce distribution – health professions/experience or specialisation/gender; role definitions; undergraduate & continuous training; workload; patient vs therapist ratio.<br>**Financial resources:** Finance allocation and affordability; funding sources; healthcare packages; salaries/fair wages.; sustainability.
**Service Delivery**	**Description:** Delivery of different health services as well as user experience.<br>**Data Items:** Level of care; comprehensiveness; quality and/or perceptions of care; multi-professional health teams; continuity of care, timeliness of care; health services and service providers (private/public; for-profit or not–for-profit, formal or informal, professional or non-professional, allopathic or traditional, remunerated or voluntary).
**Context**	**Description:** All contextual factors influencing the patient/community access of the health care system.<br>**Data Items:** Social determinants of health: socio-economic, education, health literacy, technological, cultural, political and environmental environments.
**Re-orientation of care**	**Description:** New and innovate health care solutions to improve coordination of health services and continuous health care; and intersectoral coordination.<br>**Data Items:** New technologies and strategies (eHealth; shared electronic medical records; telemedicine; m-health)
**Community engagement**	**Description:** Engaging and empowering individuals, families, communities and informal caregivers to facilitate common decision-making and self-efficacy. Reaching underserved and marginalised communities.

Application of the key characteristics of the analytical framework

FRAMEWORK COMPONENT	DESCRIPTION (data items)
**Governance/Regulation**	**Description:** a wide range of steering and rulemaking-related functions carried out by governments/decisions makers as they seek to achieve national and/or provincial health policy objectives that are conducive to UHC<br>**Data Items:** Healthcare policies at national or provincial levels; resource allocation policies; accountability monitoring; coordination and regulations; clinical treatment guidelines.
**Resources**	**Description:** All resources specific to the health care facility – including the physical structure and resources enabling or hindering delivery of health services.<br>**Data Items:**<br>**Infrastructure:** Accessibility of the health care facilities; maintenance of infrastructure; availability of equipment/testing facilities (e.g. CT Scans)<br>**Human resources:** Availability; health workforce distribution – health professions/experience or specialisation/gender; role definitions; undergraduate & continuous training; workload; patient vs therapist ratio.<br>**Financial resources:** Finance allocation and affordability; funding sources; healthcare packages; salaries/fair wages.; sustainability.
**Service Delivery**	**Description:** Delivery of different health services as well as user experience.<br>**Data Items:** Level of care; comprehensiveness; quality and/or perceptions of care; multiprofessional health teams; referral systems; service delivery models; health services and service providers (private/public; for-profit or not–for-profit, formal or informal, professional or non-professional, allopathic or traditional, remunerated or voluntary).
**Context**	**Description:** All contextual factors influencing the patient/community access of the health care system.<br>**Data Items:** Social determinants of health: socio-economic, education, health literacy, technological, cultural, political and environmental environments.
**Re-orientation of care**	**Description:** New and innovate health care solutions to improve coordination of health services and continuous health care; and intersectoral coordination.<br>**Data Items:** New technologies and strategies (eHealth; shared electronic medical records; telemedicine; m-health)
**Community engagement**	**Description:** Engaging and empowering individuals, families, communities and informal caregivers to facilitate common decision-making and self-efficacy. Reaching underserved and marginalised communities.
