# Supplementary material for: Towards universal health coverage for people with stroke in South Africa: a scoping review
Source: BMJ Open. 2021 Nov 24;11(11):e049988. doi: 10.1136/bmjopen-2021-049988 (PMC8627414; doi:10.1136/bmjopen-2021-049988)
Supplement: Supplementary data [file bmjopen-2021-049988supp003.pdf]

Supplementary file S3. Detailed demographic information of all included records (N=59)

S.no	Author (year)	Province	Area	Level of care	Literature	Aim	Study design	Sample characteristics
1	Arowoiya (2014)	Western Cape	Urban	Primary Healthcare (CHC)	Dissertations	To determine and explore the participation restrictions experienced by stroke patients	Mixed Methods (Survey + FGDs)	120 stroke patients receiving Physiotherapy for survey & 2 FGDs with 17 stroke patients
2	Bham & Ross 2005	Not reported	Undefined	Community	Primary Literature (peer reviewed publications)	To investigate the beliefs of caregivers and traditional healers within the South African Indian Muslim community regarding the etiology and treatment of stroke and the persons likely to be consulted in this regard	Descriptive case study design (Qualitative)	10 SAIM caregivers of people who had sustained strokes, as well as 10 SAIM traditional healers, who had treated stroke patients.
3	Biggs (2005)	Western Cape	Urban	Primary Healthcare (CHC)	Dissertations	To determine the health promotion needs of stroke patients accessing selected Community Health Centres in the Metropole region of the Western Cape.	Mixed Methods (Survey + In-depth interview)	418 stroke patients, representing each of the health districts of the Metropole region of the Western Cape for the survey and 12 stroke patients for Interviews
4	Biggs & Rhoda 2008	Western Cape	Urban	Primary Healthcare (CHC)	Primary Literature (peer reviewed publications)	To determine the health risk behaviours and factors that influence these behaviours of stroke patients in the Metropole Region of the Western Cape, South Africa	Mixed Methods (Survey + In-depth interview)	417 stroke patients – survey 12 stroke patients for Interviews
5	Blackwell & Littlejohns 2010	Gauteng,	Undefined	Undefined	Primary Literature (peer reviewed publications)	To measure the prevalence and review the assessment and management strategies related to dysphagia in three private rehabilitation clinics in South Africa	Review of records and thematic analysis	30 stroke patient records from three private rehabilitation clinics – total 90 records
6	Botha (2008)	Western Cape	Undefined	Community	Dissertations	To refine and pilot a training booklet for caregivers of stroke survivors for further	Mixed methods (Literature review +	Sample – 1: 11 Stroke patients in WCRC Sample – 2: 1Family members /other

						implementation	Checklist development + survey + FGDs)	carers of Stroke patients in WCRC<br>Sample – 3: 4 Home based carers and 22 nursing assistants attending carer training at WCRC<br>Sample – 4: 4 Stroke patients and their 4 caregivers participating in home-based care programme at university of Western Cape in Nyanga<br>Sample -5: Stroke patients in WCRC<br>Total 15 stroke patients and 31 caregivers
7	Bryer (2009)	South Africa	Undefined	Undefined	Primary Literature (peer reviewed publications)	There is an urgent need to develop a model of community-based stroke care with appropriate<br>Rehabilitation facilities and trained professionals<br>In South Africa, particularly in under-resourced<br>areas	Editorial	NA
8	Bryer et al (2010)	South Africa	NA	NA	Primary Literature (peer reviewed publications, Clinical Guideline)	The objective was to update the guideline published in 2000, to place the recommendations within the current South African context, and to grade evidence according to the level of scientific rigour for management of ischaemic stroke and transient ischaemic attack 2010	SASS writing committee Guidelines	NA
9	Burton 2016	South Africa	Undefined	Undefined	Primary Literature (peer reviewed publications)	To investigate the efforts of a woman with a talent for getting things done for bringing stroke units out of the blue for South Africa	Editorial	NA
10	Cawood 2012	Western Cape	Urban	Community	Dissertations	To determine if uninsured stroke survivors living in the Helderberg Basin (Western Cape) reached their optimal	A descriptive, mixed methods study (Surev + Interview)	53 stroke survivors (quantitative)<br>5 Stroke survivors (qualitative)

						rehabilitation outcome levels and if not, what environmental barriers contributed to this.		
11	Cawood& Visagie (2015)	Western Cape	Urban	Community	Primary Literature (peer reviewed publications)	To determine environmental barriers and facilitators to participation experienced by a group of stroke survivors in the Western Cape province of South Africa.	A descriptive, mixed methods study (Surev + Interview)	53 stroke survivors (quantitative)<br>5 Stroke survivors (qualitative)
12	Cawood & Visagie (2016)	Western Cape	Urban	Community	Primary Literature (peer reviewed publications)	To describe the functional outcomes achieved by stroke survivors in an urban Western Cape Province setting to add to the information on stroke management	A descriptive, mixed methods study (Surev + Interview)	53 stroke survivors (quantitative)<br>5 Stroke survivors (qualitative)
13	Cawood et al (2016)	Western Cape	Urban	Community	Primary Literature (peer reviewed publications)	To explore causal connections between impairments, activity limitations and participation restrictions after stroke.	Cross-sectional Study	53 stroke survivors
14	Connor (2005)	Sub Saharan Africa	Undefined	Undefined	Primary Literature (peer reviewed publications)	To understand the burden of stroke in black populations in sub-Saharan Africa	Systematic Review	All articles relevant to stroke in Sub Saharan Africa
15	Cunningham (2012)	Eastern cape	Urban	Uitenhage Provincial Hospital	Dissertations	To determine and explore the outcomes of stroke patients admitted to Uitenhage provincial hospital	Mixed Methods (quantitative survey + secondary data analysis + Semi-structured interviews)	168 stroke patient records for secondary data analysis, 24 stroke patients for prospective survey and 9 stroke patients for the qualitative study
16	Cunningham & Rhoda 2014	Eastern Cape	Urban	Community	Primary Literature (peer reviewed publications)	To determine the outcome of stroke patients in Eastern cape	Concurrent Mixed Methods design	24 Stroke Patients (Quantitative Survey)<br>9 Stroke patients (Qualitative interviews)
17	De la Cornillère (2007)	Western cape	Urban	BLRC Rehab centre	Dissertations	To describe the range of experiences of stroke patients relating to attendance or non-attendance of those referred to the Bishop Lavis Rehabilitation centre stroke group	Mixed Methods Descriptive study	20 participants with stroke for questionnaire survey and 6 stroke participants for FGD.

18	De Villiers et al 2009	Cape Town	Urban	Secondary hospital	Primary Literature (peer reviewed publications)	To examine the impact of multidisciplinary stroke care on the in-hospital mortality, resource utilization, and access to inpatient rehabilitation facilities for stroke patients admitted in Stroke units at a secondary hospital in Cape Town, South Africa	Cross-sectional pre and post study design	195 stroke patients
19	De Villiers 2011	Cape Town	Urban	District hospital	Primary Literature (peer reviewed publications)	To determine survival, disability and functional outcomes of stroke patients following their discharge from an acute stroke unit in an urban community with limited rehabilitative resources	Cross-sectional pre and post study design	196 stroke patients
20	Elloker (2015)	Western Cape	Urban	CHC	Dissertations	To determine participation restrictions and social support in patients with stroke, living in the Western Cape.	Mixed methods (Systematic Review + Quantitative survey)	106 stroke patients
21	Faux (2006)	Non-specific	Undefined	Undefined	Primary Literature (peer reviewed publications)	To provide a practical guide to helping stroke survivors who have a persistent disability maintain and enhance the gains made in rehabilitation	Narrative review recommendation	NA
22	Felemengas (2005)	Johannesburg Gauteng	Urban	Academic hospital	Dissertations	To investigate the family dynamics within the family system, as well as how these have evolved or changed following a stroke.	Qualitative	6 primary caregivers of stroke survivors
23	Groenewald & Rhoda 2017	Western Cape	Urban	Non-Governmental facility	Primary Literature (peer reviewed publications)	To determine outcomes of stroke patients managed by a multidisciplinary team at a step-down facility in the Western Cape.	A longitudinal observational study	68 stroke patients
24	Groenewald (2018)	Western Cape	Urban	Step down rehabilitation facility	Dissertations	To adapt and contextualize the original UK Bridges stroke SMI workbook for implementation with the South African stroke	A qualitative exploratory study -Interview, FGD, Expert	13 Health care professionals<br>12 Stroke patients<br>Expert Panel

						population	consultation	
25	Hassan et al (2011)	Western cape	Urban	Western Cape Rehabilitation centre	Primary Literature (peer reviewed publications)	To explore levels of strain experienced by caregivers and the variables that impact on their strain.	Concurrent, mixed method, descriptive design	57 caregivers of stroke survivors
26	Hilton (2011)	Johannesburg	Urban	Community	Dissertations	To establish the functional level of patients, the level of strain and quality of life of the caregiver six to 36 months post-stroke, and the influence of demographic factors, caregiver strain and patient's functional ability on quality of life of the caregiver.	Cross-sectional study	35 stroke patients and their caregivers
27	Hossain (2016)	Kwa-Zulu-Natal	Urban	Ladysmith Regional Hospital	Dissertations	To investigate the Need for Palliative care in Cerebrovascular Accident (stroke) patients at Ladysmith Regional Hospital	Mixed Methods (qualitative and quantitative)	72 stroke patients for quantitative study and<br>10 stroke patients for qualitative study
28	Joseph 2012	Western cape	Urban	WCRC – Rehabilitation centre	Dissertations	To determine the process of rehabilitation and the outcome of patients following in-patient rehabilitation at a facility in the Western Cape	A descriptive, observational, longitudinal design	76 Spinal Cord Injury patients and 67 stroke patients. Total patients (including drop outs) 130.
29	Kleineibst (2007)	Western Cape	Urban	Community	Dissertations	To determine the effectiveness of a caregiver support intervention programme to address the need for primary caregivers of stroke survivors in Bishop Lavis Community	Prospective descriptive qualitative study	29 caregivers of stroke survivors
30	Kotsokoane et al (2018)	Gauteng	Urban	CHC	Primary Literature (peer reviewed publications)	To determine the level of integration of stroke survivors at Soshanguve community clinics.	Retrospective quantitative research design	114 stroke survivors
31	Kusambiza-Kiingi (2016)	Johannesburg Gauteng	Urban	CHC	Primary Literature (peer reviewed publications)	To determine stroke survivors' levels of community reintegration, quality of life (QOL),<br>satisfaction with the physiotherapy services and the	Cross-sectional study	108 stroke survivors and 45 caregivers

						level of caregiver strain at community health centres within the Johannesburg		
32	Leichtfuss (2009)	Western Cape	Urban	Private acute care hospitals	Dissertations	To examine the practice of doctors with regards to stroke rehabilitation in private acute-care hospitals private acute-care hospitals and to evaluate information shared between doctors and pts i.r.t prognosis, severity, discharge, referral, timing of discharge planning and decision making. in the Western Cape Metropole	Retrospective and descriptive design	37 doctors treating and discharging stroke patients 48 pts
33	Mabunda (2015)	Western Cape	Urban	Private not for profit Intermediate care Facility	Dissertations	To describe the model of service provision at an IC facility and the role it plays in the continuity of care in Cape Town.	Cross sectional survey	68 stroke patients 70 clinical staff
34	Makganye (2015)	Gauteng?	Urban	CHC	Dissertations	To investigate the physical, psychological, social, religious	Qualitative design	5 stroke patients and 5 caregivers
35	Maleka et al (2012)	Limpopo and Gauteng	Urban and rural	Community	Primary Literature (peer reviewed publications)	To establish the experience of people living with stroke in low socioeconomic urban and rural areas of South Africa	Qualitative study design	32 stroke survivors living in the community
36	Mamabolo et al (2008)	Gauteng	Urban	PHC clinics	Primary Literature (peer reviewed publications)	to establish what demographic, environmental and physical factors influence functional independence post stroke.	Cross-sectional study	68 stroke patients
37	Mandizvidza (2017)	Western Cape	Urban	Level 1 2 3 hospitals	Dissertations	To describe the acute and post-acute ischaemic stroke services offered to ischaemic stroke patients in level 1, 2, and 3 hospitals in the Cape Metro Health District, compare these services to the national guideline and identify any barriers to optimum stroke	Descriptive cross-sectional study	10 doctors and 10 nurses from stroke ward and 8 doctors from emergency ward; pt records

						patient care.		
38	Mashau et al 2016	Limpopo	Rural and Urban	HBC organization	Primary Literature (peer reviewed publications)	To investigate the impact of caregiving on voluntary home-based caregivers.	A quantitative cross-sectional descriptive survey	190 home-based caregivers
39	Masuku et al (2018)	Gauteng	Urban	Community	Primary Literature (peer reviewed publications)	To describe the caregiving experience of female caregivers of PWA residing in Tembisa, a township situated in the east of Johannesburg	Qualitative study	14 primary caregivers of stroke survivors with Aphasia
40	Matshikiza (2019)	Western cape	Urban	Tertiary Hospital	Dissertations	to determine the pre-hospital barriers and in-hospital delays to emergency care for patients presenting to Groote Schuur Hospital (GSH) with acute stroke.	prospective, observational study	50 patients with stroke
41	Mudzi (2010)	Gauteng	Urban	Community	Primary Literature (peer reviewed publications)	To establish the impact of caregiver education on the morbidity of the stroke survivors and on the quality of life of the stroke survivors and their carers.	A stratified randomised controlled trial	200 stroke patients and caregivers
42	Mudzi et al (2013)	Gauteng	Urban	Community	Dissertations	to establish the extent of community participation and the barriers and facilitators to the participation for stroke patients after their discharge.	longitudinal study	200 patients with first-time ischaemic stroke
43	Ntamo (2011)	Eastern Cape	Urban	Mthatha General Hospital	Dissertations	To identify factors that influence poor attendance for outpatient physiotherapy by patients discharged from MGH with a stroke.	Mixed methods (Qualitative + Quantitative study)	85 stroke patients attending Physiotherapy at MGH
44	Parekh and Rhoda (2013)	Western Cape	Urban	Tertiary hospital	Primary Literature (peer reviewed publications)	To determine functional outcomes and factors influencing functional outcomes of stroke patients admitted to a South African tertiary hospital	Longitudinal Pre and Post test design	100 stroke patients
45	Parekh	Western	Urban	Tertiary	Dissertations	to identify factors influencing	A descriptive,	66 stroke patients

	(2011)	Cape		hospital		functional outcome of stroke patients admitted to a South African tertiary hospital	observational, longitudinal quantitative study design	
46	Posner (2015)	Gauteng	Urban	Community	Dissertations	to explore the experiences and perceived needs of employed caregivers working for patients who have suffered from a stroke within home settings in South Africa.	Qualitative research design with Interviews + FGDs	15 employed caregivers working at the homes of stroke survivors FGDs with 10 participants 5 in each group
47	Ras (2009)	Western Cape	Urban	NGO Run Hospital booth	Dissertations	to assess the quality of the stroke rehabilitation services at Booth Memorial Hospital.	Cross-sectional audit of records	NA
48	Rhoda (2009)	Western Cape	Peri - Urban	CHC	Primary Literature (peer reviewed publications)	to determine the structure and process of rehabilitation of stroke patients at Community Health Centres (CHCs) in the Western Cape	Quantitative cross-sectional survey	100 first time stroke patients and 16 therapists
49	Rhoda et al (2011)	Western Cape	Peri - Urban	CHC	Primary Literature (peer reviewed publications)	to determine the activity limitations of stroke patients receiving rehabilitation at out-patient Community health Centres (ChCs)	Longitudinal observational study	100 patients with stroke
50	Rhoda (2014)	Western Cape	Peri - Urban	CHC	Primary Literature (peer reviewed publications)	to determine the quality of life and factors influencing quality of life of community-dwelling stroke patients living in low-income, peri-urban areas in the Western Cape, South Africa.	Observational, longitudinal study	100 first time stroke patients
51	Rhoda et al (2015)	South Africa (Eastern Cape) Tanzania and Rwanda	Undefined	Provincial hospital	Primary Literature (peer reviewed publications)	the provision of inpatient rehabilitation and the post discharge challenges of stroke survivors in specific African countries.	Retrospective survey and interviews	168 SA stroke patients 145 Tanzanian and 130 Rwandan stroke patients 9 SA patients, 10 TP and 10 RP for qualitative study
52	Rouillard et al (2012)	Western Cape	Urban	WCRC rehab centre	Primary Literature (peer reviewed publications)	To determine activity limitations, participation restrictions, health-related quality of life and caregiver strain in community-dwelling	Longitudinal and descriptive study	46 stroke patients 41 caregivers

						stroke survivors discharged from an intensive inpatient rehabilitation programme at 6 months post stroke.		
53	SA-CSRG 2019	National	NA	NA	Primary literature (Peer-reviewed, Clinical Guideline)	contextualised development of stroke rehabilitation guideline	Guideline	NA
54	Scheffler and Mash 2019	Western Cape	Rural	Community	Primary Literature (peer reviewed publications)	To describe and analyze the outcomes of patients with stroke from a rural PHC setting in the Western Cape, South Africa.	Longitudinal survey	93 stroke patients
55	Smith (2019)	Western Cape	Western Cape	Urban and Rural	Community	To explore the self-management strategies employed by stroke survivors in the Western Cape, South Africa	Exploratory qualitative design (In-depth Interviews)	14 stroke survivors
56	Taylor & Ntusi (2019)	South Africa	South Africa	Undefined	Undefined	To improve management of stroke in South Africa	Editorial (Review)	NA
57	Thomas & Greenop (2008)	Gauteng	Gauteng	Urban	Community	To investigate into the complexities of caregiving, including both perceptions and experiences of the healthcare system.	Qualitative design (interviews)	6 caregivers of stroke survivors
58	Viljoen (2014)	Western Cape	Western Cape	Urban	Groote Schuur Hospital	To determine the cost of stroke care and to identify factors associated with increased expense, as well as to evaluate the quality of stroke care in general medical wards	Review of records	261 stroke patient (records)
59	Wasserman et al 2009	KwaZulu-Natal	KwaZulu-Natal	Rural	Community	To assess discharge planning of stroke patients and to evaluate integration and continuity of stroke care between hospital and community	Quantitative design (Survey)	30 stroke patients
