# Supplementary material for: Towards universal health coverage for people with stroke in South Africa: a scoping review
Source: BMJ Open. 2021 Nov 24;11(11):e049988. doi: 10.1136/bmjopen-2021-049988 (PMC8627414; doi:10.1136/bmjopen-2021-049988)
Supplement: Supplementary data [file bmjopen-2021-049988supp004.pdf]

Supplementary file S4: Components assessed in included studies

Author (year)	Governance /Regulation	Resources	Service Delivery	Context	Re-organisation of care	Community engagement
Arowoiya (2014)			X	X		
Bham & Ross 2005			X	X		
Biggs (2005)		X	X	X		X
Biggs & Rhoda (2008)				X		
Blackwell & Littlejohns (2010)			X			
Botha (2008)					X	X
Bryer (2009)		X	X			
Bryer et al (2010)	X					
Burton 2016					X	X
Cawood 2012		X	X			X
Cawood& Visagie (2015)			X			X
Cawood & Visagie (2016)			X			X
Cawood et al (2016)						x
Connor (2005)	X	X		X		
Cunningham (2012)		X	X	X		X
Cunningham & Rhoda (2014)						X
De la Cornillère (2007)			X	X		X
De Villiers et al (2009)			X			
De Villiers (2011)		X	X			
Elloker (2015)						X
Faux (2006)					X	
Felemengas (2005)			X	X		X
Groenewald & Rhoda (2017)			X			
Groenewald (2018)			X		X	
Hassan et al (2011)				X		
Hilton (2011)			X	X		
Hossain (2016)			X		X	
Joseph (2012)			X			X
Kleineibst (2007)		X	X	X		
Kotsokoane et al (2018)			X			X
Kusambiza-Kiingi (2016)			X	X		
Leichtfuss (2009)		X	X			X
Mabunda (2015)			X			
Makganye (2015)		X	X	X		
Maleka et al (2012)		X		X		X
Mamabolo et al (2008)						X
Mandizvidza (2017)	X	X	X			
Mashau et al (2016)			X			
Masuku et al (2018)			X	X		
Matshikiza (2019)		X	X			
Mudzi (2010)			X	X		X
Mudzi et al (2013)			X	X		

Ntamo (2011)		X	X	X		X
Parekh and Rhoda (2013)			X			
Parekh (2011)			X			
Posner (2015)			X			
Ras (2009)		X	X			
Rhoda (2009)		X	X			
Rhoda et al (2011)			X			
Rhoda (2014)			X	X		
Rhoda et al (2015)			X	X		
Rouillard et al (2012)			X	X		
SA-CSRG	X					
Scheffler and Mash (2019)			X			
Smith (2019)			X	X		
Taylor & Ntusi (2019)			X			
Thomas & Greenop (2008)			X	X		
Viljoen (2016)		X	X			
Wasserman et al (2009)			X			
**Total**	**4**	**16**	**47**	**24**	**5**	**19**

\]
