# Supplementary material for: Towards universal health coverage for people with stroke in South Africa: a scoping review
Source: BMJ Open. 2021 Nov 24;11(11):e049988. doi: 10.1136/bmjopen-2021-049988 (PMC8627414; doi:10.1136/bmjopen-2021-049988)
Supplement: Author's manuscript [file bmjopen-2021-049988.draft_revisions.pdf]

BMJ Open

BMJ Open is committed to open peer review. As part of this commitment we make the peer review history of every article we publish publicly available.

When an article is published we post the peer reviewers' comments and the authors' responses online. We also post the versions of the paper that were used during peer review. These are the versions that the peer review comments apply to.

The versions of the paper that follow are the versions that were submitted during the peer review process. They are not the versions of record or the final published versions. They should not be cited or distributed as the published version of this manuscript.

BMJ Open is an open access journal and the full, final, typeset and author-corrected version of record of the manuscript is available on our site with no access controls, subscription charges or pay-per-view fees (http://bmjopen.bmj.com).

If you have any questions on BMJ Open's open peer review process please email info.bmjopen@bmj.com

BMJ Open

Towards universal health coverage for people with stroke in South Africa: a scoping review

Journal:	*BMJ Open*
Manuscript ID	bmjopen-2021-049988
Article Type:	Original research
Date Submitted by the Author:	08-Feb-2021
Complete List of Authors:	Sjan-Mari, van Niekerk; Stellenbosch University, Division of Physiotherapy, Department of Health and Rehabilitation Sciences<br>Sureshkumar, K; Public Health Foundation of India, SACDIR; London School of Hygiene & Tropical Medicine, International Center for Evidence in Disability<br>Gakeemah, Inglis-Jassiem; Stellenbosch University, Division of Physiotherapy, Department of Health and Rehabilitation Sciences<br>Charumbira, Maria; Stellenbosch University Faculty of Medicine and Health Sciences, Rehabilitation Sciences<br>Fernandes, Silke; London School of Hygiene and Tropical Medicine,<br>Webster, Jayne; London School of Hygiene and Tropical Medicine,<br>English, Rene; Stellenbosch University Faculty of Medicine and Health Sciences, Global Health<br>Louw, QA; Stellenbosch University, Physiotherapy; Stellenbosch University,<br>Smythe, Tracey; London School of Hygiene and Tropical Medicine, Clinical Research
Keywords:	Stroke < NEUROLOGY, PUBLIC HEALTH, Health policy < HEALTH SERVICES ADMINISTRATION & MANAGEMENT, Rehabilitation medicine < INTERNAL MEDICINE, PRIMARY CARE

SCHOLARONE™
Manuscripts

BMJ

I, the Submitting Author has the right to grant and does grant on behalf of all authors of the Work (as defined in the below author licence), an exclusive licence and/or a non-exclusive licence for contributions from authors who are: i) UK Crown employees; ii) where BMJ has agreed a CC-BY licence shall apply, and/or iii) in accordance with the terms applicable for US Federal Government officers or employees acting as part of their official duties; on a worldwide, perpetual, irrevocable, royalty-free basis to BMJ Publishing Group Ltd ("BMJ") its licensees and where the relevant Journal is co-owned by BMJ to the co-owners of the Journal, to publish the Work in this journal and any other BMJ products and to exploit all rights, as set out in our licence.

The Submitting Author accepts and understands that any supply made under these terms is made by BMJ to the Submitting Author unless you are acting as an employee on behalf of your employer or a postgraduate student of an affiliated institution which is paying any applicable article publishing charge ("APC") for Open Access articles. Where the Submitting Author wishes to make the Work available on an Open Access basis (and intends to pay the relevant APC), the terms of reuse of such Open Access shall be governed by a Creative Commons licence – details of these licences and which Creative Commons licence will apply to this Work are set out in our licence referred to above.

Other than as permitted in any relevant BMJ Author's Self Archiving Policies, I confirm this Work has not been accepted for publication elsewhere, is not being considered for publication elsewhere and does not duplicate material already published. I confirm all authors consent to publication of this Work and authorise the granting of this licence.

For peer review only

Towards universal health coverage for people with stroke in South Africa: a scoping review

Sjan-Mari van Niekerk,[1] *Sureshkumar Kamalakannan ,[2,3] Gakeemah Inglis-Jassiem,[1] Maria Y Charumbira[1], Silke Fernandes,[4] Jayne Webster,[5] René English,[6] Quinette A Louw,[1] Tracey Smythe[3]

*Corresponding Author

Dr Sureshkumar Kamalakannan
International Center for Evidence in Disability,
London School of Hygiene and Tropical Medicine, London, UK
suresh.kumar@lshtm.ac.uk

1 Division of Physiotherapy, Department of Health and Rehabilitation Sciences, Stellenbosch University, Cape Town, South Africa

2 Public Health Foundation of India, Indian Institute of Public Health Hyderabad, SACDIR, India

3 International Center for Evidence in Disability, London School of Hygiene and Tropical Medicine, London, UK

4 Department of Global Health and Development, Faculty of Public Health and Policy, London School of Hygiene and Tropical Medicine, London, UK

5 Department of Disease Control, Faculty of Infectious and Tropical Diseases, London School of Tropical Health and Medicine, London, UK

6 Division of Health Systems and Public Health, Department of Global Health, Faculty of Medicine and Health Sciences, Stellenbosch University, Cape Town, South Africa

For peer review only

Total word count – 4566 without abstract, tables, figure and references

Key words – Stroke; Universal Health Coverage; Health System; South Africa; Scoping Review

For peer review only - http://bmjopen.bmj.com/site/about/guidelines.xhtml

ABSTRACT – word count 284

Objectives: To explore the opportunities and challenges within the South African health system to facilitate the achievement of UHC for people with stroke.

Setting: South Africa

Design: Scoping Review

Search Methods: We conducted a scoping review of opportunities and challenges to achieve UHC for PWS in the SA health system. Global and Africa specific databases and grey literature were searched in July 2020. Studies of all designs were included if they described any aspect of the health care system for PWS. Two frameworks, the Health Systems Dynamics Framework and WHO Framework on integrated people-centred health services, were used to map data and a narrative approach was used to synthesise results.

Results

Sixty studies were included in the review. Over half (51.6%) were conducted in Western Cape and most (68.3%) were conducted in urban areas. The nature, extent, and distribution of data in SA on stroke care in terms of governance and regulation, resources, service delivery, context, re-orientation of care and community engagement were found to be limited. A key finding was a lack of adequate evidence on governance and regulations for stroke care in terms of government support, investment in policy, treatment guidelines, resource distribution and commitment to evidence-based solutions. Service delivery factors for stroke care were frequently reported as poor, compounded with context related limiting factors. Promising supporting factors included adequately equipped and staffed urban tertiary facilities.

Conclusion

This review highlights the multifactorial nature of the weaknesses in the SA healthcare system and indicates the lack of readiness for UHC for PWS, especially in terms of adequate governance and regulations. An important factor to attain UHC for PWS is to prioritize and include this marginalised group into the proposed national health insurance scheme.

For peer review only - http://bmjopen.bmj.com/site/about/guidelines.xhtml

STRENGTHS AND LIMITATIONS OF THIS STUDY

- A comprehensive search strategy was developed, and the search was carried out in global, national and continental specific databases.
- The scoping review was systematic with a robust methodology that included double data extraction and data review to synthesise the state of the evidence on the topic.
- The use of a combination of frameworks such as the Health Systems Dynamics and Integrated People-Centred Health Services contributed to rigorous evaluation.
- There was no limitation on study design or exclusion based on methodological appraisal for the inclusion of records.
- Comparison of studies were challenged by heterogeneity especially regarding design and aim.

For peer review only

INTRODUCTION

Stroke is a leading cause of death and disability worldwide (1). In South Africa (SA) stroke is the second most common cause of death after HIV/AIDS and a significant cause of morbidity (2–5). It is estimated that 75 000 people experience stroke each year in SA, contributing to 564 000 stroke-related disability-adjusted-life-years (6). Furthermore, stroke incidence in rural areas of SA is increasing; an estimated 33,500 strokes occurred in these areas in 2011, contributing to half of the national stroke burden (7). However, these data are likely underestimated due to the absence of a national stroke database or registry and the paucity of studies that were undertaken in a few parts of the country.

Stroke is the leading cause of disability in adults in SA, placing strain on social and health services (8). Increased prevalence of heart disease, hypertension, diabetes mellitus, behavioural factors such as smoking, and structural factors such as unchecked industrialisation and urbanisation, contribute to this epidemiological transition of stroke in many low and middle-income countries (LIMCs)(9), including SA (2). The SA government has committed to the World Health Organisation (WHO) vision of achieving equitable, evidence-based rehabilitation for all by 2030 (10). However, it is not clear how many people access rehabilitation services following stroke, what this rehabilitation entails, and how effective this rehabilitation is (11). Therefore, achieving key global health targets and development goals will be challenging, including Universal Health Coverage (UHC) (12,13).

UHC is achieved when every person receives essential services, such as health promotion, prevention, treatment, rehabilitation (including assistive technology) and palliative care, according to their needs and without financial hardship (14). Accessible, responsive and quality stroke care services within a strengthened local health system will contribute to UHC for PWS in SA. The extent to which UHC is currently achieved for PWS in SA is unknown (15). We aimed to describe the health system related factors that will facilitate UHC for PWS and the shortcomings that currently limit implementation of UHC for stroke care in SA.

METHODS

A scoping review was conducted according to the five-step approach recommended by Levac et al.(16) as outlined in our published protocol (17): 1) identifying the research question, 2) identifying relevant studies, 3) selecting the studies, 4) charting the data, and 5) collating,

For peer review only - http://bmjopen.bmj.com/site/about/guidelines.xhtml

summarising and reporting the results. The results are reported according to the Preferred Reporting Items for Systematic Reviews and Meta-Analyses extension for Scoping Reviews (PRISMA-ScR) guidelines (18).

Patient and public involvement

No patients and/or public were involved in the design, conduct, reporting, or dissemination plans of this research.

Analytical framework

This review was guided by an analytical framework adapted from the Health Systems Dynamics Framework (HSDF)(19) and WHO Framework on integrated people-centred health services (IPCHS)(20). Our analytical framework includes all the HSDF components and two components from the IPCHS: 1) Re-orientation of care and 2) Enabling environment, which are appropriate to the SA context and population (Figure 1). 'Resources' and 'Enabling environment' were combined and titled 'Resources' as the data items described under each were similar.

Figure 1: Framework components

Identifying the research question

To answer the question 'what are the opportunities and challenges within the SA health system to facilitate achieving UHC for people with stroke?' the review objectives were to:

1. describe the health system related factors that support and guide achieving universal stroke care in SA
2. describe the health system related factors that limit achieving universal stroke care in SA
3. identify driving factors with potential to bring change required to achieve universal stroke care in SA.

Identifying relevant studies

In line with the purpose of scoping reviews, our approach was broad, with emphasis on studies that investigated any aspect of the health care system regarding stroke care in SA.

For peer review only - http://bmjopen.bmj.com/site/about/guidelines.xhtml

Search Strategy

We conducted a comprehensive search, according to the methodology described in our published protocol (17). Grey literature was identified through the National Electronic Thesis and Dissertation portal, and websites of relevant government and service provider agencies. Field experts were contacted to identify additional relevant evidence regarding stroke care in SA. Saturation was the point at which no new records were found for inclusion.

Eligibility criteria

Full text, SA based studies on stroke care of any design that addressed at least one framework component were included (17).

Evidence selection

Two reviewers (SvN, SK) independently screened the titles and abstracts of identified studies. A third reviewer (GIJ) checked the results for accuracy. Results of the initial screening were compared, and full-text records obtained for articles deemed eligible by at least one reviewer. Two reviewers (SvN, SK) independently screened the full texts using the eligibility criteria. Any discrepancies were resolved by discussion with a third reviewer (GIJ). Data were managed with Covidence (https://support.covidence.org/help) and Excel (version 365).

Data charting

The six framework components were divided between three reviewers (SvN; SK; MC) who extracted, collated, and summarised relevant data into a purpose-built Microsoft Excel database. We considered the six components using the descriptions as outlined in Supplementary file 1 (S1) and data on the following study components were extracted:

- General study information, including author and year of publication
- Study design, sampling, and recruitment methods
- Study settings and dates conducted
- Population characteristics
- Study measures
- Research outcomes related to the framework components

The three reviewers compared their results and reached consensus on organisation of extracted data. The final data and analysis were evaluated by a research team member (TS), to ensure that interpretations were credible and valid.

For peer review only - http://bmjopen.bmj.com/site/about/guidelines.xhtml

Data synthesis and analysis

We summarised the study characteristics and the study designs. We used a framework analysis approach to deductively analyse data of the included studies, which consisted of five key steps as described by Ritchie et al. (21). The framework in figure 1 was used as a dynamic tool to aid this synthesis and data was managed with Atlas.ti (version 8) and Microsoft Excel (version 365).

The final synthesis of themes was confirmed following critical discussion between all the authors. We undertook a narrative synthesis of the findings, highlighting supporting and limiting factors to achieving health for all PWS in SA. The extent of evidence supporting each component of the framework as a hinderance of UHC was synthesised and included in the framework diagram.

RESULTS

We identified a total of 4,133 records and screened the abstracts of 508. After reviewing 75 full-text records, we included a total of 60 full texts in our review. A PRISMA flow diagram summarised the study selection process (Figure 2).

Figure 2: PRIMSA flow chart

Study characteristics

Most studies (n=31, 51.6%) were undertaken in the Western Cape province. No studies were found from four of the nine provinces in SA (Free State, Mpumalanga, Northern Cape, or the North West Provinces). The most common study design was quantitative (n=22, 46.6%), followed by mixed methods (n=14, 23.3%) and qualitative (n=10, 16.6%). Eighteen (30%) studies were community-based whilst the remaining studies recruited participants from clinics (n=12, 20%) or hospitals (n=16, 26.6%). The most commonly reported framework component was Service Delivery and (n=47, 78%) and the least reported was Governance and Regulation (n=4, 6%) (Table 1).

Supplementary file 2 (S2) provides a detailed summary of included records and supplementary file 3 (S3) provides information on components reported per included record.

For peer review only - http://bmjopen.bmj.com/site/about/guidelines.xhtml

Table 1: Characteristics of included records (N=60)

Variable	Category - N (%)
Province	Western Cape – 31 (51.6%)<br>Gauteng – 12 (20.0%)<br>National- 7 (11.6 %)<br>Eastern Cape – 4 (6.6%)<br>KwaZulu-Natal – 2 (3.3%)<br>Limpopo – 1 (1.6%)<br>Limpopo and Gauteng – 1 (1.6%)<br>Free State – 0 (0%)<br>Mpumalanga – 0 (0%)<br>Northern Cape – 0 (0%)<br>North West – 0 (0%)<br>Undefined – 2 (3.3%)
Area	Urban – 41 (68.3%)<br>Rural and Urban – 3 (5.0%)<br>Peri-Urban – 3 (5.0%)<br>Rural – 2 (3.3%)<br>Undefined – 11 (18.3%)
Levels of care	Community – 18 (30.0%)<br>Hospital – 16 (26.6%)<br>Primary Healthcare (Clinics; Community Health Centres) – 12 (20.0%)<br>Rehabilitation centres – 6 (10.0%)<br>Undefined – 8 (13.3%)
Study design	Quantitative - 28 (46.6%)<br>Mixed methods – 14 (23.3%)<br>Qualitative measures – 10 (16.6%)<br>Review – 3 (5%)<br>Editorial – 3 (5%)<br>Guideline – 2 (3.3%)
Record	Primary Literature (publications) – 35 (58.3%)

For peer review only - http://bmjopen.bmj.com/site/about/guidelines.xhtml

description	Grey Literature: Dissertations – 25 (41.6%)							
Included population	PWS – 34 (56.6 %)<br>Editorials and reviews – 9 (15.0%)<br>Caregiver – 6 (10.0 %)<br>PWS + Caregiver – 5 (8.3 %)<br>PWS + HCP – 3 (5.0 %)<br>HCP – 1 (1.6 %)<br>PWS + HCP+ Experts – 1 (1.6 %)<br>Traditional healers + Caregivers – 1 (1.6 %)<br>Policy makers – 0 (0 %)							
Framework components	**CE**	**Con**	**GR**	**ReO**	**Res**	**SD**	**n**	**%**
						x	12	20.0%
		x				x	12	20.0%
					x	x	5	8.3%
	x	x			x	x	4	6.6%
	x						4	6.6%
	x					x	4	6.6%
	x	x				x	3	5.0%
				x		x	2	3.3%
		x			x	x	2	3.3%
	x			x			2	3.3%
			x				2	3.3%
		x					2	3.3%
	x				x	x	2	1.6%
				x			1	1.6%
			x		x	x	1	1.6%
		x	x		x		1	1.6%
	x	x			x		1	1.6%
							**60**	**100%**
	**19**	**24**	**4**	**5**	**16**	**47**		

For peer review only - http://bmjopen.bmj.com/site/about/guidelines.xhtml

*SSA = Sub- Saharan Africa; PWS= Person with Stroke; HCP = Health Care Professional; NA = Not Applicable; GR = Governance and Regulations; SD = Service delivery; Res = Resources; CE = Community Engagement; Con = Context; ReO = Re-Organisation of Care

Service Delivery

Comprehensiveness

Multi-disciplinary teams (MDT), consisting of five or more health care professionals were reported in nine studies (22–30). Two studies indicated that MDTs were either absent, limited, or inefficient (31,32).

Continuity of Care

Continuity of care was limited by poor referral, bed capacity for inpatient care, coordination, communication (among healthcare providers and with patients) and follow-up systems. One study indicated that poor understanding of traditional medicine and lack of trust may hinder adequate stroke care (33). At community level, referral to support groups lacked co-ordination and stroke survivors lacked knowledge of care options (26,34–36). Two studies conducted in the rural Western Cape reported that 30-40% of patients did not receive home-based care (2,37). Waiting-time for investigations and care was lengthy (31,35,38). Findings included delays in investigations being associated with a significant increase in length of stay (LOS)(39) and doctor-centric models delaying investigations or treatments (37,40,41).

Timeliness of Care

Bed shortages (27,32,35,38,42) and the pressure to discharge patients in hospitals precluded rehabilitation and delayed post-discharge rehabilitation (28,32,43,44). Four studies (28,43–45) reported that patients were discharged when medically stable (average stay was 5-10 days at secondary or tertiary hospitals (27,36,43,46)) despite functional deficits (26,27,43,47,48). Cunningham (2012) (44) reviewed 168 stroke patient records from the Eastern Cape and found only 15% were referred for physiotherapy on the day or day prior to discharge (44). Over weekends, 13% of patients did not receive any therapy (44). Difficulty with securing follow-up appointments and cancellations influenced the timeliness of post- discharge care (2,31,37,49,50).

Quality of Care

Three studies conducted in the Western Cape found that patients received between one and five rehabilitation sessions, except for the specialised Rehabilitation Centre where patients typically received 17 sessions (25,28,51,52), LOS was typically 5 - 10 days and

For peer review only - http://bmjopen.bmj.com/site/about/guidelines.xhtml

approximately 30 days in rehabilitation facilities (39,53–56). One study reported that prompt assessment by rehabilitation professionals was associated with shorter LOS (39).

Perceptions of Care

Conflicting evidence exists in regards to perceptions of care with ten studies reporting positive staff attitudes (29,31,37,41,56–61) while nine studies reported negative staff behaviour and attitudes (30,33,37,49,57,62–65). A further four studies found that PWS were dissatisfied with the healthcare service, which was driven by lack of information about their treatment and further referral (6,31,54,65). Leichtfuss (2009) (30) highlighted the significant discrepancy (p -value = 0.00438) between doctors' understanding and patients' perception of the effectiveness of the doctors' communication; 80% (n=28) of doctors compared to 50% (n=24) of patients thought that sufficient information was communicated (30). The study also found that patients perceived nursing services as inefficient and inadequate, which was supported by doctors who expressed the need for nursing staff who were trained in stroke care (30). Caregiver support and training was lacking (36,62,64,66) and resulted in care-giver burnout (63). Caregivers indicated need for additional training and help, particularly with toileting and bath transfers, and requested more home visits by therapists (36). Table 2 outlines measures and study findings that target Service Delivery.

Table 2: Description of measures and study findings that target Service Delivery (N=47).

**Author (year)**	**Service Delivery**
**Comprehensiveness of Care**	
Supporting factors	
Groenewald and Rhoda (2017); Rhoda et al. (2015); Joseph (2012); Rouillard et al., (2012); Leichtfuss (2009) Ras (2009); Wasserman et al, (2009); Rhoda (2009); De la Cornillère (2007)	Comprehensive MDTs consisting of five or more healthcare professionals in Western Cape province
Limiting factors	
Cawood (2012); De Villiers (2011)	Limited/absent MDT team consisting of less than five different healthcare professionals
**Continuity of Care***	
Limiting factors	
Masuku (2018); Mandizvidza (2017); Cawood & Visagie (2016); Joseph (2012); De la Cornillère (2007); Kleinheibst (2007)	Poor referral pathways (community; hospital)
Rhoda (2014); Rouillard (2012); Bham & Ross (2005); Scheffler and Mash (2019);	Poor follow-up and referral post discharge
Bham & Ross (2005)	Lack of reciprocal respect and understanding

For peer review only - http://bmjopen.bmj.com/site/about/guidelines.xhtml

	and coordination between traditional and medical healthcare professionals
**Timeliness of Care***	
Limiting factors	
Cawood (2012); Mudzi (2013)	Long queues in hospitals, community health clinics, and outpatient clinics
Arowoiya (2014)	Long waiting times for follow-up appointments
Matshikiza (2019); Mandizvidza (2017); Parekh & Rhoda (2013); Cawood (2012); Bryer (2009)	Long waiting times for inpatients to receive specialised health services
Cawood & Visagie (2015); Cawood (2012)	Doctor-centric model of care
Cawood (2012); Parekh (2011)	Poor collaboration between health care providers
Cunningham (2012); Hilton (2011); De Villiers (2009); Rhoda (2009)	Inadequate rehabilitation during hospital stays
**Quality of Care**	
Supporting factors	
Viljoen (2014)	Prompt assessment by an allied health professional significantly decreases LOS
Limiting factors	
Mandizvidza (2017); Leichfust (2009); Ras (2009)	Lack of appropriate care due to lack of stroke specific knowledge
Groenewald & Rhoda (2017); Parekh (2011); Rhoda et al (2011); Rhoda (2009)	Low number of in-hospital rehabilitation sessions
Groenewald (2018); Mabunda (2015); Rhoda (2014); Viljoen (2014); Hilton (2011); Parekh (2011); Blackwell & Littlejohn (2010); Mudzi (2010); Ras (2009); Kleinhebst (2007); Felemengas (2004)	Inadequate LOS at all levels of care except for specialist rehabilitation facilities
**Perceptions of Care**	
Supporting factors	
Taylor & Ntusi (2019); Groenewald (2018); Kotsokoane (2018); Hossain (2016); Kusambiza-Kiingi (2016); Cawood & Visagie (2015); Makganye (2015); Cawood (2012); Ntamo (2011); De la Cornillère (2007)	Positive staff attitudes and care
Limiting factors	
Smith (2019); Cawood & Visagie (2015); Makganye (2015); Posner (2015); Arowoiya (2014); Leichtfuss (2009); Thomas & Greenop (2008); Bham & Ross (2005); Biggs (2005)	Negative staff attitudes and behaviour e.g., impersonal care; poor support; poor communication; lack of cultural sensitivity, rudeness, and delayed assistance with patient's personal hygiene

For peer review only

Arowoiya (2014), Bertram (2013); Cawood (2012); Ntamo (2011); Kleineibst (2007)	Dissatisfaction with health care received
Kusambiza-Kiingi (2017); Mashau et (2016); Mudzi (2010); Kleineibst (2007); Rouilliard (2012); Felemengas (2004)	Lack of caregiver training

*No supporting factors reported.

Resources

Infrastructure

A mixed method study by Ntamo et al.(60) reported that substantial traveling distances were required to access rural healthcare facilities(60), this was echoed in Bryer's editorial on the need for community-based stroke care (42). Makganye et al.(57) reported that 71% of 85 rural patients (n = 60) lived over 25 km away from their nearest hospital (57). Physical access for people with a disability was further limited by poor building infrastructure (e.g. no ramps, vast distances between departments) or the surrounding uneven terrain (67).

Three records (longitudinal study, cross-sectional study and editorial) reported a lack of diagnostic equipment in rural facilities (23,35,42), in contrast with well-resourced urban rehabilitation centres (27,28) which often remained inaccessible (27,28,42) due to the location of these facilities requiring long traveling distances. A mixed methods study reported frequent stock-outs of basic medication at primary care level, which resulted in additional expenses and patients' reluctance to return to rural clinics (60) and these findings were echoed in an editorial (61).

Human Resources

Six studies reported high bed demand and rehabilitation workforce shortages led to high healthcare provider workloads (27,28,31,35,42,57). Therapists reportedly treated 2-3 times more patients than the daily recommendation (27). Mandizvidza (2017) (35) reported that nursing shortage at all healthcare levels in rural KwaZulu Natal impacted on basic stroke care. Better resourced urban tertiary hospitals in the Western Cape were also reported to experience staff shortages (35). A quantitative cross-sectional study reported that rehabilitation services are severely limited at primary care level with half of community health centers in the Western Cape providing rehabilitation services, and only two offering speech therapy (28). Stroke care was often provided by healthcare professionals without

For peer review only - http://bmjopen.bmj.com/site/about/guidelines.xhtml

specific stroke related training (27,30,35) (Table 3). No studies reported on the financial allocations or information systems in place to improve stroke service delivery.

Table 3. Resources related challenges (n = 16)

Author (year)	Resources
**Infrastructure**	
Mandizvidza (2017); Cawood (2012); Cunningham (2012); Rhoda et al (2009)	Lack of equipment (rural setting)
Ras (2009); Rhoda et al (2009)	Adequate equipment (urban rehabilitation centre setting)
Mandizvidza (2017); Biggs (2005)	Inadequate number of ambulances; in effective systems to request an ambulance
Maleka (2012); Ntamo (2011); Bryer (2009); Rhoda (2009)	Poor accessibility of health centres due to location, building structure or terrain surrounding the health facility
Matshikiza (2019); Mandizvidza (2017); De Villiers (2011); Bryer (2009); Ras (2009)	Insufficient number of beds or hospitals due to fiscal problems
Mandizvidza (2017); Villjoen (2016); Bryer (2009)	Inadequate special investigation infrastructure for diagnosis and management
Taylor & Ntusi (2019); Ntamo (2011)	Frequent medication outages
**Human Resources**	
Mandizvidza (2017); Makganye (2015); Cawood (2012); Bryer (2009), Ras (2009); Connor (2005)	Staff shortages
Mandizvidza (2017); Leichfust (2009); Ras (2009); Kleineibst (2007)	Lack of stroke specific training for staff

Context

Wellbeing and caregiver factors

Two longitudinal and one retrospective survey reported mental health problems such as anxiety and depression among PWS and caregivers (23,24,68). PWS also related feelings of confinement, personality changes, imposed family adjustments and caregiving burden (47,54,69). Gender-bias in caregiving roles were reported where women commonly left employment to assume caregiving responsibilities of male partners or parents (43) or children cared for women with stroke (44,54,67).

Financial implications

Financial burden increased when spouses became primary caregivers (without gainful employment) or through the employment of additional caregivers (54). Financial burden post

For peer review only - http://bmjopen.bmj.com/site/about/guidelines.xhtml

stroke was high due to additional caregiving costs (57,70) and limited access to disability-, old age- or child-support grants (49,62). Financial burden among rural stroke survivors was compounded by low income before stroke, difficulty in obtaining social grants due to limited awareness of eligibility criteria and the application processes, and lack of transport to submit grant applications (50,63). Poverty impacted access or utilization of rehabilitation as available finances were preferentially used to meet basic needs such as food (71).

Access to Transport

Six studies reported transport being a limiting factor to access care due to expensive private transport, unreliable public transport and inflated costs of a trip to accommodate assistive devices (29,34,36,60,62,72).

Cultural beliefs and Health Literacy

A systematic review and qualitative case study reported that PWS in SA held cultural beliefs regarding the cause and recovery of stroke, such as ascribing stroke to witchcraft or religious beliefs (6,33). Combined with poor health literacy (57,63,65) these beliefs further affected the care seeking ability of communities. Bham and Ross (2005) (33) reported that healthcare professionals needed greater awareness of cultural practices such as the inclusion of extended family in decision making procedures, adaption of communication style when interviewing older persons, and sensitivity to religious and traditional beliefs, to facilitate the inclusion and full participation of marginalised communities(33).

Community Engagement

Self-efficacy

Leichtfuss (2009) (30) found that PWS and/or their caregivers believed that they were not involved in care decision-making. Felemengas (2004) (54) and Cawood (2012) (31) reported that PWS were neither confident with self-health management nor satisfied with pre-discharge training and information (31,54). A large mixed methods study (62) that included a survey (N= 418) reported that PWS and caregivers lacked awareness of availability and benefit of rehabilitation services or support groups and this was echoed by Burton's editorial (73). Cawood et al (40) found that nearly half (n=53; 47%) of the participants in their cross sectional study indicated via a survey that they did not receive assistance from stroke organisations (37). Low participation in a peer support program was found (26) despite

For peer review only - http://bmjopen.bmj.com/site/about/guidelines.xhtml

patients who attended stroke support reporting better self-efficacy and feeling supported (31,62).

Community Integration

People with stroke were not fully re-integrated into their communities (58,74) due to negative attitudes of family, friends and society (31). Inaccessible community activities (28.3%), poor mental health (18.9%)(75), financial constraints (45.3%)(74), and inaccessible transport (62) contributed to limited community integration. Fear of stigmatisation (67), functional dependency especially due to incontinence (29,34,47,60,76), and fear of becoming a vulnerable victim of crime (37) also heightened limiting factors to integration.

Homecare resources

A Stroke Home Care booklet (in different languages) was developed for the SA context (77). In focus group discussions, seven stroke survivors (n=15; 46%) demonstrated improved knowledge, confidence, and ability to communicate information about their stroke after using the booklet (77). However, the sample included in this study was small and the booklet was only available in English when acceptability was tested, and the findings are therefore not generalizable. The stories and pictures were found to be culturally sensitive (77).

Reorganisation of Care

Educational and information resources

This review found two educational resources available via institutional websites for the public: Stroke Home Care booklet (77) and the SA contextualised Bridges Stroke Self-management intervention workbook (56). The MyStroke website (www.mystroke.co.za) was developed following a public health awareness campaign and lists available stroke care centres and services for better coordination (73). The mySOS app is an e-health initiative that directs and connects users with emergency care, potentially improving the timeliness of care. In rural settings telemedicine was used to connect with specialist services (78). However, none of these resources include trials of efficacy or determined the usage of the website or application.

Stroke Unit

At a central hospital in Western Cape, the stroke unit was associated with reduced mortality and increased rehabilitation referral, staff training and family involvement in treatment

For peer review only - http://bmjopen.bmj.com/site/about/guidelines.xhtml

decisions (45). Stroke units were recommended in evidence-based SA stroke guidelines care guidelines (79,80).

Palliative care integration

Findings based on focus groups of patients showed that palliative care should be incorporated into stroke care. However, better education of all stakeholders on palliative care benefits was needed (41).

Governance and Regulation

Two stroke clinical care guidelines for SA were identified (79,80). One focussed on acute and post-acute stroke care (80), and the other on stroke rehabilitation (79). Mandizvizda (2017) (35) evaluated the level of adherence to the acute stroke care guideline in all levels of care in the Western Cape province, and reported poor adherence in primary, secondary, and tertiary hospitals (general wards), with the two Stroke Units (situated in tertiary hospitals), being the most compliant (35). Challenges to adherence included staff shortages, limited access to diagnostic investigations and delays in patients presenting to healthcare facilities (35).

There were no national stroke specific policies. Whilst many people with disabilities are reliant on financial support from the government by means of grants, there was no specific policy on financial support for PWS or their caregivers. Poor intersectoral coordination between government departments was reported with regard to responsibilities for policies concerning persons with disabilities (81). Governance and Regulations was the most limited component with limited leadership and policy on how stroke care should be implemented and conducted at all levels of care.

Limiting and supporting factors

Health system limitations and factors that support the achievement of UHC for PWS in SA are presented in Figure 3. Findings of each health system component of the framework are mapped and identify challenges that speak to stroke care in the public sector.

Figure 3: Limiting and supporting factors toward achieving UHC

DISCUSSION

This scoping review summarises the available evidence of achieving health for all PWS in SA. The nature, extent, and distribution of data on SA stroke services in terms of the

For peer review only - http://bmjopen.bmj.com/site/about/guidelines.xhtml

individual framework components were found to be limited. Key health system limiting factors were a lack of governmental regulation in terms of stroke policies and guidelines; poor timeliness of care; a lack in continuity of care and a lack of a comprehensive MDT team at rural health facilities; bed and staff shortages and a lack of stroke-specific training; poor access to acute care and diagnostic equipment; regular medication outages; lack of caregiver training and contradictory reports on perceptions of care. Promising facilitating factors were adequately equipped and staffed urban tertiary facilities. Drivers to achieve UHC for PWS in SA may include better governance and regulation to mitigate fiscal shortfall resulting in infrastructure and human resource limiting factors, intersectoral collaboration between government departments to assist with access to social support and reliable, affordable transport to access healthcare.

A key finding of this review is a lack of adequate Governance and Regulations in terms of government support and investment in policy and treatment guidelines, resource distribution and commitment to evidence-based solutions (e.g. stroke units). Social equity for people with disabilities, including PWS, requires a renewed and concerted effort and commitment from the SA government to ensure that UHC for all is achieved (82). Administrative interventions by both government and hospital management are needed to address system-based limiting factors such as access to patients' medical records, obtaining appointments and physical access. Moreover, addressing staff shortages and improving stroke-specific training may mitigate excessive workload of healthcare workers and improve service delivery, as was achieved during a pilot program in Namibia, where an increase in the number of nurses resulted in improved service delivery (83). It is however important to note that the retention and attraction of health staff in rural and remote areas is multi-factorial (84,85). Therefore, the government needs to apply strategies which address the factors that impact on attraction and retention in the given context (84).

Established stroke units in hospitals have shown a decrease in mortality, increased MDT access and improved discharge planning compared to managing PWS in general medical wards (45). Limited governmental support may contribute to poor stroke outcomes, elevated levels of disability and low rates of community re-integration including return to work for PWS in SA. Greater accountability for financial and resource allocation to stroke services is required as the current limiting factors increase the overall societal economic burden, hinder national growth and development, as well as the achievement of universal stroke care.

For peer review only - http://bmjopen.bmj.com/site/about/guidelines.xhtml

Service Delivery and Context related factors were most frequently reported and were consistently reported as poor. South African health system weaknesses such as poor timeliness of care, a lack in continuity of care and a lack of a comprehensive MDT team are similar to health system weaknesses found in Rwanda and Malawi (86). The main hindrances affecting service delivery in SA were poor referral networks within and between healthcare facilities (26,29,35,36,40,71), inadequate caregiver training (23,36,47,54,69), fiscal inadequacies resulting in inadequate staffing levels at all levels of care (27,31,35,42,57,87), lack of stroke specific staff training (27,30,35,36), bed shortages (32,35,38,42), a lack of acute care and diagnostic equipment at primary and district health facilities (35,39,42), and a lack of availability of well-equipped rural rehabilitation facilities (28,31,35,44). As a result, many PWS are lost to follow-up care leading to poor management of comorbidities and potentially placing patients at risk of recurrence and secondary complications such as spasticity, pressure sores, aspiration pneumonia and mobility difficulties (88).

Access to equitable and affordable health care for PWS may be affected by contextual factors outside the healthcare system. Social determinants of health (poverty, education) and general safety are required to be addressed through intersectoral collaboration. Social service and health sector collaboration may ensure that eligible PWS are aware and have access to social grant support. This was echoed as an international need in a scoping review which included studies from North America, the United Kingdom, and Europe (89). Cooperation between both private and public transport services, and the health sector is needed to find a solution for accessible, affordable and reliable transport for PWS and their caregivers. Whilst there is robust empirical evidence of a lack of access to transport, especially in lower income communities, and the negative effects on health care, research on possible solutions and the effectiveness thereof is scarce (90,91).

Reported Supporting Factors

Despite the many limiting factors that were described, supporting factors to achieve UHC for PWS in SA were also reported. Supporting factors to UHC that is already in place include well-equipped rehabilitation facilities in urban areas (N = 2), perception of positive staff attitude (N = 10) and comprehensive MDT teams in urban, tertiary hospitals (N= 9). Although PWS reported their dissatisfaction with the care they have received (6,31,36,49,60) a number of studies reported patient and caregiver satisfaction, as well as positive staff attitudes, which were perceived to facilitate physical improvement through rehabilitation

For peer review only - http://bmjopen.bmj.com/site/about/guidelines.xhtml

compliance(25,26,29,31,37,41,57–59,61). This was consistent with findings where the attitude and emotional approach of health professionals as well as caregivers affected the level of motivation for rehabilitation attendance in PWS (92). Maclean et al.(92) found that a positive rapport between patients and healthcare providers resulted in increased motivation and easy transmission of information about rehabilitation (92).

Implications for future research

The limited supporting factors and multitude of limiting factors reported in the included studies highlight the gaps that remain and present opportunities for future research. Key questions include effect of continuity of care, timeliness of care and perceptions of care on the improvement of service delivery, as well as the effect of resource related factors such as fiscal management in terms of staff, bed allocation and access to diagnostic equipment as well as stroke related training.

Future research may focus on:

- Strategies to coordinate care for multi-morbidity (e.g. combined appointments with different health professionals) to minimise financial hardship on healthcare users and to evaluate effective and efficient holistic management of health, compared to silo treatment approaches.
- Extension of accessible, quality services beyond urban areas.
- Development and testing of stroke specific capacity development for staff that is evidence-based, patient-centered and holistic. Factors to highlight in training may include cultural responsiveness and awareness of the social determinants of health.
- Strategies to improve and implement person-centred discharge planning, which should include caregiver training and support before and after discharge.
- Development and evaluation of sustainable strategies to provide peer support groups either in person or on a digital platform, for both PWS and their caregivers, to provide ongoing support.
- Innovative public health campaigns via social media, television, or radio to increase the awareness of stroke signs and the urgency of seeking help. The impact, reach, and process evaluation of such campaigns should monitor effectiveness.

For peer review only - http://bmjopen.bmj.com/site/about/guidelines.xhtml

Strengths and limitations of the scoping review

We used a comprehensive search strategy that followed PRISMA guidelines, and robust methods that included double data extraction and review to produce an accurate, comprehensive state of the evidence. We used a people centred framework that acknowledged that health service provision should be coordinated around people's needs and preferences, and provided in a way that is safe, effective, timely, affordable, and of acceptable quality. The framework also acknowledged the social and economic determinants of health and political context. This review has several limitations. There was no limitation on study design for the inclusion of articles through methodological appraisal. This lack of rigour in the studies included may have led to non-generalisable conclusions. The variety in study design and aim made comparison of study results difficult.

CONCLUSION

Stroke is the leading cause of disability in adults in SA, which places strain on national social and healthcare services. Although the SA government has committed to the World Health Organisation (WHO) vision of achieving equitable, evidence-based rehabilitation for all by 2030, this review highlights the multifactorial nature of the health system in SA that requires strengthening and indicates the lack of readiness for UHC for PWS, especially pertaining to adequate governance and regulations.

Despite the available guidance on the best strategies to support healthcare systems in delivering stroke care services, the main findings of this review show that the stroke care services for PWS in SA are generally limited with a strong urban bias. Health system strengthening driven by good governance & regulation of health services, continuity of care, timeliness of care, accessibility to facilities, acute stroke care and diagnoses and well-equipped rehabilitation services is urgently needed. Health system limitations are compounded by contextual factors, highlighting the need for health system strengthening strategies that are tailored for local context.

The findings of this review have highlighted health systems challenges that speak to inequitable stroke care in the public sector, produced evidence that suggest that stroke care services are of sub-optimal quality, and highlighted evidence that stroke increases financial hardships for patients and their families, particularly for those who are of lower socio-economic status. The results of this review can be used to inform policymakers and

For peer review only - http://bmjopen.bmj.com/site/about/guidelines.xhtml

healthcare professionals of healthcare system challenges and opportunities to effectively move towards UHC for PWS in SA. Governments should be held more accountable for stroke care in terms of financial resource allocation and prioritize and include this marginalised group in the proposed national health insurance scheme.

DECLARATIONS

Availability of data and materials All data generated or analysed during this study will be included in the published scoping review article.

Ethics and dissemination Ethical approval was not required for this scoping review, as it only included published and publicly available data. The findings of this review is published in an open-access, peer-reviewed journal and developed an accessible summary of the results for website posting and stakeholder meetings.

Contributors SvN and SK in consultation with all authors constructed the search. SvN, SK and MC extracted all data in consultation with all authors. SvN, SK, GI-J, JW, QAL and TS analysed the extracted data. SvN drafted and revised the paper. SK, GI-J, MC, SF, RE, JW, QAL and TS reviewed the manuscript and provided feedback on the drafts. All authors read and approved the final manuscript.

Funding This research was commissioned by the National Institute for Health Research (NIHR) Global Health Policy and Systems Research Development Award using UK aid from the UK government. Grant number NIHR130180.

Disclaimer The views expressed in this publication are those of the author(s) and not necessarily those of the NIHR or the Department of Health and Social Care.

Competing interests None declared.

Patient consent for publication Not required.

For peer review only

REFERENCES

1. Naghavi M, Abajobir AA, Abbafati C, et al. Global, regional, and national age-sex specifc mortality for 264 causes of death, 1980-2016: A systematic analysis for the Global Burden of Disease Study 2016. Lancet. 2017;

2. Scheffler E, Mash R. Surviving a stroke in South Africa: outcomes of home-based care in a low-resource rural setting. Top Stroke Rehabil. 2019 Aug 18;26(6):423–34.

3. Pillay-van Wyk V, Msemburi W, Laubscher R, et al. Mortality trends and differentials in South Africa from 1997 to 2012: second National Burden of Disease Study. Lancet Glob Heal. 2016;

4. Maredza M, Bertram MY, Gómez-Olivé XF, et al. Burden of stroke attributable to selected lifestyle risk factors in rural South Africa. BMC Public Health [Internet]. 2016 Jan 1;16:143. Available from: http://search.ebscohost.com/login.aspx?direct=true&db=awn&AN=26869067&site=ehost-live&scope=site

5. Coovadia H, Jewkes R, Barron P, et al. The health and health system of South Africa: historical roots of current public health challenges. The Lancet. 2009.

6. Bertram MY, Katzenellenbogen J, Vos T, et al. The disability adjusted life years due to stroke in South Africa in 2008 [Internet]. Vol. 8 Suppl A1, International journal of stroke: official journal of the International Stroke Society. 2013. p. 76–80. Available from: http://search.ebscohost.com/login.aspx?direct=true&db=awn&AN=23295022&site=ehost-live&scope=site

7. Maredza M, Bertram MY, Tollman SM. Disease burden of stroke in rural South Africa: An estimate of incidence, mortality and disability adjusted life years. BMC Neurol. 2015 Apr 12;15(1).

8. Eksteen G, Mungal-Singh V. Salt intake in South Africa: A current perspective. Journal of Endocrinology, Metabolism and Diabetes of South Africa. 2015.

9. Kabudula CW, Houle B, Collinson MA, et al. Progression of the epidemiological transition in a rural South African setting: findings from population surveillance in Agincourt, 1993-2013. BMC Public Health. 2017 May 10;17(1).

10. WHO, The World Bank. Tracking Universal Health Coverage: 2017 Global Monitoring Report. World Health Organisation. 2017.

11. Vallabhjee K D V. Western Cape Government. Healthcare 2030: the road to wellness. Cape Town: Western Cape Department of Health. 2014.

12. Kuper H, Hanefeld J. Debate: can we achieve universal health coverage without a focus on disability? BMC Health Serv Res. 2018;

13. Hashemi G, Kuper H, Wickenden M. SDGs, Inclusive Health and the path to Universal Health Coverage. Disabil Glob South. 2017;

14. Evans DB, Etienne C. Health systems financing and the path to universal coverage. Bulletin of the World Health Organization. 2010.

15. Louw Q, Twizeyemariya A, Grimmer K, et al. Estimating the costs and benefits of stroke rehabilitation in South Africa. J Eval Clin Pract. 2020 Aug 1;26(4):1181–7.

16. Levac D, Colquhoun H, O'Brien KK. Scoping studies: Advancing the methodology. Implement Sci. 2010 Sep 20;5(1).

17. Van Niekerk SM, Inglis-Jassiem G, Kamalakannan S, et al. Achieving universal health

For peer review only

For peer review only - http://bmjopen.bmj.com/site/about/guidelines.xhtml

coverage for people with stroke in South Africa: protocol for a scoping review. BMJ Open. 2020 Oct;10(10):e041221.

18. Tricco AC, Lillie E, Zarin W, et al. PRISMA extension for scoping reviews (PRISMA-ScR): Checklist and explanation. Annals of Internal Medicine. 2018.

19. Olmen J Van, Criel B, Bhojani U, et al. The Health System Dynamics Framework: The introduction of an analytical model for health system analysis and its application to two case-studies. Heal Cult Soc. 2012;2(1):1–21.

20. World Health Organization (WHO). Framework on integrated, people-centred health services: Report by the Secretariat. World Heal Assem. 2016;

21. Ritchie J, Lewis J, Nicholls C, et al. Qualitative research practice: A guide for social science students and researchers [Internet]. 2013 [cited 2021 Jan 10]. Available from: https://books.google.co.za/books?hl=en&lr=&id=EQSIAwAAQBAJ&oi=fnd&pg=PP1&dq=Ritchie+J,+Lewis+J,+Nicholls+C,+Ormston+R&ots=l-QMhvTv3P&sig=Hz7vuHbw4WR5vGMtiWPszn_KSy4

22. Wasserman D. Community-based care of stroke patients in a rural African setting. South African Med J [Internet]. 2009 Jan 1;99(8):13. Available from: http://search.ebscohost.com/login.aspx?direct=true&db=awn&AN=803237&site=ehost-live&scope=site

23. Rouillard S, De Weerdt W, De Wit L, et al. Functioning at 6 months post stroke following discharge from inpatient rehabilitation. South African Med J. 2012;102(6):545–8.

24. Rhoda A, Cunningham N, Azaria S, et al. Provision of inpatient rehabilitation and challenges experienced with participation post discharge: quantitative and qualitative inquiry of African stroke patients. BMC Health Serv Res [Internet]. 2015 Jan 1;15:423. Available from: http://search.ebscohost.com/login.aspx?direct=true&db=awn&AN=26412081&site=ehost-live&scope=site

25. Groenewald R, Rhoda AJ. Multidisciplinary rehabilitation outcomes of stroke patients in the Western Cape of South Africa [Internet]. Vol. 23, African Journal for Physical, Health Education, Recreation and Dance. 2017. p. 267–76. Available from: http://search.ebscohost.com/login.aspx?direct=true&db=awn&AN=ajpherd-164230&site=ehost-live&scope=site

26. Joseph C. Determining the process of rehabilitation and the outcomes of patients at a specialised in-patient centre in the Western Cape. 2011;2–216. Available from: http://etd.uwc.ac.za/xmlui/handle/11394/2973

27. Ras T. An audit of geriatric stroke rehabilitation services at a post-acute hospital (Booth Memorial) in urban Cape Town, South Africa. Diss Univ Cape T. 2009;

28. Rhoda A, Mpofu R, DeWeerdt W. The rehabilitation of stroke patients at community health centres in the Western Cape. South African J Physiother. 2009;65(3).

29. De la Cornillère W. Participants' experience of the Bishop Lavis Rehabilitation Centre stroke group [Internet]. 2007. Available from: http://search.ebscohost.com/login.aspx?direct=true&db=awn&AN=NX0185175&site=ehost-live&scope=site

30. Leichtfuss U. Aspects of stroke rehabilitation in private acute-care hospitals. Master Philos (mphil) Fac Heal Sci. 2009;

31. Cawood J. Rehabilitation outcomes of uninsured stroke survivors in the Helderberg Basin [Internet]. 2012. Available from: http://search.ebscohost.com/login.aspx?direct=true&db=awn&AN=NX0217654&site=ehost-

For peer review only - http://bmjopen.bmj.com/site/about/guidelines.xhtml

live&scope=site

32. De Villiers L, Badri M, Ferreira M, et al. Stroke outcomes in a socio-economically disadvantaged urban community. S Afr Med J. 2011 May;101(5):345–8.

33. Bham Z, Ross E. Traditional and western medicine: cultural beliefs and practices of South African Indian Muslims with regard to stroke [Internet]. Vol. 15, Ethnicity & Disease. 2005. p. 548–54. Available from: http://search.ebscohost.com/login.aspx?direct=true&db=awn&AN=16259475&site=ehost-live&scope=site

34. Cawood J, Visagie S. Stroke management and functional outcomes of stroke survivors in an urban Western Cape Province setting. South African J Occup Ther. 2016;46(3):21–6.

35. Mandizvidza V. Quality of current ischaemic stroke care practices in the Cape Metro Health District, South Africa. Diss Univ Cape T. 2017;

36. Kleineibst LJ. The effectiveness of a caregiver support programme to address the needs of primary caregivers of stroke patients in a low socio-economic community [Internet]. 2007. Available from: http://search.ebscohost.com/login.aspx?direct=true&db=awn&AN=NX0185273&site=ehost-live&scope=site

37. Cawood J, Visagie S. Environmental factors influencing participation of stroke survivors in a Western Cape setting. African J Disabil. 2015;4(1):1–9.

38. Matshikiza. Barriers to acute stroke care at a tertiary Hospital in the Western Cape. 2019;

39. Viljoen C. Audit of the quality and cost of acute inpatient stroke care in the general medical wards at Groote Schuur Hospital. 2014;

40. Cawood J, Visagie S, Mji G. Impact of post-stroke impairments on activities and participation as experienced by stroke survivors in a Western Cape setting. South African J Occup Ther. 2016;46(2):10–5.

41. Hossain M. To investigate the Need for Palliative care in Cerebrovascular Accident (stroke) patients at Ladysmith Regional Hospital. 2014;

42. Bryer A. The need for a community-based model for stroke care in South Africa. South African Med J [Internet]. 2009 Jan 1;99(8):10. Available from: http://search.ebscohost.com/login.aspx?direct=true&db=awn&AN=803234-1&site=ehost-live&scope=site

43. Hilton J. Factors that influence the quality of life of a caregiver caring for a patient with stroke. 2011;

44. Cunningham NL. The profile and outcomes of stroke patients discharged from a hospital in the Eastern Cape [Internet]. 2012. Available from: http://search.ebscohost.com/login.aspx?direct=true&db=awn&AN=NX0215155&site=ehost-live&scope=site

45. De Villiers L, Kalula SZ, Burch VC. Does multidisciplinary stroke care improve outcome in a secondary-level hospital in South Africa? [Internet]. Vol. 4, International journal of stroke: official journal of the International Stroke Society. 2009. p. 89–93. Available from: http://search.ebscohost.com/login.aspx?direct=true&db=awn&AN=19383048&site=ehost-live&scope=site

46. Rhoda A, Smith M, Putman K, et al. Motor and functional recovery after stroke: a comparison between rehabilitation settings in a developed versus a developing country [Internet]. Vol. 14, BMC Health Services Research. 2014. p. 82. Available from:

For peer review only - http://bmjopen.bmj.com/site/about/guidelines.xhtml

http://search.ebscohost.com/login.aspx?direct=true&db=awn&AN=24559193&site=ehost-live&scope=site

47. Mudzi W. Impact of caregiver education on stroke survivors and their caregivers. Dissertation. 2010;

48. Parekh I, Rhoda A. Functional outcomes of stroke patients admitted to a tertiary hospital in the Western Cape, South Africa [Internet]. Vol. 69, South African Journal of Physiotherapy. 2013. p. 14. Available from: http://search.ebscohost.com/login.aspx?direct=true&db=awn&AN=722250&site=ehost-live&scope=site

49. Arowoiya A. Participation restrictions of stroke patients living in the community at selected community health centres in the metropole districts in the Western Cape, South Africa. Diss Univ West Cape. 2014;

50. Mudzi W, Stewart A, Musenge E. Community participation of patients 12 months post-stroke in Johannesburg, South Africa. African J Prim Heal Care Fam Med. 2013;5(1):1–9.

51. Parekh IS. Factors influencing functional outcome of stroke patients admitted to a tertiary hospital [Internet]. 2012. Available from: http://search.ebscohost.com/login.aspx?direct=true&db=awn&AN=NX0214919&site=ehost-live&scope=site

52. Rhoda A, Mpofu R, De Weerdt W. Activity limitations of patients with stroke attending out-patient facilities in the Western Cape, South Africa [Internet]. Vol. 67, South African Journal of Physiotherapy. 2011. p. 16–22. Available from: http://search.ebscohost.com/login.aspx?direct=true&db=awn&AN=702551&site=ehost-live&scope=site

53. Blackwell Z, Littlejohns P. A review of the management of dysphagia: a South African perspective [Internet]. Vol. 42, Journal of Neuroscience Nursing. 2010. p. 61–70. Available from: http://search.ebscohost.com/login.aspx?direct=true&db=awn&AN=20422791&site=ehost-live&scope=site

54. Felemengas M. Caregiver experiences and perceptione of the effects of stroke in the family [Internet]. 2004. Available from: http://search.ebscohost.com/login.aspx?direct=true&db=awn&AN=NX0150149&site=ehost-live&scope=site

55. Mabunda SA. An Evaluation of the Role of an Intermediate Care Facility in the Continuum of Care in Western Cape, South Africa. Dissertation [Internet]. 2015;53(July):227. Available from: http://library.wur.nl/WebQuery/wurpubs/fulltext/353506

56. Groenewald RC. The Adaptation and Contextualization of the Bridges Stroke Self-Management Intervention for Patients Living With Stroke in the Western Cape, South Africa. 2018;(August). Available from: http://etd.uwc.ac.za/ii

57. Makganye TM. The experience of patients and caregivers following a stroke. 2015;(December).

58. Kotsokoane FM, Tshabalala MD, Nukeri AL, et al. The level of integration of stroke survivors receiving rehabilitation services in Soshanguve clinics, South Africa [Internet]. Vol. 24, African Journal for Physical, Health Education, Recreation and Dance. 2018. p. 564–78. Available from: http://search.ebscohost.com/login.aspx?direct=true&db=awn&AN=ajpherd-181896&site=ehost-live&scope=site

59. Kusambiza-Kiingi A. Community reintegration and satisfaction of survivors of stroke

For peer review only - http://bmjopen.bmj.com/site/about/guidelines.xhtml

receiving physiotherapy services in the community health centres within the Johannesburg area. 2016;1–2.

60. Ntamo PN. Poor attendance of physiotherapy treatment by stroke out-patients in Mthatha General Hospital [Internet]. 2011. Available from: http://search.ebscohost.com/login.aspx?direct=true&db=awn&AN=NX0207219&site=ehost-live&scope=site

61. Taylor A, Ntusi NAB. Guest Editorial: Evolving concepts of stroke and stroke management in South Africa: Quo vadis? [Internet]. Vol. 109, South African Medical Journal. 2019. p. 69–71. Available from: http://search.ebscohost.com/login.aspx?direct=true&db=awn&AN=samj-184307&site=ehost-live&scope=site

62. Biggs D. Health Promotion Needs of Stroke Patients Accessing Community Health Centres in the Metropole Region of the Western Cape Health Promotion Needs of Stroke Patients Accessing Community Health Centres in the. 2005;(November).

63. Thomas M, Greenop K. Caregiver Experiences and Perceptions of Stroke. Heal SA Gesondheid [Internet]. 2008 Jan 1;13(1):29–40. Available from: http://search.ebscohost.com/login.aspx?direct=true&db=awn&AN=IM-009014&site=ehost-live&scope=site

64. Posner G. the Experiences of Employed Caregivers, Working in Private South African Homes, With Patients Who Have Suffered From a Stroke. 2015; Available from: http://wiredspace.wits.ac.za/jspui/bitstream/10539/19598/2/MASTERS DISSERTATION G POSNER.pdf

65. Smith JL. Self-management strategies employed by stroke survivors in the Western Cape, South Africa. Diss Univ Cape T. 2019;

66. Mashau NS, Netshandama VO, Mudau MJ. Self-reported impact of caregiving on voluntary home-based caregivers in Mutale Municipality, South Africa. African J Prim Heal Care Fam Med. 2016;8(2):1–5.

67. Maleka M, Stewart AS, Hale L. The experience of living with stroke in low urban and rural socioeconomic areas of South Africa [Internet]. South African Journal of Physiotherapy. 2012. p. 5–29. Available from: http://search.ebscohost.com/login.aspx?direct=true&db=awn&AN=705374&site=ehost-live&scope=site

68. Rhoda AJ. Health-related quality of life of patients six months poststroke living in the Western Cape, South Africa. African J Disabil. 2014;3(1):1–6.

69. Kusambiza-Kiingi A, Maleka D, Ntsiea V. Stroke survivors' levels of community reintegration, quality of life, satisfaction with the physiotherapy services and the level of caregiver strain at community health centres within the Johannesburg area. African J Disabil. 2017;6:1–8.

70. Hassan S, Visagie S, Mji G. Strain experienced by caregivers of stroke survivors in the Western Cape [Internet]. Vol. 67, South African Journal of Physiotherapy. 2011. p. 4–8. Available from: http://search.ebscohost.com/login.aspx?direct=true&db=awn&AN=702553&site=ehost-live&scope=site

71. Masuku KP, Mophosho M, Tshabalala M. "I felt pain. Deep pain...": Experiences of primary caregivers of stroke survivors with aphasia in a South African township. African J Disabil. 2018;7:1–7.

72. Biggs D, Rhoda A. Health risk behaviours of stroke patients in the Western Cape, South

For peer review only

Africa. South African J Physiother [Internet]. 2008 Jan 1;64(1):38–42. Available from: http://search.ebscohost.com/login.aspx?direct=true&db=awn&AN=577099&site=ehost-live&scope=site

73. Burton A. South Africa: stroke units out of the blue [Internet]. Vol. 15, The Lancet. Neurology. 2016. p. 359–60. Available from: http://search.ebscohost.com/login.aspx?direct=true&db=awn&AN=26971657&site=ehost-live&scope=site

74. Elloker T. Social support and participation restrictions in patients living with stroke in the Western Cape, SOuth Africa. Dissertation. 2015;

75. Cunningham N, Rhoda A. Outcomes of stroke patients discharged from an in-patient facility in the Eastern Cape, South Africa: a mixed methods design [Internet]. Vol. 70, South African Journal of Physiotherapy. 2014. p. 26–31. Available from: http://search.ebscohost.com/login.aspx?direct=true&db=awn&AN=722129&site=ehost-live&scope=site

76. Mamabolo V. The Influence of Demographic , Environmental and Physical Factors on Functional Independence Post Stroke Correspondence to : 2008;(011):19–22.

77. Botha JH. The refinement of a booklet on stroke care at home. 2008;

78. Faux S. Managing stroke survivors int he community. Mod Med South Africa [Internet]. 2006 Jan 1;31(1 January):8–14. Available from: http://search.ebscohost.com/login.aspx?direct=true&db=awn&AN=D-18283&site=ehost-live&scope=site

79. South African Contextualised stroke rehabilitation guideline (SACSRG), 2019. 2019;

80. Bryer A, Connor M, Haug P, et al. South African guideline for management of ischaemic stroke and transient ischaemic attack 2010: a guideline from the South African Stroke Society (SASS) and the SASS Writing Committee [Internet]. Vol. 100, S A M J South African Medical Journal. 2010. p. 747–78. Available from: http://search.ebscohost.com/login.aspx?direct=true&db=awn&AN=21081029&site=ehost-live&scope=site

81. Hussey M, Maclachlan M, Mji G. Barriers to the Implementation of the Health and Rehabilitation Articles of the United Nations Convention on the Rights of Persons with Disabilities in South Africa. Kerman Univ Med Sci [Internet]. 2017;6(4):207–218. Available from: http://dx.doi.org/10.15171/ijhpm.2016.117

82. National Department of Health. Strengthening the South African health system: Towards an integrated and unified health system. Pres Heal Summit 2018. 2018;

83. Vindigni SM, Riley PL, Kimani F, et al. Kenya's emergency-hire nursing programme: A pilot evaluation of health service delivery in two districts. Hum Resour Health. 2014;

84. Lehmann U, Dieleman M, Martineau T. Staffing remote rural areas in middle- and low-income countries: A literature review of attraction and retention. BMC Health Serv Res. 2008;

85. Hatcher AM, Onah M, Kornik S, et al. Placement, support, and retention of health professionals: National, cross-sectional findings from medical and dental community service officers in South Africa. Hum Resour Health. 2014;

86. Chimatiro GL, Rhoda AJ. Scoping review of acute stroke care management and rehabilitation in low and middle-income countries. BMC Health Serv Res. 2019 Nov;19(1):789.

87. Connor M. Stroke management in South Africa - who is responsible?: guest editorial [Internet]. Vol. 8, South African Psychiatry Review. 2005. p. 125–6. Available from:

For peer review only - http://bmjopen.bmj.com/site/about/guidelines.xhtml

http://search.ebscohost.com/login.aspx?direct=true&db=awn&AN=381538&site=ehost-live&scope=site

88. Bates B, Choi JY, Duncan PW, et al. Veterans Affairs/Department of Defense Clinical Practice Guideline for the Management of Adult Stroke Rehabilitation Care: Executive summary. Stroke. 2005.

89. Cameron JI, Tsoi C, Marsella A. Optimizing stroke systems of care by enhancing transitions across care environments. Stroke. 2008;

90. Bambra C, Gibson M, Sowden A, et al. Tackling the wider social determinants of health and health inequalities: Evidence from systematic reviews. Journal of Epidemiology and Community Health. 2010.

91. Syed ST, Gerber BS, Sharp LK. Traveling towards disease: Transportation barriers to health care access. Journal of Community Health. 2013.

92. Maclean N, Pound P, Wolfe C, et al. Qualitative analysis of stroke patients' motivation for rehabilitation. Br Med J. 2000;

LEGEND

Figure 1: Framework components

Figure 2: PRIMSA flow chart

Figure 3: Limiting and supporting factors toward achieving UHC

Supplementary File

S1: Framework component definitions

Supplementary File S2: Basic demographic information of included records (N=60)

Supplementary file S3: Components assessed in included studies

Supplementary file S4: Definitions

For peer review only - http://bmjopen.bmj.com/site/about/guidelines.xhtml

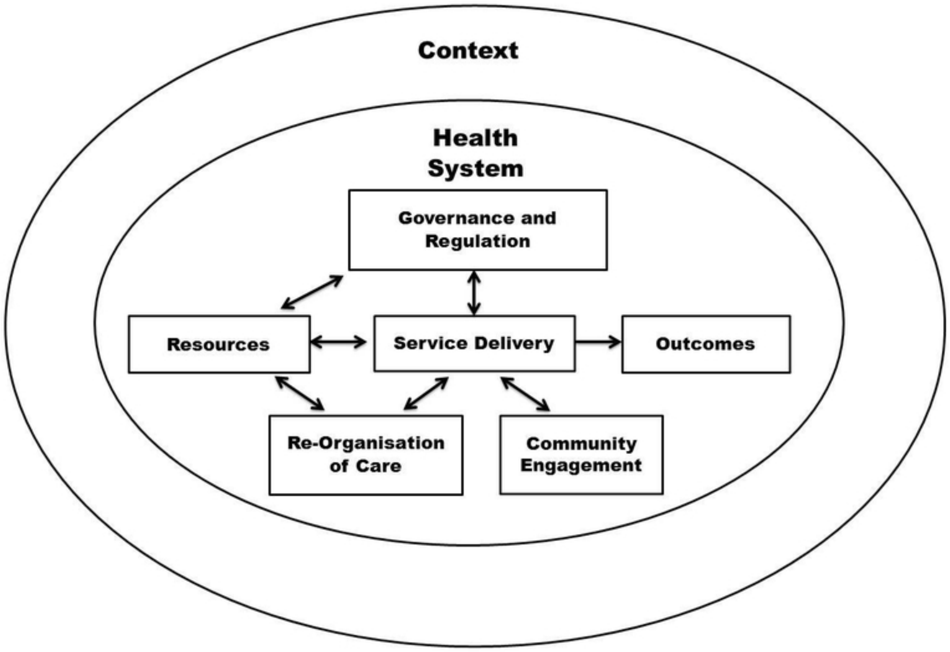

Figure 1: Framework components

68x51mm (300 x 300 DPI)

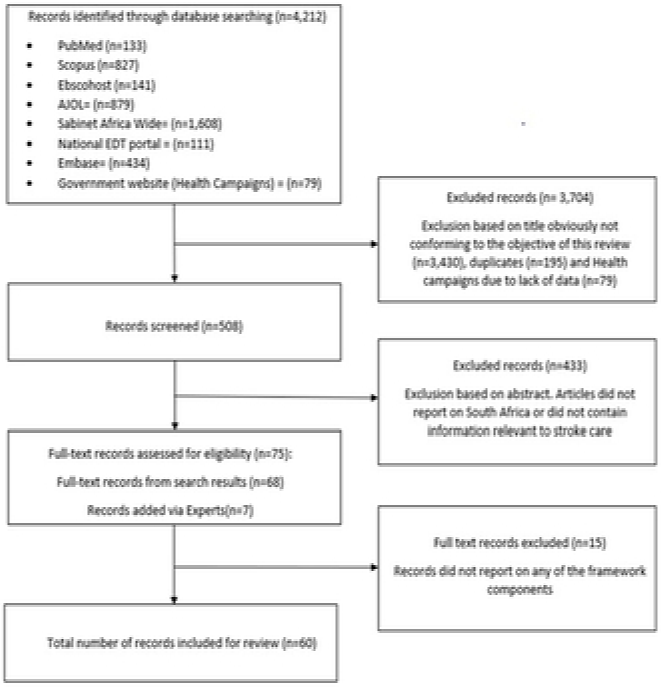

Figure 2: PRISMA flow chart

33x36mm (300 x 300 DPI)

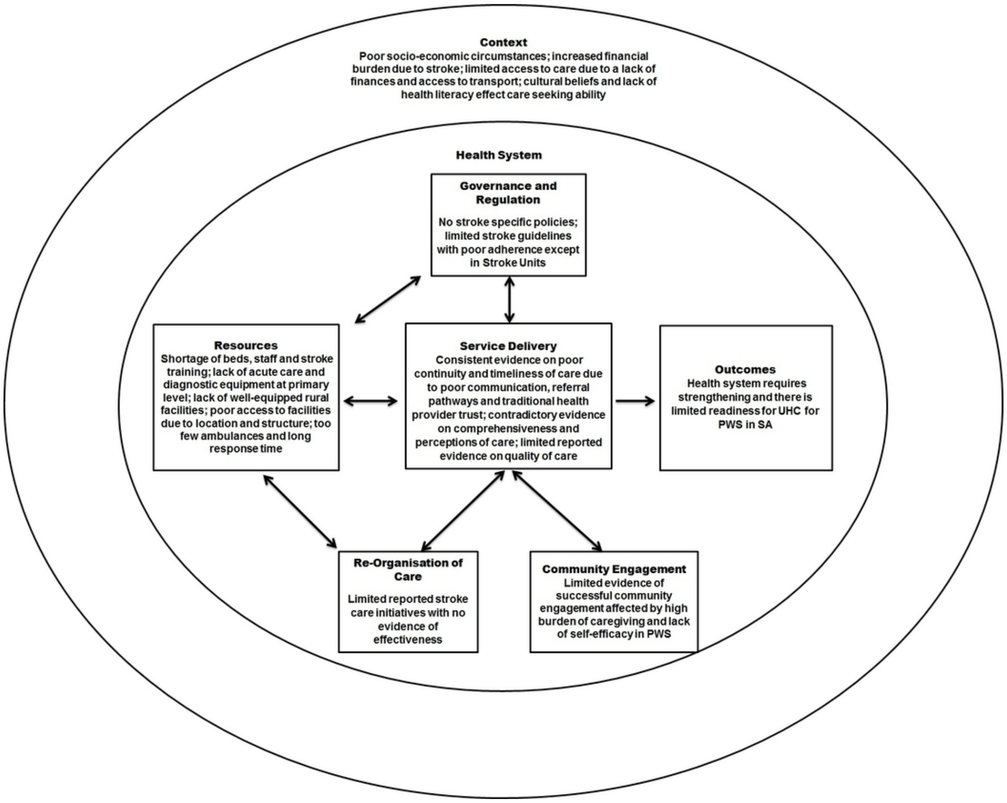

Figure 3: Limiting and supporting factors toward achieving UHC

88x70mm (300 x 300 DPI)

Supplementary file S1

FRAMEWORK COMPONENT	DESCRIPTION (data items)
**Governance/Regulation**	**Description:** a wide range of steering and rulemaking-related functions carried out by governments/decisions makers as they seek to achieve national and/or provincial health policy objectives that are conducive to UHC<br>**Data Items:** Healthcare policies at national or provincial levels; resource allocation policies; accountability monitoring; coordination and regulations; clinical treatment guidelines.
**Resources**	**Description:** All resources specific to the health care facility – including the physical structure and resources enabling or hindering delivery of health services.<br>**Data Items:**<br>**Infrastructure:** Accessibility of the health care facilities; maintenance of infrastructure; availability of equipment/testing facilities (e.g. CT Scans)<br>**Human resources:** Availability; health workforce distribution – health professions/experience or specialisation/gender; role definitions; undergraduate & continuous training; workload; patient vs therapist ratio.<br>**Financial resources:** Finance allocation and affordability; funding sources; healthcare packages; salaries/fair wages.; sustainability.
**Service Delivery**	**Description:** Delivery of different health services as well as user experience.<br>**Data Items:** Level of care; comprehensiveness; quality and/or perceptions of care; multi-professional health teams; continuity of care, timeliness of care; health services and service providers (private/public; for-profit or not–for-profit, formal or informal, professional or non-professional, allopathic or traditional, remunerated or voluntary).
**Context**	**Description:** All contextual factors influencing the patient/community access of the health care system.<br>**Data Items:** Social determinants of health: socio-economic, education, health literacy, technological, cultural, political and environmental environments.
**Re-orientation of care**	**Description:** New and innovate health care solutions to improve coordination of health services and continuous health care; and intersectoral coordination.<br>**Data Items:** New technologies and strategies (eHealth; shared electronic medical records; telemedicine; m-health)
**Community engagement**	**Description:** Engaging and empowering individuals, families, communities and informal caregivers to facilitate common decision-making and self-efficacy. Reaching underserved and marginalised communities.

Application of the key characteristics of the analytical framework

For peer review only

FRAMEWORK COMPONENT	DESCRIPTION (data items)
**Governance/Regulation**	**Description:** a wide range of steering and rulemaking-related functions carried out by governments/decisions makers as they seek to achieve national and/or provincial health policy objectives that are conducive to UHC<br>**Data Items:** Healthcare policies at national or provincial levels; resource allocation policies; accountability monitoring; coordination and regulations; clinical treatment guidelines.
**Resources**	**Description:** All resources specific to the health care facility – including the physical structure and resources enabling or hindering delivery of health services.<br>**Data Items:**<br>**Infrastructure:** Accessibility of the health care facilities; maintenance of infrastructure; availability of equipment/testing facilities (e.g. CT Scans)<br>**Human resources:** Availability; health workforce distribution – health professions/experience or specialisation/gender; role definitions; undergraduate & continuous training; workload; patient vs therapist ratio.<br>**Financial resources:** Finance allocation and affordability; funding sources; healthcare packages; salaries/fair wages.; sustainability.
**Service Delivery**	**Description:** Delivery of different health services as well as user experience.<br>**Data Items:** Level of care; comprehensiveness; quality and/or perceptions of care; multiprofessional health teams; referral systems; service delivery models; health services and service providers (private/public; for-profit or not–for-profit, formal or informal, professional or non-professional, allopathic or traditional, remunerated or voluntary).
**Context**	**Description:** All contextual factors influencing the patient/community access of the health care system.<br>**Data Items:** Social determinants of health: socio-economic, education, health literacy, technological, cultural, political and environmental environments.
**Re-orientation of care**	**Description:** New and innovate health care solutions to improve coordination of health services and continuous health care; and intersectoral coordination.<br>**Data Items:** New technologies and strategies (eHealth; shared electronic medical records; telemedicine; m-health)
**Community engagement**	**Description:** Engaging and empowering individuals, families, communities and informal caregivers to facilitate common decision-making and self-efficacy. Reaching underserved and marginalised communities.

For peer review only

Supplementary file S2. Detailed demographic information of all included records (N=60)

S.no	Author (year)	Province	Area	Level of care	Literature	Aim	Study design	Sample characteristics
1	Arowoiya (2014)	Western Cape	Urban	Primary Healthcare (CHC)	Dissertations	To determine and explore the participation restrictions experienced by stroke patients	Mixed Methods (Survey + FGDs)	120 stroke patients receiving Physiotherapy for survey & 2 FGDs with 17 stroke patients
2	Bertram et al (2013)	South Africa (Pan)	Undefined	Undefined	Primary Literature (peer reviewed publications)	To synthesize the data surrounding stroke in South Africa and calculate disability adjusted life years attributable to stroke in South Africa in 2008	Systematic review	Prevalence and mortality related studies on stroke and vital registration data
3	Bham & Ross 2005	Not reported	Undefined	Community	Primary Literature (peer reviewed publications)	To investigate the beliefs of caregivers and traditional healers within the South African Indian Muslim community regarding the etiology and treatment of stroke and the persons likely to be consulted in this regard	Descriptive case study design (Qualitative)	10 SAIM caregivers of people who had sustained strokes, as well as 10 SAIM traditional healers, who had treated stroke patients.
4	Biggs (2005)	Western Cape	Urban	Primary Healthcare (CHC)	Dissertations	To determine the health promotion needs of stroke patients accessing selected Community Health Centres in the Metropole region of the	Mixed Methods (Survey + In-depth interview)	418 stroke patients, representing each of the health districts of the Metropole region of the Western Cape for the survey and

						Western Cape.		12 stroke patients for Interviews
5	Biggs & Rhoda 2008	Western Cape	Urban	Primary Healthcare (CHC)	Primary Literature (peer reviewed publications)	To determine the health risk behaviours and factors that influence these behaviours of stroke patients in the Metropole Region of the Western Cape, South Africa	Mixed Methods (Survey + In-depth interview)	417 stroke patients – survey<br>12 stroke patients for Interviews
6	Blackwell & Littlejohns 2010	Gauteng,	Undefined	Undefined	Primary Literature (peer reviewed publications)	To measure the prevalence and review the assessment and management strategies related to dysphagia in three private rehabilitation clinics in South Africa	Review of records and thematic analysis	30 stroke patient records from three private rehabilitation clinics – total 90 records
7	Botha (2008)	Western Cape	Undefined	Community	Dissertations	To refine and pilot a training booklet for caregivers of stroke survivors for further implementation	Mixed methods (Literature review + Checklist development + survey + FGDs)	Sample – 1: 11 Stroke patients in WCRC<br>Sample – 2: 1Family members /other carers of Stroke patients in WCRC<br>Sample – 3: 4 Home based carers and 22 nursing assistants attending carer training at WCRC<br>Sample – 4: 4 Stroke patients and their 4 caregivers participating in home-based care programme at university of

								Western Cape in Nyanga<br>Sample -5: Stroke patients in WCRC<br>Total 15 stroke patients and 31 caregivers
8	Bryer (2009)	South Africa	Undefined	Undefined	Primary Literature (peer reviewed publications)	There is an urgent need to develop a model of community-based stroke care with appropriate Rehabilitation facilities and trained professionals<br>In South Africa, particularly in under-resourced areas	Editorial	NA
9	Bryer et al (2010)	South Africa	NA	NA	Primary Literature (peer reviewed publications, Clinical Guideline)	The objective was to update the guideline published in 2000, to place the recommendations within the current South African context, and to grade evidence according to the level of scientific rigour for management of ischaemic stroke and transient ischaemic attack 2010	SASS writing committee Guidelines	NA
10	Burton 2016	South Africa	Undefined	Undefined	Primary Literature (peer reviewed publications)	To investigate the efforts of a woman with	Editorial	NA

For peer review only

						a talent for getting things done for bringing stroke units out of the blue for South Africa		
11	Cawood 2012	Western Cape	Urban	Community	Dissertations	To determine if uninsured stroke survivors living in the Helderberg Basin (Western Cape) reached their optimal rehabilitation outcome levels and if not, what environmental barriers contributed to this.	A descriptive, mixed methods study (Surev + Interview)	53 stroke survivors (quantitative)<br>5 Stroke survivors (qualitative)
12	Cawood& Visagie (2015)	Western Cape	Urban	Community	Primary Literature (peer reviewed publications)	To determine environmental barriers and facilitators to participation experienced by a group of stroke survivors in the Western Cape province of South Africa.	A descriptive, mixed methods study (Surev + Interview)	53 stroke survivors (quantitative)<br>5 Stroke survivors (qualitative)
13	Cawood & Visagie (2016)	Western Cape	Urban	Community	Primary Literature (peer reviewed publications)	To describe the functional outcomes achieved by stroke survivors in an urban Western Cape Province setting to add to the information on stroke management	A descriptive, mixed methods study (Surev + Interview)	53 stroke survivors (quantitative)<br>5 Stroke survivors (qualitative)
14	Cawood et	Western	Urban	Community	Primary Literature (peer	To explore causal	Cross-sectional	53 stroke survivors

	al (2016)	Cape			reviewed publications)	connections between impairments, activity limitations and participation restrictions after stroke.	Study	
15	Connor (2005)	Sub Saharan Africa	Undefined	Undefined	Primary Literature (peer reviewed publications)	To understand the burden of stroke in black populations in sub-Saharan Africa	Systematic Review	All articles relevant to stroke in Sub Saharan Africa
16	Cunningham (2012)	Eastern cape	Urban	Uitenhage Provincial Hospital	Dissertations	To determine and explore the outcomes of stroke patients admitted to Uitenhage provincial hospital	Mixed Methods (quantitative survey + secondary data analysis + Semi-structured interviews)	168 stroke patient records for secondary data analysis, 24 stroke patients for prospective survey and 9 stroke patients for the qualitative study
17	Cunningham & Rhoda 2014	Eastern Cape	Urban	Community	Primary Literature (peer reviewed publications)	To determine the outcome of stroke patients in Eastern cape	Concurrent Mixed Methods design	24 Stroke Patients (Quantitative Survey) 9 Stroke patients (Qualitative interviews)
18	De la Cornillère (2007)	Western cape	Urban	BLRC Rehab centre	Dissertations	To describe the range of experiences of stroke patients relating to attendance or non-attendance of those referred to the Bishop Lavis Rehabilitation centre stroke group	Mixed Methods Descriptive study	20 participants with stroke for questionnaire survey and 6 stroke participants for FGD.
19	De Villiers et al 2009	Cape Town	Urban	Secondary hospital	Primary Literature (peer reviewed publications)	To examine the impact of multidisciplinary stroke care on the in-hospital mortality,	Cross-sectional pre and post study design	195 stroke patients

For peer review only

						resource utilization, and access to inpatient rehabilitation facilities for stroke patients admitted in Stroke units at a secondary hospital in Cape Town, South Africa		
20	De Villiers 2011	Cape Town	Urban	District hospital	Primary Literature (peer reviewed publications)	To determine survival, disability and functional outcomes of stroke patients following their discharge from an acute stroke unit in an urban community with limited rehabilitative resources	Cross-sectional pre and post study design	196 stroke patients
21	Elloker (2015)	Western Cape	Urban	CHC	Dissertations	To determine participation restrictions and social support in patients with stroke, living in the Western Cape.	Mixed methods (Systematic Review + Quantitative survey)	106 stroke patients
22	Faux (2006)	Non-specific	Undefined	Undefined	Primary Literature (peer reviewed publications)	To provide a practical guide to helping stroke survivors who have a persistent disability maintain and enhance the gains made in rehabilitation	Narrative review recommendation	NA
23	Felemengas (2005)	Johannesburg Gauteng	Urban	Academic hospital	Dissertations	To investigate the family dynamics within	Qualitative	6 primary caregivers of stroke survivors

For peer review only

						the family system, as well as how these have evolved or changed following a stroke.		
24	Groenewald & Rhoda 2017	Western Cape	Urban	Non-Governmental facility	Primary Literature (peer reviewed publications)	To determine outcomes of stroke patients managed by a multidisciplinary team at a step-down facility in the Western Cape.	A longitudinal observational study	68 stroke patients
25	Groenewald (2018)	Western Cape	Urban	Step down rehabilitation facility	Dissertations	To adapt and contextualize the original UK Bridges stroke SMI workbook for implementation with the South African stroke population	A qualitative exploratory study - Interview, FGD, Expert consultation	13 Health care professionals<br>12 Stroke patients<br>Expert Panel
26	Hassan et al (2011)	Western cape	Urban	Western Cape Rehabilitation centre	Primary Literature (peer reviewed publications)	To explore levels of strain experienced by caregivers and the variables that impact on their strain.	Concurrent, mixed method, descriptive design	57 caregivers of stroke survivors
27	Hilton (2011)	Johannesburg	Urban	Community	Dissertations	To establish the functional level of patients, the level of strain and quality of life of the caregiver six to 36 months post-stroke, and the influence of demographic factors, caregiver strain and	Cross-sectional study	35 stroke patients and their caregivers

For peer review only

						patient's functional ability on quality of life of the caregiver.		
28	Hossain (2016)	Kwa-Zulu-Natal	Urban	Ladysmith Regional Hospital	Dissertations	To investigate the Need for Palliative care in Cerebrovascular Accident (stroke) patients at Ladysmith Regional Hospital	Mixed Methods (qualitative and quantitative)	72 stroke patients for quantitative study and 10 stroke patients for qualitative study
29	Joseph 2012	Western cape	Urban	WCRC – Rehabilitation centre	Dissertations	To determine the process of rehabilitation and the outcome of patients following in-patient rehabilitation at a facility in the Western Cape	A descriptive, observational, longitudinal design	76 Spinal Cord Injury patients and 67 stroke patients. Total patients (including drop outs) 130.
30	Kleineibst (2007)	Western Cape	Urban	Community	Dissertations	To determine the effectiveness of a caregiver support intervention programme to address the need for primary caregivers of stroke survivors in Bishop Lavis Community	Prospective descriptive qualitative study	29 caregivers of stroke survivors
31	Kotsokoane et al (2018)	Gauteng	Urban	CHC	Primary Literature (peer reviewed publications)	To determine the level of integration of stroke survivors at Soshanguve community clinics.	Retrospective quantitative research design	114 stroke survivors
32	Kusambiza-Kiingi (2016)	Johannesburg Gauteng	Urban	CHC	Primary Literature (peer reviewed publications)	To determine stroke survivors' levels of community	Cross-sectional study	108 stroke survivors and 45 caregivers

						reintegration, quality of life (QOL), satisfaction with the physiotherapy services and the level of caregiver strain at community health centres within the Johannesburg		
33	Leichtfuss (2009)	Western Cape	Urban	Private acute care hospitals	Dissertations	To examine the practice of doctors with regards to stroke rehabilitation in private acute-care hospitals private acute-care hospitals and to evaluate information shared between doctors and pts i.r.t prognosis, severity, discharge, referral, timing of discharge planning and decision making. in the Western Cape Metropole	Retrospective and descriptive design	37 doctors treating and discharging stroke patients 48 pts
34	Mabunda (2015)	Western Cape	Urban	Private not for profit Intermediate care Facility	Dissertations	To describe the model of service provision at an IC facility and the role it plays in the continuity of care in Cape Town.	Cross sectional survey	68 stroke patients 70 clinical staff
35	Makganye (2015)	Gauteng?	Urban	CHC	Dissertations	To investigate the physical, psychological,	Qualitative design	5 stroke patients and 5 caregivers

For peer review only

						social, religious		
36	Maleka et al (2012)	Limpopo and Gauteng	Urban and rural	Community	Primary Literature (peer reviewed publications)	To establish the experience of people living with stroke in low socioeconomic urban and rural areas of South Africa	Qualitative study design	32 stroke survivors living in the community
37	Mamabolo et al (2008)	Gauteng	Urban	PHC clinics	Primary Literature (peer reviewed publications)	to establish what demographic, environmental and physical factors influence functional independence post stroke.	Cross-sectional study	68 stroke patients
38	Mandizvidza (2017)	Western Cape	Urban	Level 1 2 3 hospitals	Dissertations	To describe the acute and post-acute ischaemic stroke services offered to ischaemic stroke patients in level 1, 2, and 3 hospitals in the Cape Metro Health District, compare these services to the national guideline and identify any barriers to optimum stroke patient care.	Descriptive cross-sectional study	10 doctors and 10 nurses from stroke ward and 8 doctors from emergency ward; pt records
39	Mashau et al 2016	Limpopo	Rural and Urban	HBC organization	Primary Literature (peer reviewed publications)	To investigate the impact of caregiving on voluntary home-based caregivers.	A quantitative cross-sectional descriptive survey	190 home-based caregivers

For peer review only - http://bmjopen.bmj.com/site/about/guidelines.xhtml

40	Masuku et al (2018)	Gauteng	Urban	Community	Primary Literature (peer reviewed publications)	To describe the caregiving experience of female caregivers of PWA residing in Tembisa, a township situated in the east of Johannesburg	Qualitative study	14 primary caregivers of stroke survivors with Aphasia
41	Matshikiza (2019)	Western cape	Urban	Tertiary Hospital	Dissertations	to determine the pre-hospital barriers and in-hospital delays to emergency care for patients presenting to Groote Schuur Hospital (GSH) with acute stroke.	prospective, observational study	50 patients with stroke
42	Mudzi (2010)	Gauteng	Urban	Community	Primary Literature (peer reviewed publications)	To establish the impact of caregiver education on the morbidity of the stroke survivors and on the quality of life of the stroke survivors and their carers.	A stratified randomised controlled trial	200 stroke patients and caregivers
43	Mudzi et al (2013)	Gauteng	Urban	Community	Dissertations	to establish the extent of community participation and the barriers and facilitators to the participation for stroke patients after their discharge.	longitudinal study	200 patients with first-time ischaemic stroke
44	Ntamo (2011)	Eastern Cape	Urban	Mthatha General	Dissertations	To identify factors that influence poor	Mixed methods (Qualitative +	85 stroke patients attending Physiotherapy at MGH

For peer review only - http://bmjopen.bmj.com/site/about/guidelines.xhtml

				Hospital		attendance for outpatient physiotherapy by patients discharged from MGH with a stroke.	Quantitative study)	
45	Parekh and Rhoda (2013)	Western Cape	Urban	Tertiary hospital	Primary Literature (peer reviewed publications)	To determine functional outcomes and factors influencing functional outcomes of stroke patients admitted to a South African tertiary hospital	Longitudinal Pre and Post test design	100 stroke patients
46	Parekh (2011)	Western Cape	Urban	Tertiary hospital	Dissertations	to identify factors influencing functional outcome of stroke patients admitted to a South African tertiary hospital	A descriptive, observational, longitudinal quantitative study design	66 stroke patients
47	Posner (2015)	Gauteng	Urban	Community	Dissertations	to explore the experiences and perceived needs of employed caregivers working for patients who have suffered from a stroke within home settings in South Africa.	Qualitative research design with Interviews + FGDs	15 employed caregivers working at the homes of stroke survivors<br>FGDs with 10 participants 5 in each group
48	Ras (2009)	Western Cape	Urban	NGO Run Hospital booth	Dissertations	to assess the quality of the stroke rehabilitation services at Booth Memorial Hospital.	Cross-sectional audit of records	NA
49	Rhoda (2009)	Western Cape	Peri - Urban	CHC	Primary Literature (peer reviewed publications)	to determine the structure and process of	Quantitative cross-sectional	100 first time stroke patients and 16 therapists

						rehabilitation of stroke patients at Community Health Centres (CHCs) in the Western Cape	survey	
50	Rhoda et al (2011)	Western Cape	Peri - Urban	CHC	Primary Literature (peer reviewed publications)	to determine the activity limitations of stroke patients receiving rehabilitation at out-patient Community health Centres (ChCs)	Longitudinal observational study	100 patients with stroke
51	Rhoda (2014)	Western Cape	Peri - Urban	CHC	Primary Literature (peer reviewed publications)	to determine the quality of life and factors influencing quality of life of community-dwelling stroke patients living in low-income, peri-urban areas in the Western Cape, South Africa.	Observational, longitudinal study	100 first time stroke patients
52	Rhoda et al (2015)	South Africa (Eastern Cape) Tanzania and Rwanda	Undefined	Provincial hospital	Primary Literature (peer reviewed publications)	the provision of inpatient rehabilitation and the post discharge challenges of stroke survivors in specific African countries.	Retrospective survey and interviews	168 SA stroke patients 145 Tanzanian and 130 Rwandan stroke patients<br>9 SA patients, 10 TP and 10 RP for qualitative study
53	Rouillard et al (2012)	Western Cape	Urban	WCRC rehab centre	Primary Literature (peer reviewed publications)	To determine activity limitations, participation restrictions, health-related quality of life and caregiver strain in community-dwelling	Longitudinal and descriptive study	46 stroke patients<br>41 caregivers

For peer review only - http://bmjopen.bmj.com/site/about/guidelines.xhtml

						stroke survivors discharged from an intensive inpatient rehabilitation programme at 6 months post stroke.		
54	SA-CSRG 2019	National	NA	NA	Primary literature (Peer-reviewed, Clinical Guideline)	contextualised development of stroke rehabilitation guideline	Guideline	NA
55	Scheffler and Mash 2019	Western Cape	Rural	Community	Primary Literature (peer reviewed publications)	To describe and analyze the outcomes of patients with stroke from a rural PHC setting in the Western Cape, South Africa.	Longitudinal survey	93 stroke patients
56	Smith (2019)	Western Cape	Western Cape	Urban and Rural	Community	To explore the self-management strategies employed by stroke survivors in the Western Cape, South Africa	Exploratory qualitative design (In-depth Interviews)	14 stroke survivors
57	Taylor & Ntusi (2019)	South Africa	South Africa	Undefined	Undefined	To improve management of stroke in South Africa	Editorial (Review)	NA
58	Thomas & Greenop (2008)	Gauteng	Gauteng	Urban	Community	To investigate into the complexities of caregiving, including both perceptions and experiences of the healthcare system.	Qualitative design (interviews)	6 caregivers of stroke survivors
59	Viljoen (2014)	Western Cape	Western Cape	Urban	Groote Schuur Hospital	To determine the cost of stroke care	Review of records	261 stroke patient (records)

						and to identify factors associated with increased expense, as well as to evaluate the quality of stroke care in general medical wards		
60	Wasserman et al 2009	KwaZulu-Natal	KwaZulu-Natal	Rural	Community	To assess discharge planning of stroke patients and to evaluate integration and continuity of stroke care between hospital and community	Quantitative design (Survey)	30 stroke patients

For peer review only

Supplementary file S3: Components assessed in included studies

**Author (year)**	**Governance/ Regulation**	**Resources**	**Service Delivery**	**Context**	**Re-organisation of care**	**Community engagement**
Arowoiya (2014)			X	X		
Bertram et al (2013)			X	X		
Bham & Ross 2005			X	X		
Biggs (2005)		X	X	X		X
Biggs & Rhoda (2008)				X		
Blackwell & Littlejohns (2010)			X			
Botha (2008)					X	X
Bryer (2009)		X	X			
Bryer et al (2010)	X					
Burton 2016					X	X
Cawood 2012		X	X			X
Cawood& Visagie (2015)			X			X
Cawood & Visagie (2016)			X			X
Cawood et al (2016)						x
Connor (2005)	X	X		X		
Cunningham (2012)		X	X	X		X
Cunningham & Rhoda (2014)						X
De la Cornillère (2007)			X	X		X
De Villiers et al (2009)			X			
De Villiers (2011)		X	X			
Elloker (2015)						X
Faux (2006)					X	
Felemengas (2005)			X	X		X
Groenewald & Rhoda (2017)			X			
Groenewald (2018)			X		X	
Hassan et al (2011)				X		
Hilton (2011)			X	X		
Hossain (2016)			X		X	
Joseph (2012)			X			X
Kleineibst (2007)		X	X	X		
Kotsokoane et al (2018)			X			X
Kusambiza-Kiingi (2016)			X	X		

For peer review only

Leichtfuss (2009)		X	X			X
Mabunda (2015)			X			
Makganye (2015)		X	X	X		
Maleka et al (2012)		X		X		X
Mamabolo et al (2008)						X
Mandizvidza (2017)	X	X	X			
Mashau et al (2016)			X			
Masuku et al (2018)			X	X		
Matshikiza (2019)		X	X			
Mudzi (2010)			X	X		X
Mudzi et al (2013)			X	X		
Ntamo (2011)		X	X	X		X
Parekh and Rhoda (2013)			X			
Parekh (2011)			X			
Posner (2015)			X			
Ras (2009)		X	X			
Rhoda (2009)		X	X			
Rhoda et al (2011)			X			
Rhoda (2014)			X	X		
Rhoda et al (2015)			X	X		
Rouillard et al (2012)			X	X		
SA-CSRG	X					
Scheffler and Mash (2019)			X			
Smith (2019)			X	X		
Taylor & Ntusi (2019)			X			
Thomas & Greenop (2008)			X	X		
Viljoen (2016)		X	X			
Wasserman et al (2009)			X			
**Total**	**4**	**16**	**47**	**24**	**5**	**19**

\]

For peer review only

DEFINITIONS

Universal health coverage - "Universal health coverage means that all people receive the health services they need, including public health services designed to promote better health (such as anti-tobacco information campaigns and taxes), prevent illness (such as vaccinations), and to provide treatment, rehabilitation and palliative care (such as end-of-life care) of sufficient quality to be effective, while at the same time ensuring that the use of these services does not expose the user to financial hardship (1)."

Health System - "A health system consists of all organizations, people and actions whose *primary intent* is to promote, restore or maintain health. This includes efforts to influence the determinants of health as well as more direct health-improving activities. A health system is, therefore, more than the pyramid of publicly owned facilities that deliver personal health services (2)."

Health system goals – "Health systems have multiple goals. The World health report 2000 defined overall health system outcomes or goals such as improving health and health equity, in ways that are responsive, financially fair, and make the best, or most efficient, use of available resources. There are also important intermediate goals: the route from inputs to health outcomes is through achieving greater access to and coverage for effective health interventions, without compromising efforts to ensure provider quality and safety (2)."

Health System building blocks – "To achieve their goals, all health systems have to carry out some basic functions, regardless of how they are organized: they have to provide services; develop health workers and other key resources; mobilize and allocate finances and ensure health system leadership and governance (also known as stewardship, which is about oversight and guidance of the whole system). For the purpose of clearly articulating what WHO will do to help strengthen health systems, the functions identified in the World health report 2000 have been broken down into a set of six essential 'building blocks'. All are needed to improve outcomes (2)."

Health system strengthening - Is defined as improving the six-health system building blocks and managing their interactions in ways that achieve more equitable and sustained improvements across health services and health outcomes. It requires both technical and political knowledge and action (2).

For peer review only

Stroke care- Is defined by the World Stroke Organisation as **"...**the continuum of care starting at the onset of a stroke event through the hyperacute phase, acute inpatient care, stroke rehabilitation, prevention of recurrent stroke and concludes with community reintegration and long-term recovery (3)." Supplementary

Clinical guideline - Clinical guidelines are statements that include recommendations intended to optimise patient care that are informed by a systematic review of evidence and an assessment of the benefits and harms of alternative care options (4).

References:

1. WHO, The World Bank. Tracking Universal Health Coverage: 2017 Global Monitoring Report. World Health Organisation. 2017;

2. Who. Everybody's business: strengthening health systems to improve health outcomes: WHO's framework for action. Production. 2007;

3. Lindsay MP, Norrving B, Furie KL, et al. Global stroke guidelines and action plan: a road map for quality stroke care [Internet]. Geneva; 2016 [cited 2021 Jan 10]. Available from: https://scholar.google.co.za/scholar?hl=en&as_sdt=0%2C5&q=Global+stroke+guidelines+and+action+plan%3A+a+road+map+for+quality+stroke+care&btnG=

4. Institute of Medicine. Clinical Practice Guidelines We Can Trust Developing Trustworthy Guidelines Setting Standards for Trustworthy Guidelines. Standards. 2011

For peer review only

BMJ Open

Achieving universal health coverage for people with stroke in South Africa: protocol for a scoping review

Sjan-Mari van Niekerk,[1] Gakeemah Inglis-Jassiem,[1] Sureshkumar Kamalakannan ,[2,3] Silke Fernandes,[4] Jayne Webster,[5] Rene English,[6] Tracey Smythe ,[3] QA Louw[1]

To cite: van Niekerk S-M, Inglis-Jassiem G, Kamalakannan S, *et al*. Achieving universal health coverage for people with stroke in South Africa: protocol for a scoping review. *BMJ Open* 2020;**10**:e041221. doi:10.1136/bmjopen-2020-041221

► Prepublication history and additional materila for this paper is available online. To view these files, please visit the journal online (http://dx.doi.org/10.1136/bmjopen-2020-041221).

Received 03 June 2020
Revised 08 September 2020
Accepted 09 September 2020

Check for updates

© Author(s) (or their employer(s)) 2020. Re-use permitted under CC BY. Published by BMJ.

For numbered affiliations see end of article.

Correspondence to
Dr Sjan-Mari van Niekerk;
smbrown@sun.ac.za

ABSTRACT

Introduction Stroke is the second most common cause of death after HIV/AIDS and a significant health burden in South Africa. The extent to which universal health coverage (UHC) is achieved for people with stroke in South Africa is unknown. Therefore, a scoping review to explore the opportunities and challenges within the South African health system to facilitate the achievement of UHC for people with stroke is warranted.

Methods and analysis The scoping review will follow the approach recommended by Levac, Colquhoun and O'Brien, which includes five steps: (1) identifying the research question, (2) identifying relevant studies, (3) selecting the studies, (4) charting the data, and (5) collating, summarising and reporting the results. Health Systems Dynamics Framework and WHO Framework on integrated people-centred health services will be used to map, synthesise and analyse data thematically.

Ethics and dissemination Ethical approval is not required for this scoping review, as it will only include published and publicly available data. The findings of this review will be published in an open-access, peer-reviewed journal and we will develop an accessible summary of the results for website posting and stakeholder meetings.

Strengths and limitations of this study

- ► A comprehensive scoping review methods is proposed to review the question.
- ► Frameworks such as the Health Systems Dynamics and integrated people-centred health services will be used.
- ► Global as well as national databases will be searched with comprehensive search strategies.
- ► The review will be limited to papers published between 2005 and 2020.
- ► The review will be limited to papers published in English and Afrikaans only.

INTRODUCTION

Stroke is the second most common cause of death after HIV/AIDS and a significant health burden in South Africa (SA)[1–4] ; an estimated 75 000 strokes occur each year in SA, with a projected burden of disease of 564 000 disability-adjusted life years.[5] The burden of stroke in rural areas of SA is also on the increase with an estimated 33 500 strokes occurring in 2011 in these areas alone.[6] However, the number of people living with stroke in SA is likely underestimated as no national stroke database or registry is in place, and these estimates are calculated from limited studies undertaken in a few parts of the country.

Stroke is the leading cause of disability in adults in SA, placing additional strain on social and healthcare services nationally.[7] Increased (1) prevalence of cardiovascular risk factors (such as hypertension, obesity, diabetes mellitus), (2) unchecked industrialisation and (3) urbanisation, contribute to this epidemiological transition of stroke in many low-income and middle-income countries,[8] including SA.[1] Cardiovascular diseases, including stroke, was previously set to surpass infectious diseases as the major cause of morbidity and mortality in sub-Saharan Africa[9] with the burden of these non-communicable diseases increasing in this region of Africa.[10] In the last decade, a growing body of evidence shows an association with non-communicable diseases and infectious diseases: for example, diabetes mellitus, chronic respiratory conditions and chronic kidney disease have been linked to increased tuberculosis (TB) morbidity and mortality and vice versa.[11] Non-communicable diseases may cause 'impaired immunity, metabolic imbalances and stress factors which favour the manifestation of TB'.[11] Furthermore, younger individuals in their most economically productive years are increasingly being affected in Africa, more often presenting with infectious comorbidities such as HIV/AIDS or TB in addition to stroke,[12 13] which further increases the burden on already strained health systems. With the recent COVID-19

BMJ Open: first published as 10.1136/bmjopen-2020-041221 on 12 October 2020. Downloaded from http://bmjopen.bmj.com/ on February 8, 2021 by guest. Protected by copyright.

For peer review only

For peer review only - http://bmjopen.bmj.com/site/about/guidelines.xhtml

global pandemic, it had become evident that individuals with underlying chronic conditions like stroke are more vulnerable to this infection and might have even more complex health and social care needs.[14]

Care for people with stroke

The delivery of effective and efficient stroke care is crucial to enhance the physical, cognitive and emotional well-being of individuals post-stroke, to improve their functional independence and quality of life, and ultimately to enable reintegration to their communities.[15 16] For the purposes of this review, we define stroke care as '…the continuum of care starting at the onset of a stroke event through the hyperacute phase, acute inpatient care, stroke rehabilitation, prevention of recurrent stroke, and concludes with community reintegration and long term recovery'.[17] Internationally, delivering effective stroke care has changed over the years as an understanding of stroke has increased, moving from individual care to a more comprehensive health system approach.[18]

The majority of people with stroke in SA may have no or limited access to stroke care and rehabilitation.[3 19] Stroke rehabilitation is limited in the first instance by insufficient rehabilitation facilities in the public sector, as well as inadequate intensity of inpatient rehabilitation, and lack of outpatient or community-based rehabilitation.[20] Reduced access to outpatient community-based stroke rehabilitation services was shown in a predominantly urban province of SA, where people with stroke only received 1–4 hours of physiotherapy sessions with a median number of 1.8 hours over a 6-month period.[21] In SA, stroke care occurs across a range of settings, from tertiary hospitals to remote community primary healthcare facilities; care for people with stroke can be provided individually or in a group setting, at home, in a community environment or a specialist centre.[1] While public health policy in SA ascribes to primary healthcare and a decentralised approach, many stroke care and rehabilitation services remain centralised at district and specialist rehabilitation hospitals,[22] which reinforces the inequality experienced by rural communities in terms of healthcare access.[5] Although home-based care services are available in certain communities, poor referral to and articulation with these services and other healthcare levels, often result in poor service delivery.[23] Individuals who experience mobility limitations post-stroke subsequently experience restricted access to services, as do those with minimal access to transport. Free primary level rehabilitation services are available in some communities, however these services may not be comprehensive or efficient due to shortages of staff skilled in stroke care or incomplete multidisciplinary teams.[22] People with stroke may be discharged home without receiving rehabilitation interventions, and families experience both catastrophic health expenditure and financial loss due to additional responsibilities of caregiving, often leading to an inability to maintain gainful employment.[3 24] As in other low-income and middle-income countries, unmet stroke care and rehabilitation needs are further entrenched due to limited financial and infrastructural/architectural resources.[20 24] In addition, in SA there are no dedicated support systems for caregivers and people with stroke, which is compounded by a population that experience low levels of health literacy.[21 25 26] Health-seeking behaviours may be further affected by the influence of religion and cultural beliefs on patients' views regarding the causes and management of cardiovascular conditions such as stroke, which highlights the need for culturally sensitive stroke care and patient-centric health systems.[27]

Status of stroke care in SA

The sociopolitical context of SA has had significant effects on national healthcare policies and services, largely due to disparities in social and financial capital based on discriminatory racial and gender divides.[4] Although classified as a middle-income country, SA has high levels of poverty and unemployment, with unemployment rates ranging between 29% (nationally) and 50% (among younger people) and relative poverty worsening over time (Gini coefficient increased from 0.6 in 1995 to almost 0.7 in 2009).[28] These social determinants of health influence the high levels of mortality and morbidity brought about by infectious diseases such as HIV and TB, as well as non-communicable diseases such as diabetes and hypertension.[4 29] Since the first democratic election in 1994, both government and society strive for equality and adopted a human rights-based approach to healthcare reform. Despite well-founded rights-based policy development with practical application, many areas still struggle to achieve equality.[29] The SA government has committed to the WHO vision of achieving equitable, evidence-based rehabilitation for all by 2030,[30] however it remains 'unclear how many South Africans access and receive rehabilitation services after sustaining a stroke, what rehabilitation is provided to them, how effective this rehabilitation is and what the implications are, of receiving inadequate, or even no, rehabilitation'.[26]

Universal health coverage for people with stroke in SA

The proposed National Health Insurance (NHI) bill, currently being considered by the National Assembly of SA[31] focuses on primary healthcare as the foundation for universal health coverage (UHC), however the bill may not provide coverage for most age-related health conditions such as mobility impairments, and visual or auditory impairments.[32] UHC occurs where everyone receives essential services, according to their need and without financial hardship.[33] Essential services are defined as promotion, prevention, treatment, rehabilitation (including assistive technology) and palliative care. Key features of UHC include its human rights-focused underpinning and an integrated approach to health service delivery. It also importantly recognises the role that health system functioning plays in the realisation of UHC.[34] In recent years, SA has spearheaded radical healthcare policy reform to facilitate patient-centred and

BMJ Open: first published as 10.1136/bmjopen-2020-041221 on 12 October 2020. Downloaded from http://bmjopen.bmj.com/ on February 8, 2021 by guest. Protected by copyright.

For peer review only - http://bmjopen.bmj.com/site/about/guidelines.xhtml

accessible service delivery as outlined in the Healthcare 2030 plan of the National Department of Health.[26] This has mainly been actioned via a decentralised district level healthcare service delivery model and the development of an NHI scheme to curtail financial hardship experienced by users.[35] Moving towards UHC is a priority in SA and requires a strong and responsive healthcare delivery system to anchor effective and efficient service delivery. There is a crucial need for 'good leadership, stewardship, and management of health and related services to achieving health for all people'.[4] Louw *et al* further affirm that 'improving capacity across African healthcare settings is essential to ensure best practice health programmes to increase awareness of stroke and its causes, its early identification and acute management, and its rehabilitation'.[36]

In general, stroke care services in Africa are not adequately supported by governments, with limited support for either the development or implementation of national stroke policy frameworks, or limited provision and endorsement of evidence-based clinical practice guidelines.[37] Where policy frameworks or practice guidelines are available, the implementation processes may not be well supported by states,[38] which further limits best practice care for people with stroke.[37] In addition, uniquely African clinical practice guidelines for stroke care have not been well reported.[39 40] Practice guidelines that are appropriately adapted for local context are important, and consequently, the South African-contextualised Stroke Rehabilitation Guideline,[22] developed in collaboration with key stakeholders, including the South African Department of Health, were developed between 2017 and 2018. This guideline marked a strategic and collaborative increased focus on the importance and quality of stroke rehabilitation in the SA healthcare context. However, the update of such guidelines in at all levels of care remain a challenge.

Accessible, responsive and quality stroke care services within a strengthened local health system will contribute to UHC for people with stroke and their caregivers in SA. The extent to which UHC is achieved for people with stroke in SA is unknown and relatively little is known about the opportunities and challenges within the local health system to achieve UHC.

Aim of this scoping review

The aim of this review is to explore the opportunities and challenges within the South African health system to facilitate the achievement of UHC for people with stroke. A scoping review method was chosen to address the broad nature of our question as well as identify knowledge gaps to inform further research. For the purpose of this scoping review, we will specifically explore the components and characteristics of the stroke-related health system as well as policies that potentially facilitate or hinder stroke care. We will draw on two frameworks: Health Systems Dynamics Framework (HSDF)[41] and WHO Framework on integrated people-centred health services (IPCHS)[42] to map the data from different sources in a thematic content analysis. This information is required to adequately plan for the healthcare needs of people with stroke in the local SA context.

METHODS

We will conduct this scoping review according to the approach recommended by Levac *et al*,[43] which includes five steps: (1) identifying the research question, (2) identifying relevant studies, (3) selecting the studies, (4) charting the data, and (5) collating, summarising and reporting the results. The review will be reported according to the Preferred Reporting Items for Systematic Reviews and Meta-Analyses extension for Scoping Reviews (PRISMA-ScR) guidelines using a modified PRISMA flow chart.[44]

Analytical framework

Our review will be guided by an analytical framework adapted from the HSDF[40] and WHO Framework on IPCHS)[41] to map and synthesise data from different sources in a thematic content analysis. The HSDF incorporates the WHO health system building blocks (1) service delivery; (2) health workforce; (3) information; (4) medical products, vaccines and technologies; (5) financing; and (6) leadership and governance, and highlights how values and principles drive the behaviour of people when making choices and engaging with processes of any health system. This HSDF framework offers an integrated view of a health system in that it acknowledges the social, economic, political context and determinants of health. The WHO IPCHS aims to support countries to achieve UHC by facilitating access to health services that are provided in a way that is coordinated around people's needs, respects their preferences, and are safe, effective, timely, affordable, and of acceptable quality. Our analytical framework will include all of the HSDF components and two components from the IPCHS: (1) re-orientation of care and (2) enabling environment, which are appropriate to the SA context and population (see figure 1).

Step 1: defining the review objectives

In line with the purpose of scoping reviews, our approach is broad, with emphasis on studies that investigate any aspect of the healthcare system in regard to stroke care in SA. The definitions of the concepts that will be used for this review is provided in online supplemental file 1.

The review objectives are to:

1. Describe the health system-related factors such as governance, resources, community engagement and service delivery that support and guide stroke care in SA.
2. Describe the health system-related factors such as governance, resources, community engagement that limit the achievement of universal stroke care in SA.
3. Synthesise the findings to gain insights into the driving factors that will fundamentally bring required change to achieve universal stroke care in SA.

For peer review only

For peer review only - http://bmjopen.bmj.com/site/about/guidelines.xhtml

BMJ Open: first published as 10.1136/bmjopen-2020-041221 on 12 October 2020. Downloaded from http://bmjopen.bmj.com/ on February 8, 2021 by guest. Protected by copyright.

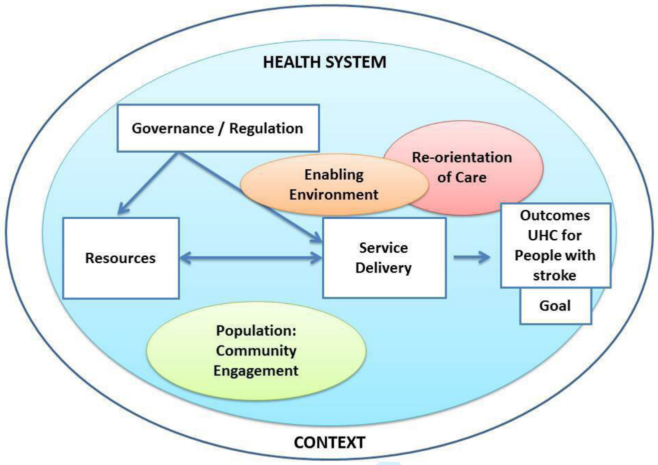

Figure 1 Analytical framework (adapted from the Health Systems Dynamics Framework[41] and the WHO Framework on integrated people-centred health services components[42]). UHC, universal health coverage.

Step 2: identifying relevant studies

Search strategy

A comprehensive search, with the most appropriate search terms per database, will be conducted for each of the following databases: PubMed, EBSCOhost (including Medline and CINAHL), Scopus, Global Health and African-wide databases (including EBSCOhost Africa Wide, African Journals Sabinet and African Journals Online). The databases were chosen to ensure that all relevant literature was identified. An initial, limited search selection of relevant databases will take place, followed by an analysis of text words in the title and abstract, as well as the index terms used to describe the article. To ensure that all relevant studies are included, additional, peer-reviewed literature will be added by hand searching the reference lists of the articles that were initially included to ensure that articles have not been missed. The search strategy developed for the purpose of the proposed review is provided in online supplemental file 2.

Grey literature will be identified by using the WHO's OpenGrey and OpenDOAR Library, as well as sources such as Open Access Theses and Dissertations, Sabinet: Current and Completed Research, ProQuest Dissertations and Theses, Database of African Theses and Dissertations, and the Networked Digital Library of Theses and Dissertations to include research conducted on stroke care in SA. The websites of relevant government and service provider agencies will be searched to identify relevant South African stroke care policy documents and/or practice guidance documents. In addition, field experts will be contacted to identify additional relevant evidence regarding stroke care in SA. Saturation will be the point at which no new evidence is found to be included in the scoping review.

A Boolean search string has been developed through the systematic process of reviewing Medical Subject Heading terms on Medline. A key term search strategy will be employed using a variety of combinations of terms for 'stroke', AND 'health system' OR 'Universal Health Coverage' OR 'clinical practice guideline' AND 'South Africa'. We will also conduct specific key term searches using each of the seven different components from the analytic framework (figure 1), combined with 'stroke' and 'South Africa'. We have conducted a pilot search in Google Scholar on the 9th of May 2020, to demonstrate the feasibility of answering our research question using a scoping review method. For the pilot search we have used the following combination of search terms 'stroke' AND 'South Africa' AND 'health system' OR 'Universal Health Coverage' OR 'clinical practice guideline' -cardiovascular -diabetes', which resulted in 2440 hits and a possible 100 publications were retrieved by title.

Eligibility criteria

This scoping review will include primary and secondary research studies, policy documents, reports and clinical practice guidelines describing any aspect of the health-care system for people with stroke in SA. The review will include qualitative, quantitative and mixed methods studies of any study design. Records published or made available between 2005 and 2020 will be accepted for inclusion. A national reform of the SA healthcare system was called on in 2009, thus this timeframe would ensure that we include all possible studies that focused on the functioning of the SA health system since the time of reform until the present moment.[45]

Records will be limited to those taking place in SA. Only records where the full text is available (in English or Afrikaans) will be included. This is especially because the research team possess these language skills and its contextually relevant. Finally, evidence will be interrogated as to whether they addressed at least one of the components of the analytical framework.

Step 3: evidence selection

Following database search, retrieved articles will be screened in three stages. First, one reviewer will independently screen the titles and abstracts of identified studies to exclude publications that do not meet the inclusion criteria. A second reviewer will check the results for accuracy. The data will be managed with EndNote V.X8. The full-text article will be retrieved for review if the citation is deemed eligible by at least one reviewer. Two of these reviewers will independently assess each article against the inclusion criteria. Any discrepancies between the reviewers will be resolved by discussion, and a third reviewer will be consulted if necessary. A modified PRISMA that incorporates PRISMA-ScR reporting framework will be completed to summarise the study selection process.

Step 4: data charting

Data extraction will be undertaken independently by three reviewers (S-MvN, GI-J, SK). Each reviewer will be allocated specific data item(s) to extract, a second reviewer will check the extracted data for accuracy. Before

BMJ Open: first published as 10.1136/bmjopen-2020-041221 on 12 October 2020. Downloaded from http://bmjopen.bmj.com/ on February 8, 2021 by guest. Protected by copyright.

For peer review only - http://bmjopen.bmj.com/site/about/guidelines.xhtml

Table 1 Application of the key characteristics of the analytical framework

Framework component	Description (data items)
Governance/regulation	Healthcare policies at national or provincial levels; resource allocation policies; accountability; coordination and regulations.
Resources	Infrastructure (accessible; equipped and maintained); finance allocation and affordability; human resources (availability; distribution—occupation/specialisation, place of work, gender; graduates of health professionals); knowledge (clinical decision-making; clinical guidelines).
Service delivery	Service delivery models; health services and service providers (private/public, for-profit or not–for-profit, formal or informal, professional or non-professional, allopathic or traditional, remunerated or voluntary).
Context	Socioeconomic, technological, cultural, political and environmental environments.
Reorientation of care	Health promotion and patient education. New technologies (eHealth; shared electronic medical records; telemedicine; m-health). Coordination of health services; continuous healthcare, multiprofessional health teams; comprehensive health services and intersectoral coordination.
Enabling environment	Health workforce allocation/shortages; distribution of health workforce; workforce training; clearly defined roles and fair wages
Population: community engagement	Engaging and empowering individuals, families, communities and informal carers. Reaching underserved and marginalised communities. Common decision-making, self-efficacy of patients.

commencing data extraction, the reviewers will first discuss the information to be extracted to ensure clarity of the data extraction process. Any discrepancies between the reviewers will be resolved by discussion, and a third reviewer will be consulted if necessary. A custom-designed form will be developed in Excel for data charting. Two independent reviewers will pilot the data charting form using a random sample of five included studies for consistency and required amendments will be agreed by consensus. We will modify the data extraction form as required based on feedback from the two reviewers, and the form amended at each stage where necessary. We plan to contact study authors in the case of unclear information and will make up to three attempts by email.

Data items

The following general information will be extracted and tabulated from the included articles: author name(s), publishing journal, year of publication, region in SA, study population, the study aims/objectives/question, the setting, study-design and findings. In addition, the following data will be extracted according to the components of HSDF and the WHO's IPCHS framework components. To ensure consistency of the data extraction process, we will consider the seven components using the descriptions in table 1.

Data synthesis and analysis

The analytical framework will guide the data synthesis and thematic analysis. We anticipate that the dataset will include different study designs, and therefore both descriptive statistics and narrative synthesis will be used. We will establish the strengths and opportunities that should be optimised in order to improve UHC of stroke services in SA as well as the weaknesses and threats which need to be minimised or eliminated.

Availability of data and materials

All data generated or analysed during this study will be included in the published scoping review article.

Patient and public involvement

No patients and public were involved for the purpose of this scoping review.

Ethics and dissemination

Ethical approval is not required for this scoping review, as it will only include published and publicly available data. The findings of this review will be published in an open-access, peer-reviewed journal and we will develop an accessible summary of the results for website posting and stakeholder meetings.

Author affiliations
[1]Division of Physiotherapy, Department of Health and Rehabilitation Sciences, Stellenbosch University, Cape Town, South Africa
[2]SACDIR, Public Health Foundation of India, Gurgaon, India
[3]International Center for Evidence in Disability, London School of Hygiene and Tropical Medicine, London, UK
[4]Department of Global Health and Development, Faculty of Public Health and Policy, London School of Hygiene and Tropical Medicine, London, UK
[5]Disease Control Department, Faculty of Infectious and Tropical Diseases, London School of Tropical Health and Medicine, London, UK
[6]Division of Health Systems and Public Health, Department of Global Health, Faculty of Medicine and Health Sciences, Stellenbosch University, Cape Town, South Africa

Correction notice The article has been corrected since it is published. The affiliations 5 and 6 have been updated.

Contributors S-MvN and GI-J drafted and revised the protocol with suggestions from TS, JW, SK, SF, RE and QAL who reviewed the protocol and provided feedback on the draft. S-MvN, SF and GI-J in consultation with the other authors constructed the search. All authors read and approved the final protocol.

Funding This research was commissioned by the National Institute for Health Research (NIHR) Global Health Policy and Systems Research Development Award using UK aid from the UK government. Grant number NIHR130180.

Disclaimer The views expressed in this publication are those of the author(s) and not necessarily those of the NIHR or the Department of Health and Social Care.

Competing interests None declared.

Patient and public involvement Patients and/or the public were not involved in the design, or conduct, or reporting, or dissemination plans of this research.

Patient consent for publication Not required.

Provenance and peer review Not commissioned; externally peer reviewed.

Open access This is an open access article distributed in accordance with the Creative Commons Attribution 4.0 Unported (CC BY 4.0) license, which permits others to copy, redistribute, remix, transform and build upon this work for any purpose, provided the original work is properly cited, a link to the licence is given, and indication of whether changes were made. See: https://creativecommons.org/licenses/by/4.0/.

ORCID iDs

For peer review only - http://bmjopen.bmj.com/site/about/guidelines.xhtml

BMJ Open: first published as 10.1136/bmjopen-2020-041221 on 12 October 2020. Downloaded from http://bmjopen.bmj.com/ on February 8, 2021 by guest. Protected by copyright.

Sureshkumar Kamalakannan http://orcid.org/0000-0003-4407-7838
Tracey Smythe http://orcid.org/0000-0003-3408-7362

REFERENCES

1 Scheffler E, Mash R. Surviving a stroke in South Africa: outcomes of home-based care in a low-resource rural setting. *Top Stroke Rehabil* 2019;26:423–34.
2 Pillay-van Wyk V, Msemburi W, Laubscher R, *et al*. Mortality trends and differentials in South Africa from 1997 to 2012: second National burden of disease study. *Lancet Glob Health* 2016;4:e642–53.
3 Maredza M, Bertram MY, Gómez-Olivé XF, *et al*. Burden of stroke attributable to selected lifestyle risk factors in rural South Africa. *BMC Public Health* 2016;16:143.
4 Coovadia H, Jewkes R, Barron P, *et al*. The health and health system of South Africa: historical roots of current public health challenges. *Lancet* 2009;374:817–34.
5 Bertram MY, Katzenellenbogen J, Vos T, *et al*. The disability adjusted life years due to stroke in South Africa in 2008. *Int J Stroke* 2013;8:76–80.
6 Maredza M, Bertram MY, Tollman SM. Disease burden of stroke in rural South Africa: an estimate of incidence, mortality and disability adjusted life years. *BMC Neurol* 2015;15:54.
7 The Heart and Stroke Foundation. Salt is killing South Africans, 2015. Available: https://www.health24.com/Diet-and-nutrition/News/Salt-is-killing-South-Africans-20130312
8 Kabudula CW, Houle B, Collinson MA, *et al*. Progression of the epidemiological transition in a rural South African setting: findings from population surveillance in Agincourt, 1993-2013. *BMC Public Health* 2017;17:424.
9 Yusuf S, Reddy S, Ôunpuu S, *et al*. Global burden of cardiovascular diseases: part I: general considerations, the epidemiologic transition, risk factors, and impact of urbanization. *Circulation* 2001;104:2746–53.
10 Owolabi MO, Akarolo-Anthony S, Akinyemi R, *et al*. The burden of stroke in Africa: a glance at the present and a glimpse into the future. *Cardiovasc J Afr* 2015;26:S27–38.
11 Puchner KP, Rodriguez-Fernandez R, Oliver M, *et al*. Non-communicable diseases and tuberculosis: Anticipating the impending global storm. *Glob Public Health* 2019;14:1372–81.
12 Zimba S, Ntanda PM, Lakhi S, *et al*. HIV infection, hypercoagulability and ischaemic stroke in adults at the University teaching hospital in Zambia: a case control study. *BMC Infect Dis* 2017;17:354.
13 Heikinheimo T, Chimbayo D, Kumwenda JJ, *et al*. Stroke outcomes in Malawi, a country with high prevalence of HIV: a prospective follow-up study. *PLoS One* 2012;7:e33765.
14 Wu Z, McGoogan JM. Characteristics of and Important Lessons From the Coronavirus Disease 2019 (COVID-19) Outbreak in China: Summary of a Report of 72 314 Cases From the Chinese Center for Disease Control and Prevention. *JAMA* 2020;323:1239–42.
15 Kitzman P, Hudson K, Sylvia V, *et al*. Care coordination for community transitions for individuals post-stroke returning to low-resource rural communities. *J Community Health* 2017;42:565–72.
16 Langhorne P, Bernhardt J, Kwakkel G. Stroke rehabilitation. *Lancet* 2011;377:1693–702.
17 Lindsay MP, Norrving B, Furie KL, *et al*. *Global stroke guidelines and action plan: a road map for quality stroke care*. Geneva: World Stroke Organization, 2016.
18 Miller KK, Lin SH, Neville M. From hospital to home to participation: a position paper on transition planning poststroke. *Arch Phys Med Rehabil* 2019;100:1162–75.
19 Ntsiea MV. Current stroke rehabilitation services and physiotherapy research in South Africa. *S Afr J Physiother* 2019;75:475.
20 Bryer A, Connor MD, Haug P, *et al*. The South African guideline for the management of ischemic stroke and transient ischemic attack: recommendations for a resource-constrained health care setting. *Int J Stroke* 2011;6:349–54.
21 Rhoda A. The rehabilitation of stroke patients at community health centres in the Western Cape (Doctoral dissertation, University of the Western Cape).
22 South African Contextualised stroke rehabilitation guideline (SA-CSRG), 2019. Available: http://www.sun.ac.za/english/faculty/healthsciences/health-rehabilitation-sciences/Documents/Completed%20stroke%20guidelines_May2019.pdf
23 A Mabunda S, London L, Pienaar D. An evaluation of the role of an intermediate care facility in the continuum of care in Western Cape, South Africa. *Int J Health Policy Manag* 2018;7:167–79.
24 Pandian JD, William AG, Kate MP, *et al*. Strategies to improve stroke care services in low- and middle-income countries: a systematic review. *Neuroepidemiology* 2017;49:45–61.
25 Visagie S, Swartz L. Rural South Africans' rehabilitation experiences: case studies from the Northern Cape Province. *S Afr J Physiother* 2016;72:298.
26 Western Cape Government. *Healthcare 2030: the road to wellness*. Cape Town: Western Cape Department of Health, 2014.
27 Osokpo O, Riegel B. Cultural factors influencing self-care by persons with cardiovascular disease: an integrative review. *Int J Nurs Stud* 2019;9:103383.
28 Benatar S, Gill S. Universal access to healthcare: the case of South Africa in the comparative global context of the late Anthropocene era. *Int J Health Policy Manag* 2020
29 van den Heever AM. South Africa's universal health coverage reforms in the post-apartheid period. *Health Policy* 2016;120:1420–8.
30 World Health Organisation. *Tracking universal health coverage: 2017 global monitoring report. World Health organization and international bank for reconstruction and development / the world bank*, 2017.
31 National health insurance bill. Available: https://www.gov.za/documents/national-health-insurance-bill-b-11-2019-6-aug-2019-0000
32 McIntyre D, Garshong B, Mtei G, *et al*. Beyond fragmentation and towards universal coverage: insights from Ghana, South Africa and the United Republic of Tanzania. *Bull World Health Organ* 2008;86:871–6.
33 World Health Organisation. *Health systems financing: the path to universal coverage. World health report 2010*. Geneva: WHO, 2010a.
34 Abiiro GA, De Allegri M. Universal health coverage from multiple perspectives: a synthesis of conceptual literature and global debates. *BMC Int Health Hum Rights* 2015;15:17.
35 Fusheini A, Eyles J. Achieving universal health coverage in South Africa through a district health system approach: conflicting ideologies of health care provision. *BMC Health Serv Res* 2016;16:558.
36 Louw Q, Twizeyemariya A, Grimmer K, *et al*. Estimating the costs and benefits of stroke rehabilitation in South Africa. *J Eval Clin Pract* 2020;26:1181–7.
37 Baatiema L, Otim M, Mnatzaganian G, *et al*. Towards best practice in acute stroke care in Ghana: a survey of hospital services. *BMC Health Serv Res* 2017;17:108.
38 Cockburn L, Fanfon TN, Bramall A, *et al*. Best practice guidelines for stroke in Cameroon: an innovative and participatory knowledge translation project. *Afr J Disabil* 2014;3:92.
39 Okwen PM, Maweu I, Grimmer K, *et al*. Evaluation of all African clinical practice guidelines for hypertension: quality and opportunities for improvement. *J Eval Clin Pract* 2019;25:565–74.
40 Grimmer K, Louw Q, Dizon JM, *et al*. Standardising evidence strength grading for recommendations from multiple clinical practice guidelines: a South African case study. *Implement Sci* 2018;13:117.
41 Olmen JV, Criel B, Bhojani U, *et al*. The health system dynamics framework: the introduction of an analytical model for health system analysis and its application to two case-studies. *Hcs* 2012;2:1–21.
42 World Health Organization. Framework on integrated, people-centred health services. Report by the Secretariat. 69th World health assembly provisional agenda item 2016;A69.
43 Levac D, Colquhoun H, O'Brien KK. Scoping studies: advancing the methodology. *Implementation Sci* 2010;5:69.
44 Tricco AC, Lillie E, Zarin W, *et al*. PRISMA extension for scoping reviews (PRISMA-ScR): checklist and explanation. *Ann Intern Med* 2018;169:467–73.
45 Mayosi BM, Lawn JE, Van Niekerk A, *et al*. Lancet South Africa team. Health in South Africa: changes and challenges since 2009. *Lancet* 2012;380:2029–43.

For peer review only

BMJ Open: first published as 10.1136/bmjopen-2020-041221 on 12 October 2020. Downloaded from http://bmjopen.bmj.com/ on February 8, 2021 by guest. Protected by copyright.

For peer review only - http://bmjopen.bmj.com/site/about/guidelines.xhtml

Preferred Reporting Items for Systematic reviews and Meta-Analyses extension for Scoping Reviews (PRISMA-ScR) Checklist

SECTION	ITEM	PRISMA-ScR CHECKLIST ITEM	REPORTED ON PAGE #
**TITLE**			
Title	1	Identify the report as a scoping review.	1
**ABSTRACT**			
Structured summary	2	Provide a structured summary that includes (as applicable): background, objectives, eligibility criteria, sources of evidence, charting methods, results, and conclusions that relate to the review questions and objectives.	2
**INTRODUCTION**			
Rationale	3	Describe the rationale for the review in the context of what is already known. Explain why the review questions/objectives lend themselves to a scoping review approach.	4
Objectives	4	Provide an explicit statement of the questions and objectives being addressed with reference to their key elements (e.g., population or participants, concepts, and context) or other relevant key elements used to conceptualize the review questions and/or objectives.	4
**METHODS**			
Protocol and registration	5	Indicate whether a review protocol exists; state if and where it can be accessed (e.g., a Web address); and if available, provide registration information, including the registration number.	6
Eligibility criteria	6	Specify characteristics of the sources of evidence used as eligibility criteria (e.g., years considered, language, and publication status), and provide a rationale.	6
Information sources*	7	Describe all information sources in the search (e.g., databases with dates of coverage and contact with authors to identify additional sources), as well as the date the most recent search was executed.	6
Search	8	Present the full electronic search strategy for at least 1 database, including any limits used, such that it could be repeated.	6
Selection of sources of evidence†	9	State the process for selecting sources of evidence (i.e., screening and eligibility) included in the scoping review.	6
Data charting process‡	10	Describe the methods of charting data from the included sources of evidence (e.g., calibrated forms or forms that have been tested by the team before their use, and whether data charting was done independently or in duplicate) and any processes for obtaining and confirming data from investigators.	6
Data items	11	List and define all variables for which data were sought and any assumptions and simplifications made.	6-7
Critical appraisal of individual sources of evidence§	12	If done, provide a rationale for conducting a critical appraisal of included sources of evidence; describe the methods used and how this information was used in any data synthesis (if appropriate).	7
Synthesis of results	13	Describe the methods of handling and summarizing the data that were charted.	7

St. Michael's
Inspired Care.
Inspiring Science.

For peer review only - http://bmjopen.bmj.com/site/about/guidelines.xhtml

SECTION	ITEM	PRISMA-ScR CHECKLIST ITEM	REPORTED ON PAGE #
**RESULTS**			
Selection of sources of evidence	14	Give numbers of sources of evidence screened, assessed for eligibility, and included in the review, with reasons for exclusions at each stage, ideally using a flow diagram.	7-17
Characteristics of sources of evidence	15	For each source of evidence, present characteristics for which data were charted and provide the citations.	7-17
Critical appraisal within sources of evidence	16	If done, present data on critical appraisal of included sources of evidence (see item 12).	7-17
Results of individual sources of evidence	17	For each included source of evidence, present the relevant data that were charted that relate to the review questions and objectives.	7-17
Synthesis of results	18	Summarize and/or present the charting results as they relate to the review questions and objectives.	7-17
**DISCUSSION**			
Summary of evidence	19	Summarize the main results (including an overview of concepts, themes, and types of evidence available), link to the review questions and objectives, and consider the relevance to key groups.	17
Limitations	20	Discuss the limitations of the scoping review process.	21
Conclusions	21	Provide a general interpretation of the results with respect to the review questions and objectives, as well as potential implications and/or next steps.	21
**FUNDING**			
Funding	22	Describe sources of funding for the included sources of evidence, as well as sources of funding for the scoping review. Describe the role of the funders of the scoping review.	22

JBI = Joanna Briggs Institute; PRISMA-ScR = Preferred Reporting Items for Systematic reviews and Meta-Analyses extension for Scoping Reviews.
* Where *sources of evidence* (see second footnote) are compiled from, such as bibliographic databases, social media platforms, and Web sites.
† A more inclusive/heterogeneous term used to account for the different types of evidence or data sources (e.g., quantitative and/or qualitative research, expert opinion, and policy documents) that may be eligible in a scoping review as opposed to only studies. This is not to be confused with *information sources* (see first footnote).
‡ The frameworks by Arksey and O'Malley (6) and Levac and colleagues (7) and the JBI guidance (4, 5) refer to the process of data extraction in a scoping review as data charting.
§ The process of systematically examining research evidence to assess its validity, results, and relevance before using it to inform a decision. This term is used for items 12 and 19 instead of "risk of bias" (which is more applicable to systematic reviews of interventions) to include and acknowledge the various sources of evidence that may be used in a scoping review (e.g., quantitative and/or qualitative research, expert opinion, and policy document).

From: Tricco AC, Lillie E, Zarin W, O'Brien KK, Colquhoun H, Levac D, et al. PRISMA Extension for Scoping Reviews (PRISMAScR): Checklist and Explanation. Ann Intern Med. 2018;169:467–473. doi: 10.7326/M18-0850.

St. Michael's
Inspired Care.
Inspiring Science.

For peer review only - http://bmjopen.bmj.com/site/about/guidelines.xhtml

BMJ Open

Towards universal health coverage for people with stroke in South Africa: a scoping review

Journal:	*BMJ Open*
Manuscript ID	bmjopen-2021-049988.R1
Article Type:	Original research
Date Submitted by the Author:	22-Aug-2021
Complete List of Authors:	Sjan-Mari, van Niekerk; Stellenbosch University, Division of Physiotherapy, Department of Health and Rehabilitation Sciences<br>Sureshkumar, K; Public Health Foundation of India, SACDIR; London School of Hygiene & Tropical Medicine, International Center for Evidence in Disability<br>Gakeemah, Inglis-Jassiem; Stellenbosch University, Division of Physiotherapy, Department of Health and Rehabilitation Sciences<br>Charumbira, Maria; Stellenbosch University Faculty of Medicine and Health Sciences, Rehabilitation Sciences<br>Fernandes, Silke; London School of Hygiene and Tropical Medicine,<br>Webster, Jayne; London School of Hygiene and Tropical Medicine,<br>English, Rene; Stellenbosch University Faculty of Medicine and Health Sciences, Global Health<br>Louw, QA; Stellenbosch University, Physiotherapy; Stellenbosch University,<br>Smythe, Tracey; London School of Hygiene and Tropical Medicine, Clinical Research
**Primary Subject Heading**:	Public health
Secondary Subject Heading:	Health policy, Health services research, Rehabilitation medicine, Research methods
Keywords:	Stroke < NEUROLOGY, PUBLIC HEALTH, Health policy < HEALTH SERVICES ADMINISTRATION & MANAGEMENT, Rehabilitation medicine < INTERNAL MEDICINE, PRIMARY CARE

SCHOLARONE™
Manuscripts

BMJ

I, the Submitting Author has the right to grant and does grant on behalf of all authors of the Work (as defined in the below author licence), an exclusive licence and/or a non-exclusive licence for contributions from authors who are: i) UK Crown employees; ii) where BMJ has agreed a CC-BY licence shall apply, and/or iii) in accordance with the terms applicable for US Federal Government officers or employees acting as part of their official duties; on a worldwide, perpetual, irrevocable, royalty-free basis to BMJ Publishing Group Ltd ("BMJ") its licensees and where the relevant Journal is co-owned by BMJ to the co-owners of the Journal, to publish the Work in this journal and any other BMJ products and to exploit all rights, as set out in our licence.

The Submitting Author accepts and understands that any supply made under these terms is made by BMJ to the Submitting Author unless you are acting as an employee on behalf of your employer or a postgraduate student of an affiliated institution which is paying any applicable article publishing charge ("APC") for Open Access articles. Where the Submitting Author wishes to make the Work available on an Open Access basis (and intends to pay the relevant APC), the terms of reuse of such Open Access shall be governed by a Creative Commons licence – details of these licences and which Creative Commons licence will apply to this Work are set out in our licence referred to above.

Other than as permitted in any relevant BMJ Author's Self Archiving Policies, I confirm this Work has not been accepted for publication elsewhere, is not being considered for publication elsewhere and does not duplicate material already published. I confirm all authors consent to publication of this Work and authorise the granting of this licence.

For peer review only

Towards universal health coverage for people with stroke in South Africa: a scoping review

Sjan-Mari van Niekerk,[1] *Sureshkumar Kamalakannan ,[2,3] Gakeemah Inglis-Jassiem,[1] Maria Y Charumbira[1], Silke Fernandes,[4] Jayne Webster,[5] René English,[6] Quinette A Louw,[1] Tracey Smythe[3]

*Corresponding Author

Dr Sureshkumar Kamalakannan
International Center for Evidence in Disability,
London School of Hygiene and Tropical Medicine, London, UK
suresh.kumar@lshtm.ac.uk

1 Division of Physiotherapy, Department of Health and Rehabilitation Sciences, Stellenbosch University, Cape Town, South Africa

2 Public Health Foundation of India, Indian Institute of Public Health Hyderabad, SACDIR, India

3 International Center for Evidence in Disability, London School of Hygiene and Tropical Medicine, London, UK

4 Department of Global Health and Development, Faculty of Public Health and Policy, London School of Hygiene and Tropical Medicine, London, UK

5 Department of Disease Control, Faculty of Infectious and Tropical Diseases, London School of Tropical Health and Medicine, London, UK

6 Division of Health Systems and Public Health, Department of Global Health, Faculty of Medicine and Health Sciences, Stellenbosch University, Cape Town, South Africa

Total word count – 4566 without abstract, tables, figure, and references

Keywords – Stroke; Universal Health Coverage; Health System; South Africa; Scoping Review

For peer review only - http://bmjopen.bmj.com/site/about/guidelines.xhtml

ABSTRACT – word count 311

Objectives: To explore the opportunities and challenges within the health system to facilitate the achievement of Universal Health Coverage (UHC) for people with stroke (PWS) in South Africa (SA).

Setting: South Africa

Design: Scoping Review

Search Methods: We conducted a scoping review of opportunities and challenges to achieve UHC for PWS in SA. Global and Africa-specific databases and grey literature were searched in July 2020. We included studies of all designs that described the health care system for PWS. Two frameworks, the Health Systems Dynamics Framework and WHO Framework, were used to map data on governance and regulation, resources, service delivery, context, re-orientation of care, and community engagement. A narrative approach was used to synthesise results.

Results

Fifty-nine articles were included in the review. Over half (n=31, 52.5%) were conducted in Western Cape province and most (n=41, 69.4%) were conducted in urban areas. Studies evaluated a diverse range of health system categories and various outcomes. The most common reported component was service delivery (n=46, 77.9%), and only four studies (6.7%) evaluated governance and regulation. Service delivery factors for stroke care were frequently reported as poor and compounded by context-related limiting factors. Governance and regulations for stroke care in terms of government support, investment in policy, treatment guidelines, resource distribution, and commitment to evidence-based solutions were limited. Promising supporting factors included adequately equipped and staffed urban tertiary facilities, the emergence of Stroke units, prompt assessment by health professionals, positive staff attitudes and care, two clinical care guidelines, and educational and information resources being available.

Conclusion

This review fills a gap in the literature by providing the range of opportunities and challenges to achieve health for all PWS in SA. It highlights some health system areas that show

For peer review only - http://bmjopen.bmj.com/site/about/guidelines.xhtml

encouraging trends to improve service delivery including comprehensiveness, quality, and perceptions of care.

STRENGTHS AND LIMITATIONS OF THIS STUDY

- A comprehensive search strategy was developed, and the search was carried out in global, national, and continental-specific databases.
- The scoping review methodology included double data extraction and data review to synthesise the state of the evidence on the topic.
- The use of a combination of two frameworks, the Health Systems Dynamics and Integrated People-Centred Health Services contributed to rigorous evaluation.
- There was no limitation on study design or exclusion based on methodological appraisal for the inclusion of records.
- Comparison of studies was challenged by heterogeneity, especially regarding design and aim.

For peer review only

INTRODUCTION

Stroke is a leading cause of death and disability worldwide (1). In South Africa (SA) stroke is the second most common cause of death after HIV/AIDS and a significant cause of morbidity (2–5). It is estimated that 75 000 people experience a stroke each year in SA, contributing to 564 000 stroke-related disability-adjusted life-years (6). Furthermore, stroke incidence in rural areas of SA is increasing; an estimated 33,500 strokes occurred in these areas in 2011, contributing to half of the national stroke burden (7). However, these data are likely underestimated due to the absence of a national stroke database or registry and the paucity of studies that were undertaken in a few parts of the country.

Stroke is the leading cause of disability in adults in SA, placing strain on social and health services (8). Increased prevalence of heart disease, hypertension, diabetes mellitus, behavioural factors such as smoking, and structural factors such as unchecked industrialisation and urbanisation, contribute to this epidemiological transition of stroke in many low and middle-income countries (LMICs)(9), including SA (2). The SA government has committed to the World Health Organisation (WHO) vision of achieving equitable, evidence-based rehabilitation for all by 2030 (10). South Africa's Constitution guarantees every citizen to have access to health services (section 27 of the Bill of Rights). The SA health system comprises the public sector (the government managed) and the private sector. Public health services are divided into primary, secondary, and tertiary institutions managed by provincial Departments of Health, with the National Ministry of Health being responsible for policy development and coordination (11). Individuals can access either public or private health services, with access to private health dependant on an individual's ability to pay for services. The majority of South Africans (84%), access health services through government-run public clinics and hospitals (12). SA, stroke care, including rehabilitation, occurs across a range of settings, from tertiary hospitals to remote community primary healthcare facilities, and can be provided individually or in a group setting, at home, in a community environment, or a specialist centre (2). Whilst public health policy in SA ascribes to primary health care and a decentralised approach, many stroke care, and rehabilitation services remain centralised at district and specialist rehabilitation hospitals (13). It is not clear how many people access rehabilitation services following stroke, what this rehabilitation entails, and how effective this rehabilitation is (14). Therefore, achieving key global health targets and development goals will be challenging, including Universal Health Coverage (UHC) (15,16).

For peer review only - http://bmjopen.bmj.com/site/about/guidelines.xhtml

UHC is achieved when every person receives essential services, such as health promotion, prevention, treatment, rehabilitation (including assistive technology), and palliative care, according to their needs and without financial hardship (17). Accessible, responsive, and quality stroke care services within a strengthened local health system will contribute to UHC for people with stroke (PWS) in SA. The extent to which UHC is currently achieved for PWS in SA is unknown (18). We aimed to describe the health system-related factors that will facilitate UHC for PWS and the shortcomings that currently limit the implementation of UHC for stroke care in SA.

METHODS

A scoping review was conducted according to the five-step approach recommended by Levac et al.(19) as outlined in our published protocol (20): 1) identifying the research question, 2) identifying relevant studies, 3) selecting the studies, 4) charting the data, and 5) collating, summarising and reporting the results. The results are reported according to the Preferred Reporting Items for Systematic Reviews and Meta-Analyses extension for Scoping Reviews (PRISMA-ScR) guidelines (21).

Patient and public involvement

No patients and/or public were involved in the design, conduct, reporting, or dissemination plans of this research.

Analytical framework

This review was guided by an analytical framework adapted from the Health Systems Dynamics Framework (HSDF)(22) and WHO Framework on integrated people-centred health services (IPCHS)(23). Our analytical framework includes all the HSDF components and two components from the IPCHS: 1) Re-orientation of care and 2) Enabling environment, which is appropriate to the SA context and population (Figure 1). ‘Resources’ and ‘Enabling environment’ were combined and titled ‘Resources’ as the data items described under each were similar.

For peer review only - http://bmjopen.bmj.com/site/about/guidelines.xhtml

Figure 1: Components of the analytical framework that incorporates components from the Health Systems Dynamics Framework (19) and WHO Framework on integrated people-centred health services (20). Identifying the research question

To answer the question ‘what are the opportunities and challenges within the SA health system to facilitate achieving UHC for people with stroke?’ the review objectives were to:

1. describe the health system-related factors that support and guide achieving universal stroke care in SA
2. describe the health system-related factors that limit achieving universal stroke care in SA
3. identify driving factors with the potential to bring change required to achieve universal stroke care in SA

Identifying relevant studies

In line with the purpose of scoping reviews, our approach was broad, with emphasis on studies that investigated any aspect of the health care system regarding stroke care in SA.

Search Strategy

We conducted a comprehensive search, according to the methodology described in our published protocol (20) and an example of the search strategy is available as a supplementary file (S1). Grey literature was identified through the National Electronic Thesis and Dissertation portal, and websites of relevant government and service provider agencies. Field experts were contacted to identify additional relevant evidence regarding stroke care in SA. Saturation was the point at which no new records were found for inclusion.

Eligibility criteria

Full text, SA-based studies on stroke care of any design that addressed at least one framework component were included (20).

Evidence selection

Two reviewers (SvN, SK) independently screened the titles and abstracts of identified studies. A third reviewer (GIJ) checked the results for accuracy. Results of the initial screening were compared, and full-text records were obtained for articles deemed eligible by at least one reviewer. Two reviewers (SvN, SK) independently screened the full texts using the eligibility criteria. Any discrepancies were resolved by discussion with a third reviewer

For peer review only - http://bmjopen.bmj.com/site/about/guidelines.xhtml

(GIJ). Data were managed with Covidence (https://support.covidence.org/help) and Excel (version 365).

Data charting

The six framework components were divided between three reviewers (SvN; SK; MC) who extracted, collated, and summarised relevant data into a purpose-built Microsoft Excel database. We considered the six components using the descriptions as outlined in Supplementary file 2 (S2) and data on the following study components were extracted:

- General study information, including author and year of publication
- Study design, sampling, and recruitment methods
- Study settings and dates conducted
- Population characteristics
- Study measures
- Research outcomes related to the framework components

The three reviewers compared their results and reached a consensus on the organisation of extracted data. The final data and analysis were evaluated by a research team member (TS), to ensure that interpretations were credible and valid.

Data synthesis and analysis

We summarised the study characteristics and the study designs. We used a framework analysis approach to deductively analyse data of the included studies, which consisted of five key steps as described by Ritchie et al. (24). The framework in figure 1 was used as a dynamic tool to aid this synthesis and data was managed with Atlas. ti (version 8) and Microsoft Excel (version 365).

The final synthesis of themes was confirmed following a critical discussion between all the authors. We undertook a narrative synthesis of the findings, highlighting supporting and limiting factors to achieving UHC for PWS in SA. The range of opportunities and challenges to achieve health for all PWS in SA was synthesised and included in the framework diagram.

RESULTS

For peer review only - http://bmjopen.bmj.com/site/about/guidelines.xhtml

We identified a total of 4,133 records and screened the abstracts of 508. After reviewing 75 full-text records, we included a total of 59 full texts in our review. A PRISMA flow diagram summarised the study selection process (Figure 2).

Figure 2: PRISMA flow chart

Study characteristics

The majority (n=41, 69.4%) of studies were conducted in urban areas, and over half of them (n=31, 52.5%) were undertaken in the Western Cape province. No studies were found from four of the nine provinces in SA (Free State, Mpumalanga, Northern Cape, or the northwest Provinces). The most common study design was quantitative (n=22, 37,2%), followed by mixed methods (n=14, 23.7%) and qualitative (n=10, 16.9%). Eighteen (30%) studies were community-based whilst the remaining studies recruited participants from clinics (n=12, 20.3%) or hospitals (n=16, 27.1%). The most commonly reported framework component was Service Delivery and (n=46, 77.9%) and the least reported was Governance and Regulation (n=4, 6.7%). (Table 1).

Supplementary file 3 (S3) provides a detailed summary of included records and Supplementary file 4 (S4) provides information on components reported per included record.

Variable	Category	N (%)
Province	Western Cape	31 (52.5%)
	Gauteng	12 (20.3%)
	National	6 (10.1 %)
	Eastern Cape	4 (6.7%)
	KwaZulu-Natal	2 (3.3%)
	Limpopo	1 (1.6%)
	Limpopo and Gauteng	1 (1.6%)
	Free State	0 (0%)
	Mpumalanga	0 (0%)
	Northern Cape	0 (0%)
	North West	0 (0%)
	Undefined	2 (3.3%)
Area	Urban	41 (69.4%)
	Rural and Urban	3 (5.0%)
	Peri-Urban	3 (5.0%)
	Rural	2 (3.3%)
	Undefined	10 (16.9%)
Levels of care	Community	18 (30.5%)
	Hospital	16 (27.1%)
	Primary Healthcare (Clinics; Community Health Centres)	12 (20.3%)

For peer review only - http://bmjopen.bmj.com/site/about/guidelines.xhtml

	Rehabilitation centres	6 (10.1%)
	Undefined	7 (11.8%)
Study design	Quantitative	28 (47.4%)
	Mixed methods	14 (23.7%)
	Qualitative measures	10 (16.9%)
	Review	2 (3.3%)
	Editorial	3 (5.0%)
	Guideline	2 (3.3%)
Record description	Primary Literature(publications)	34 (57.6%)
	Grey Literature: Dissertations	25 (42.3%)
Included population	PWS	34 (57.6 %)
	Editorials and reviews	8 (13.5%)
	Caregiver	6 (10.1 %)
	PWS + Caregiver	5 (8.4 %)
	PWS + HCP	3 (5.0 %)
	HCP	1 (1.6 %)
	PWS + HCP+ Experts	1 (1.6 %)
	Traditional healers + Caregivers	1 (1.6 %)
	Policy makers	0 (0 %)

PWS = people with stroke; HCP = health care provider

Table 1: Characteristics of included records (N=59)

Twenty-one articles (35.5%) reported on a single framework component (Service Delivery: n=12; Community Engagement: n=4; Governance and Regulations=2; Context: n=2; Re-Organisation of Care: n= 1) and the majority of articles reported on a combination of two or more framework components (n=38, 64.4%). Twenty-four articles (40.6%) reported on a combination of two framework components (Context and Service Delivery: n =11; Resources and Service Delivery: n=5; Community Engagement and Service Delivery: n =4; Re-Organisation of Care and Service Delivery: n=2; Community Engagement and Re-Organisation of Care: n =2) and fourteen articles (23.7%) reported on three or more framework component combinations (Community Engagement, Context, Service Delivery: n=3; Context, Resources, Service Delivery: n= 2; Community Engagement, Resources, Service Delivery: n =2; Governance and Regulations, Resources, Service Delivery: n=1; Context, Governance, and Regulations, Resources n=1; Community Engagement, Context, Resources: n=1; Community Engagement, Context, Resources, Service Delivery: n=4).

Service Delivery

For peer review only - http://bmjopen.bmj.com/site/about/guidelines.xhtml

Comprehensiveness

A comprehensive multi-disciplinary team (MDT), defined as consisting of five or more different types of health care professionals working together in a coordinated manner, was reported in nine studies (25-33). Two studies indicated that MDTs were either absent, limited, or inefficient (34,35).

Continuity of Care

Continuity of care was limited by poorly defined referral pathways, bed capacity for inpatient care, coordination of care, communication (among healthcare providers and with patients) in regards to care and discharge planning as well as follow-up systems. One study indicated that poor understanding of faith-based medicine by medical professionals and reciprocal lack of trust between medical and faith-based medicine practitioners may hinder adequate stroke care (36). At the community level, referral to support groups lacked coordination and stroke survivors lacked knowledge of care options (29,37–39). Two studies conducted in a rural part of the Western Cape reported that 30% (n=19) of the 64 patients who were referred for home-based care, did not receive rehabilitation care from community health workers following an assessment and subsequent treatment plan designed by a district therapist. The lack of therapy sessions was due to a long waiting time for appointments. Those who did receive therapy had a median of three visits which lasted 20 minutes each (2,40). Waiting time for investigations such as magnetic resonance imaging or computerised tomography scans and general stroke care was lengthy (34,38,41). Findings included delays in investigations being associated with a significant increase in length of stay (42) and doctor-led models, where a doctor is solely responsible for the patient's care and flow of information, leading to delays in investigations and/or treatments (40,43,44).

Timeliness of Care

Bed shortages (30,35,38,41,45) resulting in the pressure to discharge patients in hospitals precluded rehabilitation and delayed post-discharge rehabilitation (31,35,46,47). In addition, doctor-led models of care were reported to lead to delays of care as staff wait for instruction or referral from a doctor before conducting investigations or administering treatment (31,37). Four studies (31,46–48) reported that patients were discharged when medically stable (average stay was 5-10 days at secondary or tertiary hospitals (30,39,46,49)) despite functional deficits (29,30,46,50,51). Cunningham (2012) (47) reviewed 168 stroke patient

For peer review only - http://bmjopen.bmj.com/site/about/guidelines.xhtml

acute care records from the Eastern Cape province and found only 15% were referred for physiotherapy on the day of or a day before discharge from in-patient acute care (47). Over weekends, 13% of acute-care patients did not receive any therapy (47). Difficulty with securing follow-up appointments and cancellations influenced the timeliness of post-discharge care (2,34,40,52,53).

Quality of Care

Four studies conducted in the Western Cape found that patients received between one and five rehabilitation sessions during acute care in hospital, except for the specialised sub-acute, in-patient Rehabilitation Centre where patients typically received 17 sessions (28,31,54-55), LOS was typically 5 - 10 days and approximately 30 days in rehabilitation facilities (42,56–59). One study reported that prompt assessment by rehabilitation professionals was associated with a shorter length of stay (42).

Perceptions of Care

Conflicting evidence exists in regards to perceptions of care with ten studies reporting positive staff attitudes (32,34,40,44,59–64) while nine studies reported negative staff behaviour and attitudes (33,36,40,52,60,65–68). A further four studies found that PWS were dissatisfied with the healthcare service along the entire continuum of care, which was driven by a lack of information about their treatment and further referral (34, 36,68). Leichtfuss (2009) (33) highlighted the significant discrepancy (p-value = 0.00438) between doctors' understanding and patients' perception of the effectiveness of the doctors' communication; 80% (n=28) of doctors compared to 50% (n=24) of patients thought that sufficient information was communicated (33). The study also found that patients perceived nursing services as inefficient and inadequate, which was supported by doctors who expressed the need for nursing staff who were trained in stroke care (33). Caregiver support and training were lacking (39,65,67,69) and resulted in caregiver burnout (66). Caregivers indicated the need for additional training and help, particularly with toileting and bath transfers, and requested more home visits by therapists (39). Table 2 outlines measures and study findings that target Service Delivery.

Table 2: Supportive and limiting factors influencing different components of Service Delivery (N=46).

	**Service Delivery**	**Source of evidence: Author (year)**

**Comprehensiveness of Care**		
Facilitators	Comprehensive multi-disciplinary teams consisting of five or more different healthcare professionals in Western Cape province	Groenewald and Rhoda (2017); Rhoda et al. (2015); Joseph (2012); Rouillard et al., (2012); Leichtfuss (2009) Ras (2009); Wasserman et al, (2009); Rhoda (2009); De la Cornillère (2007)
Barriers	Limited/absent multi-disciplinary team consisting of less than five different healthcare professionals	Cawood (2012); De Villiers (2011)
**Continuity of Care***		
Barriers	Poor referral pathways (community; hospital)	Masuku (2018); Mandizvidza (2017); Cawood & Visagie (2016); Joseph (2012); De la Cornillère (2007); Kleinheibst (2007)
	Poor follow-up and referral post-discharge	Rhoda (2014); Rouillard (2012); Bham & Ross (2005); Scheffler and Mash (2019);
	Lack of reciprocal respect and understanding and coordination between traditional and medical healthcare professionals	Bham & Ross (2005)
**Timeliness of Care***		
Barriers	Long queues in hospitals, community health clinics, and outpatient clinics	Cawood (2012); Mudzi (2013)
	Long waiting times for follow-up appointments	Arowoiya (2014)
	Long waiting times for inpatients to receive specialised health services	Matshikiza (2019); Mandizvidza (2017); Parekh & Rhoda (2013); Cawood (2012); Bryer (2009)
	Doctor-centric model of care	Cawood & Visagie (2015); Cawood (2012)
	Poor collaboration between health care providers	Cawood (2012); Parekh (2011)
	Inadequate rehabilitation during hospital stays	Cunningham (2012); Hilton (2011); De Villiers (2009); Rhoda (2009)
**Quality of Care**		
Facilitators	Prompt assessment by an allied health professional significantly decreases the length of stay	Viljoen (2014)
Barriers	Lack of appropriate care due to lack of stroke-specific knowledge	Mandizvidza (2017); Leichfust (2009); Ras (2009)
	A low number of in-hospital rehabilitation sessions	Groenewald & Rhoda (2017); Parekh (2011) ; Rhoda et al (2011)

For peer review only - http://bmjopen.bmj.com/site/about/guidelines.xhtml

		; Rhoda (2009)
	Inadequate length of stay at all levels of care except for specialist rehabilitation facilities	Groenewald (2018); Mabunda (2015); Rhoda (2014); Viljoen (2014); Hilton (2011); Parekh (2011); Blackwell & Littlejohn (2010); Mudzi (2010); Ras (2009); Kleinhebst (2007); Felemengas (2004)
**Perceptions of Care**		
Facilitators	Positive staff attitudes and care	Taylor & Ntusi (2019); Groenewald (2018); Kotsokoane (2018); Hossain (2016); Kusambiza-Kiingi (2016); Cawood & Visagie (2015); Bham & Ross (2005); Cawood (2012); Ntamo (2011); De la Cornillère (2007)
Barriers	Negative staff attitudes and behaviour e.g., impersonal care; poor support; poor communication; lack of cultural sensitivity, rudeness, and delayed assistance with patient's personal hygiene	Smith (2019); Cawood & Visagie (2015); Makganye (2015); Posner (2015); Arowoiya (2014); Leichtfuss (2009); Thomas & Greenop (2008); Bham & Ross (2005); Biggs (2005)
	Dissatisfaction with health care received	Arowoiya (2014) ; Cawood (2012); Bham & Ross (2005) Ntamo (2011); Kleineibst (2007)
	Lack of caregiver training	Kusambiza-Kiingi (2017); Mashau et (2016); Mudzi (2010); Kleineibst (2007); Rouilliard (2012); Felemengas (2004)

*No supporting factors reported.

Resources

Infrastructure

A mixed-method study by Ntamo et al.(63) reported that substantial traveling distances were required to access rural healthcare facilities(63), this was echoed in Bryer's editorial on the need for community-based stroke care (45). Makganye et al.(60) reported that 71% of 85 rural patients (n = 60) lived over 25 km away from their nearest hospital (60). Physical access for people with a disability was further limited by poor building infrastructure (e.g. no ramps, vast distances between departments) or the surrounding uneven terrain (70).

Three records (longitudinal study, cross-sectional study, and editorial) reported a lack of diagnostic equipment in rural facilities (26,38,45), in contrast with well-resourced urban

For peer review only - http://bmjopen.bmj.com/site/about/guidelines.xhtml

rehabilitation centres (30,31) which often remained inaccessible (30,31,45) due to the location of these facilities requiring long traveling distances. A mixed-methods study reported frequent stock-outs of basic medication at the primary care level, which resulted in additional expenses and patients' reluctance to return to rural clinics (63), and these findings were echoed in an editorial (64).

Human Resources

Adequately equipped urban rehabilitation centres were described in two studies (30; 31). Six studies reported high bed demand and rehabilitation workforce shortages led to high healthcare provider workloads (30,31,34,38,45,60). Therapists reportedly treated 2-3 times more patients than the daily recommendation (30). Mandizvidza (2017) (38) reported that nursing shortage at all healthcare levels in rural KwaZulu Natal negatively impacted basic stroke care. Better-resourced urban tertiary hospitals in the Western Cape were also reported to experience staff shortages (38). A quantitative cross-sectional study reported that rehabilitation services are severely limited at the primary care level with half of the community health centers in the Western Cape providing rehabilitation services, and only two offering speech therapy (31). Stroke care was often provided by healthcare professionals without specific stroke-related training (30,33,38) (Table 3). There were no articles that reported on the financial allocations in place as a resource for stroke care

Table 3. Facilitators and barriers influencing different components of Resources (n = 16)

	Resources	Source of evidence: Author (year)
**Infrastructure**		
Facilitators	Adequate equipment (urban rehabilitation centre setting)	Ras (2009); Rhoda et al (2009)
Barriers	Lack of equipment (rural setting)	Mandizvidza (2017); Cawood (2012); Cunningham (2012); Rhoda et al (2009)
	Inadequate number of ambulances; ineffective systems to request an ambulance	Mandizvidza (2017); Biggs (2005)
	Poor accessibility of health centres due to location, building structure, or terrain	Maleka (2012); Ntamo (2011); Bryer (2009); Rhoda (2009)

For peer review only - http://bmjopen.bmj.com/site/about/guidelines.xhtml

	surrounding the health facility	
	Insufficient number of beds or hospitals due to fiscal problems	Matshikiza (2019); Mandizvidza (2017); De Villiers (2011); Bryer (2009); Ras (2009)
	Inadequate special investigation infrastructure for diagnosis and management	Mandizvidza (2017); Villjoen (2016); Bryer (2009)
	Frequent medication outages	Taylor & Ntusi (2019); Ntamo (2011)
**Human Resources** *		
Barriers	Staff shortages	Mandizvidza (2017); Makganye (2015) ; Cawood (2012); Bryer (2009), Ras (2009); Connor (2005)
	Lack of stroke-specific training for staff	Mandizvidza (2017); Leichfust (2009); Ras (2009); Kleineibst (2007)

*no supporting factors reported

Context

Wellbeing and caregiver factors

Two longitudinal and one retrospective surveys reported mental health problems such as anxiety and depression among PWS and caregivers (26,27,71). PWS also related feelings of confinement, personality changes, imposed family adjustments, and caregiving burden (50,57,72). Gender bias in caregiving roles was reported where women commonly left employment to assume caregiving responsibilities of male partners or parents (46) or children cared for women with stroke (47,57,70).

Financial implications

The financial burden increased when spouses became primary caregivers (without gainful employment) or through the employment of additional caregivers (57). The financial burden post-stroke was high due to additional caregiving costs (60,73) and limited access to disability-, old age- or child-support grants (52,65). The financial burden among rural stroke survivors was compounded by low income before the stroke, difficulty in obtaining social grants due to limited awareness of eligibility criteria and the application processes, and lack of transport to submit grant applications (53,66). Poverty impacted access or utilization of

For peer review only - http://bmjopen.bmj.com/site/about/guidelines.xhtml

rehabilitation as available finances were preferentially used to meet basic needs such as food (74).

Access to Transport

Six studies reported transport being a limiting factor to access care due to expensive private transport, unreliable public transport, and inflated costs of a trip to accommodate assistive devices (32,37,39,63,65,75).

Cultural beliefs and Health Literacy

Two qualitative case studies reported that PWS in SA held cultural beliefs regarding the cause and recovery of strokes, such as ascribing stroke to witchcraft or religious beliefs (36, 60). Poor health literacy (60,66,68) and these beliefs further affected the care-seeking ability of communities. Bham and Ross (2005) (36) reported that healthcare professionals needed greater awareness of cultural practices such as the inclusion of extended family in decision-making procedures, adaption of communication style when interviewing older persons, and sensitivity to religious and traditional beliefs, to facilitate the inclusion and full participation of marginalised communities(36).

Community Engagement

Self-efficacy

Leichtfuss (2009) (33) found that PWS and/or their caregivers believed that they were not involved in care decision-making. Felemengas (2004) (57) and Cawood (2012) (34) reported that PWS were neither confident with self-health management nor satisfied with pre-discharge training and information (34,57). A large mixed methods study (65) that included a survey (N= 418) reported that PWS and caregivers lacked awareness of the availability and benefit of rehabilitation services or support groups and this was echoed by Burton's editorial (76). Cawood et al (43) found that nearly half (n=53; 47%) of the participants in their cross-sectional study indicated via a survey that they did not receive assistance from stroke organisations (40). Low participation in a peer support program was found (29) despite patients who attended stroke support reporting better self-efficacy and feeling supported (34,65).

For peer review only - http://bmjopen.bmj.com/site/about/guidelines.xhtml

Community Integration

People with stroke were not fully re-integrated into their communities (61,77) due to negative attitudes of family, friends, and society (34). Inaccessible community activities (28.3%), poor mental health (18.9%)(78), financial constraints (45.3%)(77), and inaccessible transport (65) contributed to limited community integration. Fear of stigmatisation (70), functional dependency especially due to incontinence (32,37,50,63,79), and fear of becoming a vulnerable victim of crime (40) also heightened limiting factors to integration.

Homecare resources

A Stroke Home Care booklet (in different languages) was developed for the SA context (80). In focus group discussions, seven-stroke survivors (n=15; 46%) demonstrated improved knowledge, confidence, and ability to communicate information about their stroke after using the booklet (80). However, the sample included in this study was small and the booklet was only available in English when acceptability was tested, and the findings are therefore not generalizable. The stories and pictures were found to be culturally sensitive (80).

Reorganisation of Care

Educational and information resources

This review found two educational resources available via institutional websites for the public: The stroke Home Care booklet (80) and the SA contextualised Bridges Stroke Self-management intervention workbook (59). The MyStroke website (www.mystroke.co.za) was developed following a public health awareness campaign and lists available stroke care centres and services for better coordination (76). The mySOS app is an e-health initiative that directs and connects users with emergency care, potentially improving the timeliness of care. In rural settings, telemedicine was used to connect with specialist services (81). However, none of these resources include trials of efficacy or determined the usage of the website or application.

Stroke Unit

At a central hospital in Western Cape, the stroke unit was associated with reduced mortality and increased rehabilitation referral, staff training, and family involvement in treatment decisions (48). Stroke units were recommended in evidence-based SA stroke care guidelines (13,82).

Palliative care integration

For peer review only

Findings based on focus groups of patients showed that palliative care should be incorporated into stroke care. However, better education of all stakeholders on palliative care benefits was needed (44).

Governance and Regulation

Two-stroke clinical care guidelines for SA were identified (13,82). One focussed on acute and post-acute stroke care (82), and the other on stroke rehabilitation (13). Mandizvizda (2017) (38) evaluated the level of adherence to the acute stroke care guideline in all levels of care in the Western Cape province and reported poor adherence in primary, secondary, and tertiary hospitals (general wards), with the two Stroke Units (situated in tertiary hospitals), being the most compliant (38). Challenges to adherence included staff shortages, limited access to diagnostic investigations, and delays in patients presenting to healthcare facilities (38).

There were no national stroke-specific policies. Whilst many people with disabilities are reliant on financial support from the government using grants, there was no specific policy on financial support for PWS or their caregivers. Poor intersectoral coordination between government departments was reported about responsibilities for policies concerning persons with disabilities (83). Governance and Regulations were the most limited component with limited leadership and policy on how stroke care should be implemented and conducted at all levels of care.

Limiting and supporting factors

Health system limitations and factors that support the achievement of UHC for PWS in SA are presented in Figure 3. Findings of each health system component of the framework are mapped and identify challenges and opportunities that speak to stroke care in the public sector.

Figure 3: Limiting and supporting factors toward achieving UHC

DISCUSSION

This scoping review summarises the available evidence of achieving health for all PWS in SA. Studies evaluated a diverse range of health system categories and various outcomes, with the majority of studies reporting on two or more framework components. Key health system limiting factors were a lack of governmental regulation in terms of stroke policies and

For peer review only - http://bmjopen.bmj.com/site/about/guidelines.xhtml

guidelines; poor timeliness of care; a lack of the continuity of care and a lack of a comprehensive multi-disciplinary team at rural health facilities; bed and staff shortages and a lack of stroke-specific training; poor access to acute care and diagnostic equipment; regular medication outages; lack of caregiver training and contradictory reports on perceptions of care. Promising facilitating factors were adequately equipped and staffed urban tertiary facilities, the emergence of Stroke Units in urban areas, prompt assessment by rehabilitation professionals, positive staff attitudes and care, two clinical care guidelines, and educational and information resources being available. Drivers to achieve UHC for PWS in SA may include better governance and regulation to mitigate fiscal shortfall resulting in infrastructure and human resource limiting factors, the intersectoral collaboration between government departments to assist with access to social support, and reliable, affordable transport to access healthcare.

A key finding of this review is a lack of adequate Governance and Regulations in terms of government support and investment in policy and treatment guidelines, resource distribution, and commitment to evidence-based solutions (e.g. stroke units). Equity for people with disabilities, including PWS, requires a concerted commitment from the SA government to ensure that UHC for all is achieved (84). Opportunities to facilitate these renewed efforts include administrative interventions by both government and hospital management to address system-based limiting factors, such as access to patients' medical records and obtaining appointments. Addressing staff shortages and improving stroke-specific training may mitigate the excessive workload of healthcare workers and improve service delivery, as was achieved during a pilot program in Namibia, where an increase in the number of nurses resulted in improved service delivery (85). However, attracting and retaining health professionals in rural and remote areas is multi-factorial (86,87) and contextual strategies to attract and retain health professionals in these areas are needed (86). Dedicated stroke units in hospitals have reduced stroke mortality, increased access to rehabilitation from multidisciplinary teams, and have resulted in improved discharge planning services at these stroke units compared to managing PWS in general medical wards (48). Support for these units may contribute to better stroke outcomes and improve community re-integration and return to work for PWS in SA.

Service Delivery and Context related factors were most frequently reported in combination (n =11, 18.6%) and were consistently reported as poor. These findings of poor timeliness of care, a lack of continuity of care, and a lack of a comprehensive multidisciplinary team in

For peer review only - http://bmjopen.bmj.com/site/about/guidelines.xhtml

rural areas, are similar to health system weaknesses found in Rwanda and Malawi (88). The main hindrances affecting service delivery in SA were poor referral networks within and between healthcare facilities (29,32,38,39,43,74), inadequate caregiver training (26,39,50,57,72), fiscal inadequacies resulting in inadequate staffing levels at all levels of care (30,34,38,45,60,89), lack of stroke-specific staff training (30,33,38,39), bed shortages (35,38,41,45), a lack of acute care and diagnostic equipment at primary and district health facilities (38,42,45), and a lack of availability of well-equipped rural rehabilitation facilities (31,34,38,47). As a result, many PWS are lost to follow-up care leading to poor management of comorbidities and potentially placing patients at risk of recurrence and secondary complications such as spasticity, pressure sores, aspiration pneumonia, and mobility difficulties (90).

Access to equitable and affordable health care for PWS may be affected by contextual factors outside the healthcare system. Social determinants of health (poverty, education) and general safety can be addressed through intersectoral collaboration. Social service and health sector collaboration may ensure that eligible PWS are aware and have access to social grant support. This was echoed as an international need in a scoping review which included studies from North America, the United Kingdom, and Europe (88). Cooperation between both private and public transport services, and the health sector is needed to find a solution for accessible, affordable, and reliable transport for PWS and their caregivers. Whilst there is strong evidence of the link between lack of access to transport and negative effects on health care, research on possible solutions and the effectiveness thereof is scarce.

Reported Supporting Factors

Despite the many limiting factors that were described, supporting factors to achieve UHC for PWS in SA were also reported. Supporting factors to UHC that are already in place include well-equipped rehabilitation facilities in urban areas (N=2), a stroke unit in an urban area (N=1), perception of positive staff attitude (N=10), and comprehensive multi-disciplinary teams in urban, tertiary hospitals (N=9). There were also two clinical care guidelines and educational and information resources were available. Although some PWS reported their dissatisfaction with the care they have received (34,36,39,52,63) several studies reported patient and caregiver satisfaction, as well as positive staff attitudes, which were perceived to facilitate physical improvement through rehabilitation compliance(28,29,32,34,40,44,60–62,64). This was consistent with findings where the attitude and emotional approach of health

For peer review only - http://bmjopen.bmj.com/site/about/guidelines.xhtml

For peer review only

professionals, as well as caregivers, affected the level of motivation for rehabilitation attendance in PWS (91). Maclean et al.(91) found that a positive rapport between patients and healthcare providers resulted in increased motivation and easy transmission of information about rehabilitation (91).

Implications for future research

The limited supporting factors and a multitude of limiting factors reported in the included studies highlight the gaps that remain and present opportunities for future research. Key questions include the effect of continuity of care, timeliness of care, and perceptions of care on the improvement of service delivery, as well as the effect of resources (such as staffing, bed allocation, and access to diagnostic equipment), and the impact of stroke-related training on service delivery. The distribution of research as reported in this review was found to be disproportionate with just over half of the studies being conducted in a single province (Western Cape) and largely in urban areas, with four of the nine provinces not being reported on at all. Insights regarding barriers and facilitators to UHC for PWS residing in these unreported provinces are warranted.

Future research may focus on:

- Strategies to coordinate care for multi-morbidity (e.g., combined appointments with different health professionals) to minimise financial hardship on healthcare users and to evaluate effective and efficient holistic management of health, compared to silo treatment approaches.
- Extension of research on stroke services in the under-reported provinces.
- Evaluation of accessible, quality services beyond urban areas.
- Development and testing of stroke-specific capacity development for staff that is evidence-based, patient-centered, and holistic. Factors to highlight in training may include cultural responsiveness and awareness of the social determinants of health.
- Strategies to improve and implement person-centred discharge planning, which should include caregiver training and support before and after discharge.
- Development and evaluation of sustainable strategies to provide peer support groups either in person or on a digital platform, for both PWS and their caregivers, to provide ongoing support.

For peer review only - http://bmjopen.bmj.com/site/about/guidelines.xhtml

- Innovative public health campaigns via social media, television, or radio to increase the awareness of stroke signs and the urgency of seeking help. The impact, reach, and process evaluation of such campaigns should monitor effectiveness.

Strengths and limitations of the scoping review

We used a comprehensive search strategy that followed PRISMA guidelines, and robust methods that included double data extraction and review to produce a comprehensive state of the evidence. Our framework for analysis included a people-centered framework that acknowledged that health service provision should be coordinated around people's needs and preferences and provided in a way that is safe, effective, timely, affordable, and of acceptable quality. The framework also acknowledged the political context and the social and economic determinants of health. However, this review has several limitations. There was no restriction on study design for the inclusion of articles through methodological appraisal and the variety in study design and aim made a comparison of study results difficult. This lack of rigour in the included studies as well as the disproportioned distribution of where research on stroke care services was conducted may have led to non-generalisable conclusions. We included research articles, dissertations, and commentaries, and there may be evidence missed from health or government websites.

CONCLUSION

Stroke is the leading cause of disability in adults in SA, which places strain on national social and healthcare services. Although the SA government has committed to the WHO vision of achieving equitable, evidence-based rehabilitation for all by 2030, this review highlights the multifactorial nature of the health system in SA that requires strengthening and indicates the lack of readiness for UHC for PWS, especially on adequate governance and regulations.

Despite the available guidance on the best strategies to support healthcare systems in delivering stroke care services, the main findings of this review show that the stroke care services for PWS in SA are generally limited with a strong urban bias. The findings of this review have highlighted health systems challenges that speak to inequitable stroke care in the public sector. Health system strengthening driven by good governance & regulation of health

For peer review only - http://bmjopen.bmj.com/site/about/guidelines.xhtml

services, continuity of care, timeliness of care, accessibility to facilities, acute stroke care, and diagnoses, and well-equipped rehabilitation services is urgently needed. Health system limitations are compounded by contextual factors, highlighting the need for health system strengthening strategies that are tailored for the local context.

This scoping review highlights some health system areas that show encouraging trends to improve service delivery including comprehensiveness, quality, and perceptions of care. The results of this review can be used to inform policymakers and healthcare professionals of healthcare system challenges and opportunities to effectively move towards UHC for PWS in SA. Governments should be held more accountable for stroke care in terms of financial resource allocation and prioritize and include this marginalised group in the proposed national health insurance scheme.

DECLARATIONS

Ethics and dissemination Ethical approval were not required for this scoping review, as it only included published and publicly available data. The findings of this review are published in an open-access, peer-reviewed journal and developed an accessible summary of the results for website posting and stakeholder meetings.

Contributors SvN and SK in consultation with all authors constructed the search. SvN, SK, and MC extracted all data in consultation with all authors. SvN, SK, GI-J, JW, QAL, and TS analysed the extracted data. SvN drafted and revised the paper. SK, GI-J, MC, SF, RE, JW, QAL, and TS reviewed the manuscript and provided feedback on the drafts. All authors read and approved the final manuscript.

Competing interests None declared.

Funding This research was commissioned by the National Institute for Health Research (NIHR) Global Health Policy and Systems Research Development Award using UK aid from the UK government. Grant number NIHR130180.

Data sharing statement No Additional data available

Data availability Statement All data relevant to the study are included in the article or uploaded as supplementary file

Disclaimer The views expressed in this publication are those of the author(s) and not necessarily those of the NIHR or the Department of Health and Social Care.

For peer review only - http://bmjopen.bmj.com/site/about/guidelines.xhtml

Patient consent for publication Not required.

For peer review only

For peer review only - http://bmjopen.bmj.com/site/about/guidelines.xhtml

REFERENCES

Bibliography

1. Naghavi M, Abajobir AA, Abbafati C, Abbas KM, Abd-Allah F, Abera SF, et al. Global, regional, and national age-sex specifc mortality for 264 causes of death, 1980-2016: A systematic analysis for the Global Burden of Disease Study 2016. Lancet. 2017

2. Scheffler E, Mash R. Surviving a stroke in South Africa: outcomes of home-based care in a low-resource rural setting. Top Stroke Rehabil. 2019 Aug 18;26(6):423–34.

3. Pillay-van Wyk V, Msemburi W, Laubscher R, Dorrington RE, Groenewald P, Glass T, et al. Mortality trends and differentials in South Africa from 1997 to 2012: second National Burden of Disease Study. Lancet Glob Heal. 2016

4. Maredza M, Bertram MY, Gómez-Olivé XF, Tollman SM. Burden of stroke attributable to selected lifestyle risk factors in rural South Africa. BMC Public Health [Internet]. 2016 Jan 1;16:143. Available from: http://search.ebscohost.com/login.aspx?direct=true&db=awn&AN=26869067&site=ehost-live&scope=site

5. Coovadia H, Jewkes R, Barron P, Sanders D, McIntyre D. The health and health system of South Africa: historical roots of current public health challenges. The Lancet. 2009.

6. Bertram MY, Katzenellenbogen J, Vos T, Bradshaw D, Hofman KJ. The disability adjusted life years due to stroke in South Africa in 2008 [Internet]. Vol. 8 Suppl A1, International journal of stroke: official journal of the International Stroke Society. 2013. p. 76–80. Available from: http://search.ebscohost.com/login.aspx?direct=true&db=awn&AN=23295022&site=ehost-live&scope=site

7. Maredza M, Bertram MY, Tollman SM. Disease burden of stroke in rural South Africa: An estimate of incidence, mortality and disability adjusted life years. BMC Neurol. 2015 Apr 12;15(1).

8. Eksteen G, Mungal-Singh V. Salt intake in South Africa: A current perspective. Journal of Endocrinology, Metabolism and Diabetes of South Africa. 2015.

9. Kabudula CW, Houle B, Collinson MA, Kahn K, Gómez-Olivé FX, Clark SJ, et al. Progression of the epidemiological transition in a rural South African setting: findings from population surveillance in Agincourt, 1993-2013. BMC Public Health. 2017 May 10;17(1).

10. WHO, The World Bank. Tracking Universal Health Coverage: 2017 Global Monitoring Report. World Health Organisation. 2017.

11. Mahlathi, P. and Dlamini J. Minimum data sets for human resources for health and the

For peer review only - http://bmjopen.bmj.com/site/about/guidelines.xhtml

surgical workforce in South Africa's health system: a rapid analysis of stock and migration. African Inst Heal Leadersh Dev. 2015

12. Naidoo S. The South African National Health Insurance: a revolution in health care delivery! J Public Health (Bangkok). 2012;34(1):149–150.

13. South African Contextualised stroke rehabilitation guideline (SACSRG), 2019. 2019;

14. Vallabhjee K D V. Western Cape Government. Healthcare 2030: the road to wellness. Cape Town: Western Cape Department of Health. 2014.

15. Kuper H, Hanefeld J. Debate: can we achieve universal health coverage without a focus on disability? BMC Health Serv Res. 2018

16. Hashemi G, Kuper H, Wickenden M. SDGs, Inclusive Health and the path to Universal Health Coverage. Disabil Glob South. 2017

17. Evans DB, Etienne C. Health systems financing and the path to universal coverage. Bulletin of the World Health Organization. 2010.

18. Louw Q, Twizeyemariya A, Grimmer K, Leibbrandt D. Estimating the costs and benefits of stroke rehabilitation in South Africa. J Eval Clin Pract. 2020 Aug 1;26(4):1181–7.

19. Levac D, Colquhoun H, O'Brien KK. Scoping studies: Advancing the methodology. Implement Sci. 2010 Sep 20;5(1).

20. van Niekerk S-M, Inglis-Jassiem G, Kamalakannan S, Fernandes S, Webster J, English R, et al. Achieving universal health coverage for people with stroke in South Africa: protocol for a scoping review. BMJ Open. 2020 Oct;10(10):e041221.

21. Tricco AC, Lillie E, Zarin W, O'Brien KK, Colquhoun H, Levac D, et al. PRISMA extension for scoping reviews (PRISMA-ScR): Checklist and explanation. Annals of Internal Medicine. 2018.

22. Olmen J Van, Criel B, Bhojani U, Marchal B, Belle S Van, Chenge MF, et al. The Health System Dynamics Framework: The introduction of an analytical model for health system analysis and its application to two case-studies. Heal Cult Soc. 2012;2(1):1–21.

23. World Health Organization (WHO). Framework on integrated, people-centred health services: Report by the Secretariat. World Heal Assem. 2016

24. Ritchie J, Lewis J, Nicholls C, Ormston R. Qualitative research practice: A guide for social science students and researchers [Internet]. 2013 [cited 2021 Jan 10]. Available from: https://books.google.co.za/books?hl=en&lr=&id=EQSIAwAAQBAJ&oi=fnd&pg=PP1&dq=Ritchie+J,+Lewis+J,+Nicholls+C,+Ormston+R&ots=l-QMhvTv3P&sig=Hz7vuHbw4WR5vGMtiWPszn_KSy4

For peer review only - http://bmjopen.bmj.com/site/about/guidelines.xhtml

25. Wasserman D. Community-based care of stroke patients in a rural African setting. South African Med J [Internet]. 2009 Jan 1;99(8):13. Available from: http://search.ebscohost.com/login.aspx?direct=true&db=awn&AN=803237&site=ehost-live&scope=site

26. Rouillard S, De Weerdt W, De Wit L, Jelsma J. Functioning at 6 months post stroke following discharge from inpatient rehabilitation. South African Med J. 2012;102(6):545–8.

27. Rhoda A, Cunningham N, Azaria S, Urimubenshi G. Provision of inpatient rehabilitation and challenges experienced with participation post discharge: quantitative and qualitative inquiry of African stroke patients. BMC Health Serv Res [Internet]. 2015 Jan 1;15:423. Available from: http://search.ebscohost.com/login.aspx?direct=true&db=awn&AN=26412081&site=ehost-live&scope=site

28. Groenewald R, Rhoda AJ. Multidisciplinary rehabilitation outcomes of stroke patients in the Western Cape of South Africa [Internet]. Vol. 23, African Journal for Physical, Health Education, Recreation and Dance. 2017. p. 267–76. Available from: http://search.ebscohost.com/login.aspx?direct=true&db=awn&AN=ajpherd-164230&site=ehost-live&scope=site

29. Joseph C. Determining the process of rehabilitation and the outcomes of patients at a specialised in-patient centre in the Western Cape. 2011;2–216. Available from: http://etd.uwc.ac.za/xmlui/handle/11394/2973

30. Ras T. An audit of geriatric stroke rehabilitation services at a post-acute hospital (Booth Memorial) in urban Cape Town, South Africa. Diss Univ Cape T. 2009;

31. Rhoda A, Mpofu R, DeWeerdt W. The rehabilitation of stroke patients at community health centres in the Western Cape. South African J Physiother. 2009;65(3).

32. De la Cornillère W. Participants' experience of the Bishop Lavis Rehabilitation Centre stroke group [Internet]. 2007. Available from: http://search.ebscohost.com/login.aspx?direct=true&db=awn&AN=NX0185175&site=ehost-live&scope=site

33. Leichtfuss U. Aspects of stroke rehabilitation in private acute-care hospitals. Master Philos (mphil) Fac Heal Sci. 2009

34. Cawood J. Rehabilitation outcomes of uninsured stroke survivors in the Helderberg Basin [Internet]. 2012. Available from: http://search.ebscohost.com/login.aspx?direct=true&db=awn&AN=NX0217654&site=ehost-live&scope=site

35. de Villiers L, Badri M, Ferreira M, Bryer A. Stroke outcomes in a socio-economically disadvantaged urban community. S Afr Med J. 2011 May;101(5):345–8.

For peer review only

36. Bham Z, Ross E. Traditional and western medicine: cultural beliefs and practices of South African Indian Muslims with regard to stroke [Internet]. Vol. 15, Ethnicity & Disease. 2005. p. 548–54. Available from: http://search.ebscohost.com/login.aspx?direct=true&db=awn&AN=16259475&site=ehost-live&scope=site

37. Cawood J, Visagie S. Stroke management and functional outcomes of stroke survivors in an urban Western Cape Province setting. South African J Occup Ther. 2016;46(3):21–6.

38. Mandizvidza V. Quality of current ischaemic stroke care practices in the Cape Metro Health District, South Africa. Diss Univ Cape T. 2017.

39. Kleineibst LJ. The effectiveness of a caregiver support programme to address the needs of primary caregivers of stroke patients in a low socio-economic community [Internet]. 2007. Available from: http://search.ebscohost.com/login.aspx?direct=true&db=awn&AN=NX0185273&site=ehost-live&scope=site

40. Cawood J, Visagie S. Environmental factors influencing participation of stroke survivors in a Western Cape setting. African J Disabil. 2015;4(1):1–9

41. Matshikiza. Barriers to acute stroke care at a tertiary Hospital in the Western Cape. 2019

42. Viljoen C. Audit of the quality and cost of acute inpatient stroke care in the general medical wards at Groote Schuur Hospital. 2014

43. Cawood J, Visagie S, Mji G. Impact of post-stroke impairments on activities and participation as experienced by stroke survivors in a Western Cape setting. South African J Occup Ther. 2016;46(2):10–5.

44. Hossain M. To investigate the Need for Palliative care in Cerebrovascular Accident (stroke) patients at Ladysmith Regional Hospital. 2014

45. Bryer A. The need for a community-based model for stroke care in South Africa. South African Med J [Internet]. 2009 Jan 1;99(8):10. Available from: http://search.ebscohost.com/login.aspx?direct=true&db=awn&AN=803234-1&site=ehost-live&scope=site

46. Hilton J. Factors that influence the quality of life of a caregiver caring for a patient with stroke. 2011

47. Cunningham NL. The profile and outcomes of stroke patients discharged from a hospital in the Eastern Cape [Internet]. 2012. Available from: http://search.ebscohost.com/login.aspx?direct=true&db=awn&AN=NX0215155&site=ehost-live&scope=site

48. de Villiers L, Kalula SZ, Burch VC. Does multidisciplinary stroke care improve

For peer review only - http://bmjopen.bmj.com/site/about/guidelines.xhtml

outcome in a secondary-level hospital in South Africa? [Internet]. Vol. 4, International journal of stroke: official journal of the International Stroke Society. 2009. p. 89–93. Available from: http://search.ebscohost.com/login.aspx?direct=true&db=awn&AN=19383048&site=ehost-live&scope=site

49. Rhoda A, Smith M, Putman K, Mpofu R, DeWeerdt W, DeWit L. Motor and functional recovery after stroke: a comparison between rehabilitation settings in a developed versus a developing country [Internet]. Vol. 14, BMC Health Services Research. 2014. p. 82. Available from: http://search.ebscohost.com/login.aspx?direct=true&db=awn&AN=24559193&site=ehost-live&scope=site

50. Mudzi W. Impact of caregiver education on stroke survivors and their caregivers. Dissertation. 2010

51. Parekh I, Rhoda A. Functional outcomes of stroke patients admitted to a tertiary hospital in the Western Cape, South Africa [Internet]. Vol. 69, South African Journal of Physiotherapy. 2013. p. 14. Available from: http://search.ebscohost.com/login.aspx?direct=true&db=awn&AN=722250&site=ehost-live&scope=site

52. Arowoiya A. Participation restrictions of stroke patients living in the community at selected community health centres in the metropole districts in the Western Cape, South Africa. Diss Univ West Cape. 2014

53. Mudzi W, Stewart A, Musenge E. Community participation of patients 12 months post-stroke in Johannesburg, South Africa. African J Prim Heal Care Fam Med. 2013;5(1):1–9.

54. Parekh IS. Factors influencing functional outcome of stroke patients admitted to a tertiary hospital [Internet]. 2012. Available from: http://search.ebscohost.com/login.aspx?direct=true&db=awn&AN=NX0214919&site=ehost-live&scope=site

55. Rhoda A, Mpofu R, De Weerdt W. Activity limitations of patients with stroke attending out-patient facilities in the Western Cape, South Africa [Internet]. Vol. 67, South African Journal of Physiotherapy. 2011. p. 16–22. Available from: http://search.ebscohost.com/login.aspx?direct=true&db=awn&AN=702551&site=ehost-live&scope=site

56. Blackwell Z, Littlejohns P. A review of the management of dysphagia: a South African perspective [Internet]. Vol. 42, Journal of Neuroscience Nursing. 2010. p. 61–70. Available from: http://search.ebscohost.com/login.aspx?direct=true&db=awn&AN=20422791&site=ehost-live&scope=site

For peer review only

For peer review only - http://bmjopen.bmj.com/site/about/guidelines.xhtml

57. Felemengas M. Caregiver experiences and perceptions of the effects of stroke in the family [Internet]. 2004. Available from: http://search.ebscohost.com/login.aspx?direct=true&db=awn&AN=NX0150149&site=ehost-live&scope=site

58. Mabunda SA. An Evaluation of the Role of an Intermediate Care Facility in the Continuum of Care in Western Cape, South Africa. Dissertation [Internet]. 2015;53(July):227. Available from: http://library.wur.nl/WebQuery/wurpubs/fulltext/353506

59. Groenewald RC. The Adaptation and Contextualization of the Bridges Stroke Self-Management Intervention for Patients Living With Stroke in the Western Cape, South Africa. 2018;(August). Available from: http://etd.uwc.ac.za/ii

60. Makganye TM. The experience of patients and caregivers following a stroke. 2015;(December).

61. Kotsokoane FM, Tshabalala MD, Nukeri AL, Mkhacwa WB. The level of integration of stroke survivors receiving rehabilitation services in Soshanguve clinics, South Africa [Internet]. Vol. 24, African Journal for Physical, Health Education, Recreation and Dance. 2018. p. 564–78. Available from: http://search.ebscohost.com/login.aspx?direct=true&db=awn&AN=ajpherd-181896&site=ehost-live&scope=site

62. Kusambiza-kiingi A. Community reintegration and satisfaction of survivors of stroke receiving physiotherapy services in the community health centres within the Johannesburg area. 2016;1–2.

63. Ntamo PN. Poor attendance of physiotherapy treatment by stroke out-patients in Mthatha General Hospital [Internet]. 2011. Available from: http://search.ebscohost.com/login.aspx?direct=true&db=awn&AN=NX0207219&site=ehost-live&scope=site

64. Taylor A, Ntusi NAB. Guest Editorial: Evolving concepts of stroke and stroke management in South Africa: Quo vadis? [Internet]. Vol. 109, South African Medical Journal. 2019. p. 69–71. Available from: http://search.ebscohost.com/login.aspx?direct=true&db=awn&AN=samj-184307&site=ehost-live&scope=site

65. Biggs D. Health Promotion Needs of Stroke Patients Accessing Community Health Centres in the Metropole Region of the Western Cape Health Promotion Needs of Stroke Patients Accessing Community Health Centres in the. 2005;(November).

66. Thomas M, Greenop K. Caregiver Experiences and Perceptions of Stroke. Heal SA Gesondheid [Internet]. 2008 Jan 1;13(1):29–40. Available from: http://search.ebscohost.com/login.aspx?direct=true&db=awn&AN=IM-009014&site=ehost-live&scope=site

For peer review only - http://bmjopen.bmj.com/site/about/guidelines.xhtml

67. Posner G. the Experiences of Employed Caregivers, Working in Private South African Homes, With Patients Who Have Suffered From a Stroke. 2015; Available from: http://wiredspace.wits.ac.za/jspui/bitstream/10539/19598/2/MASTERS DISSERTATION G POSNER.pdf

68. Smith JL. Self-management strategies employed by stroke survivors in the Western Cape, South Africa. Diss Univ Cape T. 2019

69. Mashau NS, Netshandama VO, Mudau MJ. Self-reported impact of caregiving on voluntary home-based caregivers in Mutale Municipality, South Africa. African J Prim Heal Care Fam Med. 2016;8(2):1–5.

70. Maleka M, Stewart AS, Hale L. The experience of living with stroke in low urban and rural socioeconomic areas of South Africa [Internet]. South African Journal of Physiotherapy. 2012. p. 5–29. Available from: http://search.ebscohost.com/login.aspx?direct=true&db=awn&AN=705374&site=ehost-live&scope=site

71. Rhoda AJ. Health-related quality of life of patients six months poststroke living in the Western Cape, South Africa. African J Disabil. 2014;3(1):1–6.

72. Kusambiza-Kiingi A, Maleka D, Ntsiea V. Stroke survivors' levels of community reintegration, quality of life, satisfaction with the physiotherapy services and the level of caregiver strain at community health centres within the Johannesburg area. African J Disabil. 2017;6:1–8.

73. Hassan S, Visagie S, Mji G. Strain experienced by caregivers of stroke survivors in the Western Cape [Internet]. Vol. 67, South African Journal of Physiotherapy. 2011. p. 4–8. Available from: http://search.ebscohost.com/login.aspx?direct=true&db=awn&AN=702553&site=ehost-live&scope=site

74. Masuku KP, Mophosho M, Tshabalala M. "I felt pain. Deep pain...": Experiences of primary caregivers of stroke survivors with aphasia in a South African township. African J Disabil. 2018;7:1–7.

75. Biggs D, Rhoda A. Health risk behaviours of stroke patients in the Western Cape, South Africa. South African J Physiother [Internet]. 2008 Jan 1;64(1):38–42. Available from: http://search.ebscohost.com/login.aspx?direct=true&db=awn&AN=577099&site=ehost-live&scope=site

76. Burton A. South Africa: stroke units out of the blue [Internet]. Vol. 15, The Lancet. Neurology. 2016. p. 359–60. Available from: http://search.ebscohost.com/login.aspx?direct=true&db=awn&AN=26971657&site=ehost-live&scope=site

77. Elloker T. Social support and participation restrictions in patients living with stroke in

For peer review only

For peer review only - http://bmjopen.bmj.com/site/about/guidelines.xhtml

the Western Cape, SOuth Africa. Dissertation. 2015

78. Cunningham N, Rhoda A. Outcomes of stroke patients discharged from an in-patient facility in the Eastern Cape, South Africa: a mixed methods design [Internet]. Vol. 70, South African Journal of Physiotherapy. 2014. p. 26–31. Available from: http://search.ebscohost.com/login.aspx?direct=true&db=awn&AN=722129&site=ehost-live&scope=site

79. Mamabolo V. The Influence of Demographic, Environmental and Physical Factors on Functional Independence Post Stroke Correspondence to : 2008;(011):19–22.

80. Botha JH. The refinement of a booklet on stroke care at home. 2008

81. Faux S. Managing stroke survivors int he community. Mod Med South Africa [Internet]. 2006 Jan 1;31(1 January):8–14. Available from: http://search.ebscohost.com/login.aspx?direct=true&db=awn&AN=D-18283&site=ehost-live&scope=site

82. Bryer A, Connor M, Haug P, Cheyip B, Staub H, Tipping B, et al. South African guideline for management of ischaemic stroke and transient ischaemic attack 2010: a guideline from the South African Stroke Society (SASS) and the SASS Writing Committee [Internet]. Vol. 100, S A M J South African Medical Journal. 2010. p. 747–78. Available from: http://search.ebscohost.com/login.aspx?direct=true&db=awn&AN=21081029&site=ehost-live&scope=site

83. Hussey M, Maclachlan M, Mji G. Barriers to the Implementation of the Health and Rehabilitation Articles of the United Nations Convention on the Rights of Persons with Disabilities in South Africa. Kerman Univ Med Sci [Internet]. 2017;6(4):207–218. Available from: http://dx.doi.org/10.15171/ijhpm.2016.117

84. National Department of Health. Strengthening the South African health system: Towards an integrated and unified health system. Pres Heal Summit 2018.

85. Vindigni SM, Riley PL, Kimani F, Willy R, Warutere P, Sabatier JF, et al. Kenya's emergency-hire nursing programme: A pilot evaluation of health service delivery in two districts. Hum Resour Health. 2014

86. Lehmann U, Dieleman M, Martineau T. Staffing remote rural areas in middle- and low-income countries: A literature review of attraction and retention. BMC Health Serv Res. 2008

87. Hatcher AM, Onah M, Kornik S, Peacocke J, Reid S. Placement, support, and retention of health professionals: National, cross-sectional findings from medical and dental community service officers in South Africa. Hum Resour Health. 2014

88. Chimatiro GL, Rhoda AJ. Scoping review of acute stroke care management and rehabilitation in low and middle-income countries. BMC Health Serv Res. 2019

For peer review only - http://bmjopen.bmj.com/site/about/guidelines.xhtml

Nov;19(1):789.

89. Connor M. Stroke management in South Africa - who is responsible?: guest editorial [Internet]. Vol. 8, South African Psychiatry Review. 2005. p. 125–6. Available from: http://search.ebscohost.com/login.aspx?direct=true&db=awn&AN=381538&site=ehost-live&scope=site

90. Bates B, Choi JY, Duncan PW, Glasberg JJ, Graham GD, Katz RC, et al. Veterans Affairs/Department of Defense Clinical Practice Guideline for the Management of Adult Stroke Rehabilitation Care: Executive summary. Stroke. 2005.

91. Maclean N, Pound P, Wolfe C, Rudd A. Qualitative analysis of stroke patients' motivation for rehabilitation. Br Med J. 2000

LEGEND

Figure 1: Components of the analytical framework that incorporates components from the Health Systems Dynamics Framework (19) and WHO Framework on integrated people-centred health services (20).

Figure 2: PRISMA flow chart

Figure 3: Limiting and supporting factors toward achieving UHC

Supplementary File

Supplementary File S1: Search Strategy

Supplementary File S2: Framework component definitions

Supplementary file S3: Basic demographic information of included records (N=59)

Supplementary file S4: Components assessed in included studies

For peer review only

For peer review only - http://bmjopen.bmj.com/site/about/guidelines.xhtml

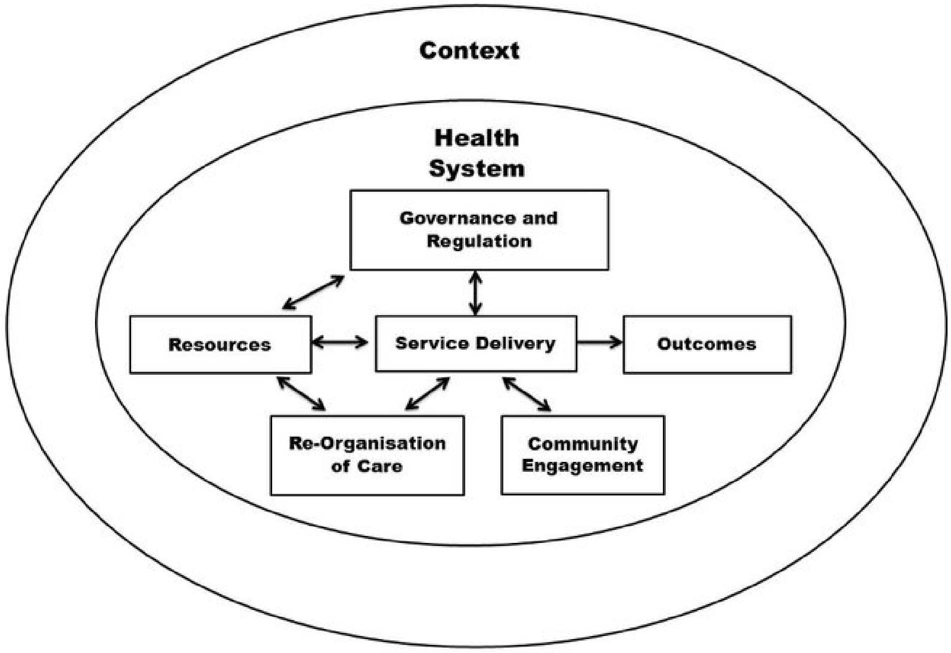

Figure 1: Components of the analytical framework that incorporates components from the Health Systems Dynamics Framework (19) and WHO Framework on integrated people-centred health services (20). Identifying the research question

182x136mm (96 x 96 DPI)

Figure-2: PRISMA flowchart of the scoping review.

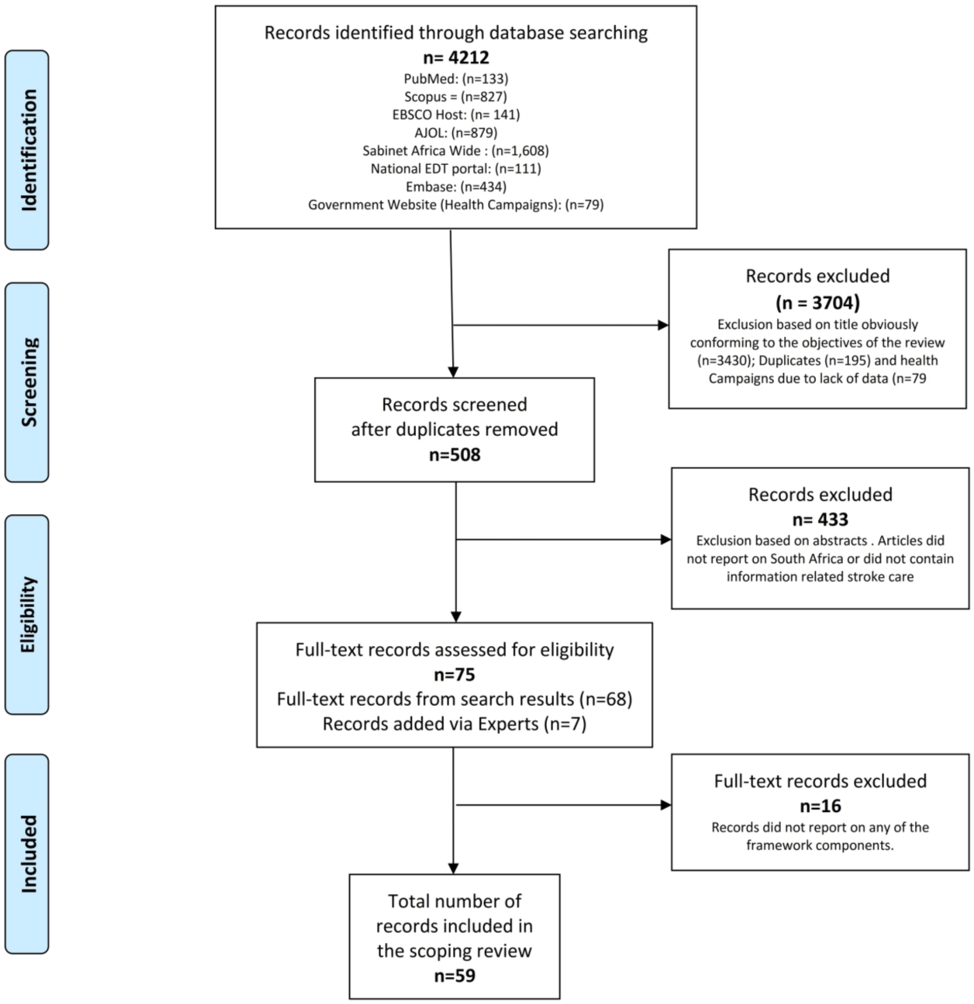

Figure 2: PRISMA flow chart

191x201mm (300 x 300 DPI)

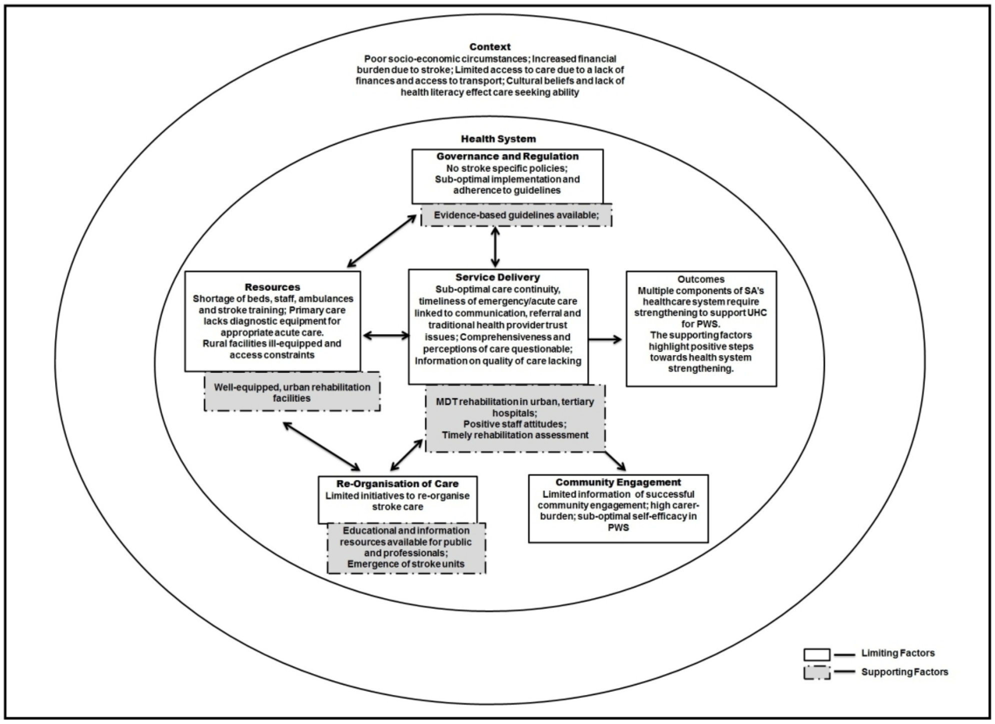

Figure 3: Limiting and supporting factors toward achieving UHC

531x388mm (96 x 96 DPI)

Supplemantary File - 1

Achieving Universal Health Coverage for people with stroke in South Africa:

OVID Search

Database: Embase <1980 to 2020 Week 24>, Global Health <1910 to 2020 Week 23>, Journals@Ovid Full Text <June 17, 2020>, APA PsycExtra <1908 to June 08, 2020>, APA PsycInfo <1806 to June Week 2 2020>, LSHTM Journals@Ovid, Econlit <1886 to June 11, 2020>, Ovid MEDLINE(R) and Epub Ahead of Print, In-Process & Other Non-Indexed Citations, Daily and Versions(R) <1946 to June 16, 2020>, Social Policy and Practice <202004>
Search Strategy:
----

1 Stroke.mp. [mp=ti, ab, hw, tn, ot, dm, mf, dv, kw, fx, dq, bt, id, cc, tx, sh, ct, tc, tm, mh, nm, kf, ox, px, rx, an, ui, sy, pt] (1263546)
2 cerebro vascular accident.mp. [mp=ti, ab, hw, tn, ot, dm, mf, dv, kw, fx, dq, bt, id, cc, tx, sh, ct, tc, tm, mh, nm, kf, ox, px, rx, an, ui, sy, pt] (560)
3 ischaemia.mp. [mp=ti, ab, hw, tn, ot, dm, mf, dv, kw, fx, dq, bt, id, cc, tx, sh, ct, tc, tm, mh, nm, kf, ox, px, rx, an, ui, sy, pt] (140097)
4 hemorrhage.mp. [mp=ti, ab, hw, tn, ot, dm, mf, dv, kw, fx, dq, bt, id, cc, tx, sh, ct, tc, tm, mh, nm, kf, ox, px, rx, an, ui, sy, pt] (923222)
5 Universal health coverage.mp. [mp=ti, ab, hw, tn, ot, dm, mf, dv, kw, fx, dq, bt, id, cc, tx, sh, ct, tc, tm, mh, nm, kf, ox, px, rx, an, ui, sy, pt] (9467)
6 universal access.mp. [mp=ti, ab, hw, tn, ot, dm, mf, dv, kw, fx, dq, bt, id, cc, tx, sh, ct, tc, tm, mh, nm, kf, ox, px, rx, an, ui, sy, pt] (12850)
7 Universal health care.mp. [mp=ti, ab, hw, tn, ot, dm, mf, dv, kw, fx, dq, bt, id, cc, tx, sh, ct, tc, tm, mh, nm, kf, ox, px, rx, an, ui, sy, pt] (7370)
8 universal health access.mp. [mp=ti, ab, hw, tn, ot, dm, mf, dv, kw, fx, dq, bt, id, cc, tx, sh, ct, tc, tm, mh, nm, kf, ox, px, rx, an, ui, sy, pt] (64)
9 stroke disability.mp. [mp=ti, ab, hw, tn, ot, dm, mf, dv, kw, fx, dq, bt, id, cc, tx, sh, ct, tc, tm, mh, nm, kf, ox, px, rx, an, ui, sy, pt] (1581)
10 stroke rehabilitation.mp. [mp=ti, ab, hw, tn, ot, dm, mf, dv, kw, fx, dq, bt, id, cc, tx, sh, ct, tc, tm, mh, nm, kf, ox, px, rx, an, ui, sy, pt] (30385)
11 stroke care.mp. [mp=ti, ab, hw, tn, ot, dm, mf, dv, kw, fx, dq, bt, id, cc, tx, sh, ct, tc, tm, mh, nm, kf, ox, px, rx, an, ui, sy, pt] (18395)
12 treatment access.mp. [mp=ti, ab, hw, tn, ot, dm, mf, dv, kw, fx, dq, bt, id, cc, tx, sh, ct, tc, tm, mh, nm, kf, ox, px, rx, an, ui, sy, pt] (6626)
13 health systems.mp. [mp=ti, ab, hw, tn, ot, dm, mf, dv, kw, fx, dq, bt, id, cc, tx, sh, ct, tc, tm, mh, nm, kf, ox, px, rx, an, ui, sy, pt] (111350)
14 South Africa.mp. [mp=ti, ab, hw, tn, ot, dm, mf, dv, kw, fx, dq, bt, id, cc, tx, sh, ct, tc, tm, mh, nm, kf, ox, px, rx, an, ui, sy, pt] (235927)
15 Eastern Cape.mp. [mp=ti, ab, hw, tn, ot, dm, mf, dv, kw, fx, dq, bt, id, cc, tx, sh, ct, tc, tm, mh, nm, kf, ox, px, rx, an, ui, sy, pt] (4355)
16 Free State.mp. [mp=ti, ab, hw, tn, ot, dm, mf, dv, kw, fx, dq, bt, id, cc, tx, sh, ct, tc, tm, mh, nm, kf, ox, px, rx, an, ui, sy, pt] (14110)
17 Gauteng.mp. [mp=ti, ab, hw, tn, ot, dm, mf, dv, kw, fx, dq, bt, id, cc, tx, sh, ct, tc, tm, mh, nm, kf, ox, px, rx, an, ui, sy, pt] (4356)
18 KwaZulu-Natal.mp. [mp=ti, ab, hw, tn, ot, dm, mf, dv, kw, fx, dq, bt, id, cc, tx, sh, ct, tc, tm, mh, nm, kf, ox, px, rx, an, ui, sy, pt] (11901)

For peer review only

19 Limpopo.mp. [mp=ti, ab, hw, tn, ot, dm, mf, dv, kw, fx, dq, bt, id, cc, tx, sh, ct, tc, tm, mh, nm, kf, ox, px, rx, an, ui, sy, pt] (3368)
20 Mpumalanga.mp. [mp=ti, ab, hw, tn, ot, dm, mf, dv, kw, fx, dq, bt, id, cc, tx, sh, ct, tc, tm, mh, nm, kf, ox, px, rx, an, ui, sy, pt] (1992)
21 Northern Cape.mp. [mp=ti, ab, hw, tn, ot, dm, mf, dv, kw, fx, dq, bt, id, cc, tx, sh, ct, tc, tm, mh, nm, kf, ox, px, rx, an, ui, sy, pt] (997)
22 North West.mp. [mp=ti, ab, hw, tn, ot, dm, mf, dv, kw, fx, dq, bt, id, cc, tx, sh, ct, tc, tm, mh, nm, kf, ox, px, rx, an, ui, sy, pt] (39969)
23 Western Cape.mp. [mp=ti, ab, hw, tn, ot, dm, mf, dv, kw, fx, dq, bt, id, cc, tx, sh, ct, tc, tm, mh, nm, kf, ox, px, rx, an, ui, sy, pt] (8316)
24 1 or 2 or 3 or 4 (2162628)
25 5 or 6 or 7 or 8 (28371)
26 9 or 10 or 11 or 12 (54898)
27 15 or 16 or 17 or 18 or 19 or 20 or 21 or 22 or 23 (83478)
28 14 or 27 (286527)
29 13 or 25 (135636)
30 24 and 29 (7926)
31 24 and 26 and 29 (655)
32 24 and 28 and 29 (433)

****

For peer review only

UHC for Stroke care in SA - Scopus Search results

(Stroke OR Universal health coverage) AND (Health system) AND (LIMIT-TO (AFFILCOUNTRY,"South Africa")) AND (LIMIT-TO (PUBYEAR,2020) OR LIMIT-TO (PUBYEAR,2019) OR LIMIT-TO (PUBYEAR,2018) OR LIMIT-TO (PUBYEAR,2017) OR LIMIT-TO (PUBYEAR,2016) OR LIMIT-TO (PUBYEAR,2015) OR LIMIT-TO (PUBYEAR,2014) OR LIMIT-TO (PUBYEAR,2013) OR LIMIT-TO (PUBYEAR,2012) OR LIMIT-TO (PUBYEAR,2011) OR LIMIT-TO (PUBYEAR,2010) OR LIMIT-TO (PUBYEAR,2009) OR LIMIT-TO (PUBYEAR,2008) OR LIMIT-TO (PUBYEAR,2007) OR LIMIT-TO (PUBYEAR,2006) OR LIMIT-TO (PUBYEAR,2004) OR LIMIT-TO (PUBYEAR,2003) OR LIMIT-TO (PUBYEAR,2002) OR LIMIT-TO (PUBYEAR,2000)) AND (LIMIT-TO (openaccess,1)) AND (LIMIT-TO (SUBJAREA,"MEDI") OR LIMIT-TO (SUBJAREA,"SOCI") OR LIMIT-TO (SUBJAREA,"MULT") OR LIMIT-TO (SUBJAREA,"NURS") OR LIMIT-TO (SUBJAREA,"HEAL") OR LIMIT-TO (SUBJAREA,"ENVI") OR LIMIT-TO (SUBJAREA,"PSYC") OR LIMIT-TO (SUBJAREA,"ECON") OR LIMIT-TO (SUBJAREA,"ARTS") OR LIMIT-TO (SUBJAREA,"NEUR")) AND (LIMIT-TO (PUBSTAGE,"final") OR LIMIT-TO (PUBSTAGE,"aip"))

For peer review only

Print Search History: EBSCOhost

		Narrow by SubjectThesaurus: - qualitative research<br>Narrow by SubjectThesaurus: - primary care<br>Narrow by SubjectThesaurus: - evaluation research<br>Narrow by SubjectThesaurus: - health behavior<br>Narrow by SubjectThesaurus: - community health services<br>Narrow by SubjectThesaurus: - health outcome assessment<br>Narrow by SubjectThesaurus: - government policy<br>Narrow by SubjectThesaurus: - world health<br>Narrow by SubjectThesaurus: - quality of life<br>Narrow by SubjectThesaurus: - health services accessibility<br>Narrow by SubjectThesaurus: - health promotion<br>Narrow by SubjectThesaurus: - public health<br>Search modes - Boolean/Phrase		
S5	(stroke or cerebrovascular accident or cva or cerebral vascular event or cve or transient ischaemic attack or tia) OR (stroke rehabilitation or stroke recovery) OR stroke	Limiters - Published Date: 20050101-20200631<br>Expanders - Apply related words; Apply equivalent subjects<br>Narrow by Language: - english	Interface - EBSCOhost Research Databases<br>Search Screen - Advanced Search<br>Database - Academic Search Complete	16,739

prevention OR stroke patients AND universal health care OR universal health coverage OR universal health coverage in south africa AND health system OR health systems strengthening OR (health system or health services) OR health systems management AND south africa	Narrow by SubjectThesaurus: - public health surveillance<br>Narrow by SubjectThesaurus: - primary health care<br>Narrow by SubjectThesaurus: - national health services<br>Narrow by SubjectThesaurus: - health insurance<br>Narrow by SubjectThesaurus: - health disparities<br>Narrow by SubjectThesaurus: - health care reform<br>Narrow by SubjectThesaurus: - health programs<br>Narrow by SubjectThesaurus: - chronic diseases<br>Narrow by SubjectThesaurus: - economics<br>Narrow by SubjectThesaurus: - systematic reviews (medical research)<br>Narrow by SubjectThesaurus: - qualitative research<br>Narrow by SubjectThesaurus: - primary care<br>Narrow by SubjectThesaurus: - evaluation research<br>Narrow by SubjectThesaurus: - health behavior<br>Narrow by SubjectThesaurus: - community health services<br>Narrow by SubjectThesaurus: -

		health outcome assessment<br>Narrow by SubjectThesaurus: - government policy<br>Narrow by SubjectThesaurus: - world health<br>Narrow by SubjectThesaurus: - quality of life<br>Narrow by SubjectThesaurus: - health services accessibility<br>Narrow by SubjectThesaurus: - health promotion<br>Narrow by SubjectThesaurus: - public health<br>Search modes - Boolean/Phrase		
S4	(stroke or cerebrovascular accident or cva or cerebral vascular event or cve or transient ischaemic attack or tia) OR (stroke rehabilitation or stroke recovery) OR stroke prevention OR stroke patients AND universal health care OR universal health coverage OR universal health coverage in south africa AND health system OR health systems strengthening OR (health system or health services) OR health systems management AND south africa	Limiters - Published Date: 20050101-20200631<br>Expanders - Apply related words; Apply equivalent subjects<br>Narrow by SubjectThesaurus: - public health surveillance<br>Narrow by SubjectThesaurus: - primary health care<br>Narrow by SubjectThesaurus: - national health services<br>Narrow by SubjectThesaurus: - health insurance<br>Narrow by SubjectThesaurus: - health disparities<br>Narrow by SubjectThesaurus: - health care reform<br>Narrow by SubjectThesaurus: -	Interface - EBSCOhost Research Databases<br>Search Screen - Advanced Search<br>Database - Academic Search Complete	17,251

Print Search History: EBSCOhost

health programs
Narrow by
SubjectThesaurus: -
chronic diseases
Narrow by
SubjectThesaurus: -
economics
Narrow by
SubjectThesaurus: -
systematic reviews
(medical research)
Narrow by
SubjectThesaurus: -
qualitative research
Narrow by
SubjectThesaurus: -
primary care
Narrow by
SubjectThesaurus: -
evaluation research
Narrow by
SubjectThesaurus: -
health behavior
Narrow by
SubjectThesaurus: -
community health
services
Narrow by
SubjectThesaurus: -
health outcome
assessment
Narrow by
SubjectThesaurus: -
government policy
Narrow by
SubjectThesaurus: -
world health
Narrow by
SubjectThesaurus: -
quality of life
Narrow by
SubjectThesaurus: -
health services
accessibility
Narrow by
SubjectThesaurus: -
health promotion
Narrow by
SubjectThesaurus: -

Print Search History: EBSCOhost

		public health<br>Search modes - Boolean/Phrase		
S3	(stroke or cerebrovascular accident or cva or cerebral vascular event or cve or transient ischaemic attack or tia) OR (stroke rehabilitation or stroke recovery) OR stroke prevention OR stroke patients AND universal health care OR universal health coverage OR universal health coverage in south africa AND health system OR health systems strengthening OR (health system or health services) OR health systems management AND south africa	Limiters - Published Date: 20050101-20200631<br>Expanders - Apply related words; Apply equivalent subjects<br>Narrow by SubjectThesaurus: - public health<br>Search modes - Boolean/Phrase	Interface - EBSCOhost Research Databases<br>Search Screen - Advanced Search<br>Database - Academic Search Complete	84,912
S2	(stroke or cerebrovascular accident or cva or cerebral vascular event or cve or transient ischaemic attack or tia) OR (stroke rehabilitation or stroke recovery) OR stroke prevention OR stroke patients AND universal health care OR universal health coverage OR universal health coverage in south africa AND health system OR health systems strengthening OR (health system or health services) OR health systems management AND south africa	Limiters - Published Date: 20050101-20200631<br>Expanders - Apply related words; Apply equivalent subjects<br>Search modes - Boolean/Phrase	Interface - EBSCOhost Research Databases<br>Search Screen - Advanced Search<br>Database - Academic Search Complete	878,271
S1	(stroke or cerebrovascular accident or cva or cerebral	Limiters - Published Date: 20050101-20200631	Interface - EBSCOhost Research Databases<br>Search Screen - Advanced	878,271

vascular event or cve or transient ischaemic attack or tia) OR (stroke rehabilitation or stroke recovery) OR stroke prevention OR stroke patients AND universal health care OR universal health coverage OR universal health coverage in south africa AND health system OR health systems strengthening OR (health system or health services) OR health systems management OR health system financing	Expanders - Apply related words; Apply equivalent subjects Search modes - Boolean/Phrase	Search Database - Academic Search Complete

Supplementary file S2

FRAMEWORK COMPONENT	DESCRIPTION (data items)
**Governance/Regulation**	**Description:** a wide range of steering and rulemaking-related functions carried out by governments/decisions makers as they seek to achieve national and/or provincial health policy objectives that are conducive to UHC<br>**Data Items:** Healthcare policies at national or provincial levels; resource allocation policies; accountability monitoring; coordination and regulations; clinical treatment guidelines.
**Resources**	**Description:** All resources specific to the health care facility – including the physical structure and resources enabling or hindering delivery of health services.<br>**Data Items:**<br>**Infrastructure:** Accessibility of the health care facilities; maintenance of infrastructure; availability of equipment/testing facilities (e.g. CT Scans)<br>**Human resources:** Availability; health workforce distribution – health professions/experience or specialisation/gender; role definitions; undergraduate & continuous training; workload; patient vs therapist ratio.<br>**Financial resources:** Finance allocation and affordability; funding sources; healthcare packages; salaries/fair wages.; sustainability.
**Service Delivery**	**Description:** Delivery of different health services as well as user experience.<br>**Data Items:** Level of care; comprehensiveness; quality and/or perceptions of care; multi-professional health teams; continuity of care, timeliness of care; health services and service providers (private/public; for-profit or not–for-profit, formal or informal, professional or non-professional, allopathic or traditional, remunerated or voluntary).
**Context**	**Description:** All contextual factors influencing the patient/community access of the health care system.<br>**Data Items:** Social determinants of health: socio-economic, education, health literacy, technological, cultural, political and environmental environments.
**Re-orientation of care**	**Description:** New and innovate health care solutions to improve coordination of health services and continuous health care; and intersectoral coordination.<br>**Data Items:** New technologies and strategies (eHealth; shared electronic medical records; telemedicine; m-health)
**Community engagement**	**Description:** Engaging and empowering individuals, families, communities and informal caregivers to facilitate common decision-making and self-efficacy. Reaching underserved and marginalised communities.

Application of the key characteristics of the analytical framework

For peer review only

FRAMEWORK COMPONENT	DESCRIPTION (data items)
**Governance/Regulation**	**Description:** a wide range of steering and rulemaking-related functions carried out by governments/decisions makers as they seek to achieve national and/or provincial health policy objectives that are conducive to UHC<br>**Data Items:** Healthcare policies at national or provincial levels; resource allocation policies; accountability monitoring; coordination and regulations; clinical treatment guidelines.
**Resources**	**Description:** All resources specific to the health care facility – including the physical structure and resources enabling or hindering delivery of health services.<br>**Data Items:**<br>**Infrastructure:** Accessibility of the health care facilities; maintenance of infrastructure; availability of equipment/testing facilities (e.g. CT Scans)<br>**Human resources:** Availability; health workforce distribution – health professions/experience or specialisation/gender; role definitions; undergraduate & continuous training; workload; patient vs therapist ratio.<br>**Financial resources:** Finance allocation and affordability; funding sources; healthcare packages; salaries/fair wages.; sustainability.
**Service Delivery**	**Description:** Delivery of different health services as well as user experience.<br>**Data Items:** Level of care; comprehensiveness; quality and/or perceptions of care; multiprofessional health teams; referral systems; service delivery models; health services and service providers (private/public; for-profit or not–for-profit, formal or informal, professional or non-professional, allopathic or traditional, remunerated or voluntary).
**Context**	**Description:** All contextual factors influencing the patient/community access of the health care system.<br>**Data Items:** Social determinants of health: socio-economic, education, health literacy, technological, cultural, political and environmental environments.
**Re-orientation of care**	**Description:** New and innovate health care solutions to improve coordination of health services and continuous health care; and intersectoral coordination.<br>**Data Items:** New technologies and strategies (eHealth; shared electronic medical records; telemedicine; m-health)
**Community engagement**	**Description:** Engaging and empowering individuals, families, communities and informal caregivers to facilitate common decision-making and self-efficacy. Reaching underserved and marginalised communities.

For peer review only

Supplementary file S3. Detailed demographic information of all included records (N=59)

S.no	Author (year)	Province	Area	Level of care	Literature	Aim	Study design	Sample characteristics
1	Arowoiya (2014)	Western Cape	Urban	Primary Healthcare (CHC)	Dissertations	To determine and explore the participation restrictions experienced by stroke patients	Mixed Methods (Survey + FGDs)	120 stroke patients receiving Physiotherapy for survey & 2 FGDs with 17 stroke patients
2	Bham & Ross 2005	Not reported	Undefined	Community	Primary Literature (peer reviewed publications)	To investigate the beliefs of caregivers and traditional healers within the South African Indian Muslim community regarding the etiology and treatment of stroke and the persons likely to be consulted in this regard	Descriptive case study design (Qualitative)	10 SAIM caregivers of people who had sustained strokes, as well as 10 SAIM traditional healers, who had treated stroke patients.
3	Biggs (2005)	Western Cape	Urban	Primary Healthcare (CHC)	Dissertations	To determine the health promotion needs of stroke patients accessing selected Community Health Centres in the Metropole region of the Western Cape.	Mixed Methods (Survey + In-depth interview)	418 stroke patients, representing each of the health districts of the Metropole region of the Western Cape for the survey and 12 stroke patients for Interviews
4	Biggs & Rhoda 2008	Western Cape	Urban	Primary Healthcare (CHC)	Primary Literature (peer reviewed publications)	To determine the health risk behaviours and factors that influence these behaviours of stroke patients in the Metropole Region of the Western Cape, South Africa	Mixed Methods (Survey + In-depth interview)	417 stroke patients – survey 12 stroke patients for Interviews
5	Blackwell & Littlejohns 2010	Gauteng,	Undefined	Undefined	Primary Literature (peer reviewed publications)	To measure the prevalence and review the assessment and management strategies related to dysphagia in three private rehabilitation clinics in South Africa	Review of records and thematic analysis	30 stroke patient records from three private rehabilitation clinics – total 90 records
6	Botha (2008)	Western Cape	Undefined	Community	Dissertations	To refine and pilot a training booklet for caregivers of stroke survivors for further	Mixed methods (Literature review +	Sample – 1: 11 Stroke patients in WCRC Sample – 2: 1Family members /other

For peer review only

						implementation	Checklist development + survey + FGDs)	carers of Stroke patients in WCRC<br>Sample – 3: 4 Home based carers and 22 nursing assistants attending carer training at WCRC<br>Sample – 4: 4 Stroke patients and their 4 caregivers participating in home-based care programme at university of Western Cape in Nyanga<br>Sample -5: Stroke patients in WCRC<br>Total 15 stroke patients and 31 caregivers
7	Bryer (2009)	South Africa	Undefined	Undefined	Primary Literature (peer reviewed publications)	There is an urgent need to develop a model of community-based stroke care with appropriate<br>Rehabilitation facilities and trained professionals<br>In South Africa, particularly in under-resourced<br>areas	Editorial	NA
8	Bryer et al (2010)	South Africa	NA	NA	Primary Literature (peer reviewed publications, Clinical Guideline)	The objective was to update the guideline published in 2000, to place the recommendations within the current South African context, and to grade evidence according to the level of scientific rigour for management of ischaemic stroke and transient ischaemic attack 2010	SASS writing committee Guidelines	NA
9	Burton 2016	South Africa	Undefined	Undefined	Primary Literature (peer reviewed publications)	To investigate the efforts of a woman with a talent for getting things done for bringing stroke units out of the blue for South Africa	Editorial	NA
10	Cawood 2012	Western Cape	Urban	Community	Dissertations	To determine if uninsured stroke survivors living in the Helderberg Basin (Western Cape) reached their optimal	A descriptive, mixed methods study (Surev + Interview)	53 stroke survivors (quantitative)<br>5 Stroke survivors (qualitative)

For peer review only

						rehabilitation outcome levels and if not, what environmental barriers contributed to this.		
11	Cawood& Visagie (2015)	Western Cape	Urban	Community	Primary Literature (peer reviewed publications)	To determine environmental barriers and facilitators to participation experienced by a group of stroke survivors in the Western Cape province of South Africa.	A descriptive, mixed methods study (Surev + Interview)	53 stroke survivors (quantitative)<br>5 Stroke survivors (qualitative)
12	Cawood & Visagie (2016)	Western Cape	Urban	Community	Primary Literature (peer reviewed publications)	To describe the functional outcomes achieved by stroke survivors in an urban Western Cape Province setting to add to the information on stroke management	A descriptive, mixed methods study (Surev + Interview)	53 stroke survivors (quantitative)<br>5 Stroke survivors (qualitative)
13	Cawood et al (2016)	Western Cape	Urban	Community	Primary Literature (peer reviewed publications)	To explore causal connections between impairments, activity limitations and participation restrictions after stroke.	Cross-sectional Study	53 stroke survivors
14	Connor (2005)	Sub Saharan Africa	Undefined	Undefined	Primary Literature (peer reviewed publications)	To understand the burden of stroke in black populations in sub-Saharan Africa	Systematic Review	All articles relevant to stroke in Sub Saharan Africa
15	Cunningham (2012)	Eastern cape	Urban	Uitenhage Provincial Hospital	Dissertations	To determine and explore the outcomes of stroke patients admitted to Uitenhage provincial hospital	Mixed Methods (quantitative survey + secondary data analysis + Semi-structured interviews)	168 stroke patient records for secondary data analysis, 24 stroke patients for prospective survey and 9 stroke patients for the qualitative study
16	Cunningham & Rhoda 2014	Eastern Cape	Urban	Community	Primary Literature (peer reviewed publications)	To determine the outcome of stroke patients in Eastern cape	Concurrent Mixed Methods design	24 Stroke Patients (Quantitative Survey)<br>9 Stroke patients (Qualitative interviews)
17	De la Cornillère (2007)	Western cape	Urban	BLRC Rehab centre	Dissertations	To describe the range of experiences of stroke patients relating to attendance or non-attendance of those referred to the Bishop Lavis Rehabilitation centre stroke group	Mixed Methods Descriptive study	20 participants with stroke for questionnaire survey and 6 stroke participants for FGD.

For peer review only

18	De Villiers et al 2009	Cape Town	Urban	Secondary hospital	Primary Literature (peer reviewed publications)	To examine the impact of multidisciplinary stroke care on the in-hospital mortality, resource utilization, and access to inpatient rehabilitation facilities for stroke patients admitted in Stroke units at a secondary hospital in Cape Town, South Africa	Cross-sectional pre and post study design	195 stroke patients
19	De Villiers 2011	Cape Town	Urban	District hospital	Primary Literature (peer reviewed publications)	To determine survival, disability and functional outcomes of stroke patients following their discharge from an acute stroke unit in an urban community with limited rehabilitative resources	Cross-sectional pre and post study design	196 stroke patients
20	Elloker (2015)	Western Cape	Urban	CHC	Dissertations	To determine participation restrictions and social support in patients with stroke, living in the Western Cape.	Mixed methods (Systematic Review + Quantitative survey)	106 stroke patients
21	Faux (2006)	Non-specific	Undefined	Undefined	Primary Literature (peer reviewed publications)	To provide a practical guide to helping stroke survivors who have a persistent disability maintain and enhance the gains made in rehabilitation	Narrative review recommendation	NA
22	Felemengas (2005)	Johannesburg Gauteng	Urban	Academic hospital	Dissertations	To investigate the family dynamics within the family system, as well as how these have evolved or changed following a stroke.	Qualitative	6 primary caregivers of stroke survivors
23	Groenewald & Rhoda 2017	Western Cape	Urban	Non-Governmental facility	Primary Literature (peer reviewed publications)	To determine outcomes of stroke patients managed by a multidisciplinary team at a step-down facility in the Western Cape.	A longitudinal observational study	68 stroke patients
24	Groenewald (2018)	Western Cape	Urban	Step down rehabilitation facility	Dissertations	To adapt and contextualize the original UK Bridges stroke SMI workbook for implementation with the South African stroke	A qualitative exploratory study -Interview, FGD, Expert	13 Health care professionals<br>12 Stroke patients<br>Expert Panel

For peer review only

						population	consultation	
25	Hassan et al (2011)	Western cape	Urban	Western Cape Rehabilitation centre	Primary Literature (peer reviewed publications)	To explore levels of strain experienced by caregivers and the variables that impact on their strain.	Concurrent, mixed method, descriptive design	57 caregivers of stroke survivors
26	Hilton (2011)	Johannesburg	Urban	Community	Dissertations	To establish the functional level of patients, the level of strain and quality of life of the caregiver six to 36 months post-stroke, and the influence of demographic factors, caregiver strain and patient's functional ability on quality of life of the caregiver.	Cross-sectional study	35 stroke patients and their caregivers
27	Hossain (2016)	Kwa-Zulu-Natal	Urban	Ladysmith Regional Hospital	Dissertations	To investigate the Need for Palliative care in Cerebrovascular Accident (stroke) patients at Ladysmith Regional Hospital	Mixed Methods (qualitative and quantitative)	72 stroke patients for quantitative study and<br>10 stroke patients for qualitative study
28	Joseph 2012	Western cape	Urban	WCRC – Rehabilitation centre	Dissertations	To determine the process of rehabilitation and the outcome of patients following in-patient rehabilitation at a facility in the Western Cape	A descriptive, observational, longitudinal design	76 Spinal Cord Injury patients and 67 stroke patients. Total patients (including drop outs) 130.
29	Kleineibst (2007)	Western Cape	Urban	Community	Dissertations	To determine the effectiveness of a caregiver support intervention programme to address the need for primary caregivers of stroke survivors in Bishop Lavis Community	Prospective descriptive qualitative study	29 caregivers of stroke survivors
30	Kotsokoane et al (2018)	Gauteng	Urban	CHC	Primary Literature (peer reviewed publications)	To determine the level of integration of stroke survivors at Soshanguve community clinics.	Retrospective quantitative research design	114 stroke survivors
31	Kusambiza-Kiingi (2016)	Johannesburg Gauteng	Urban	CHC	Primary Literature (peer reviewed publications)	To determine stroke survivors' levels of community reintegration, quality of life (QOL),<br>satisfaction with the physiotherapy services and the	Cross-sectional study	108 stroke survivors and 45 caregivers

For peer review only

						level of caregiver strain at community health centres within the Johannesburg		
32	Leichtfuss (2009)	Western Cape	Urban	Private acute care hospitals	Dissertations	To examine the practice of doctors with regards to stroke rehabilitation in private acute-care hospitals private acute-care hospitals and to evaluate information shared between doctors and pts i.r.t prognosis, severity, discharge, referral, timing of discharge planning and decision making. in the Western Cape Metropole	Retrospective and descriptive design	37 doctors treating and discharging stroke patients<br>48 pts
33	Mabunda (2015)	Western Cape	Urban	Private not for profit Intermediate care Facility	Dissertations	To describe the model of service provision at an IC facility and the role it plays in the continuity of care in Cape Town.	Cross sectional survey	68 stroke patients<br>70 clinical staff
34	Makganye (2015)	Gauteng?	Urban	CHC	Dissertations	To investigate the physical, psychological, social, religious	Qualitative design	5 stroke patients and 5 caregivers
35	Maleka et al (2012)	Limpopo and Gauteng	Urban and rural	Community	Primary Literature (peer reviewed publications)	To establish the experience of people living with stroke in low socioeconomic urban and rural areas of South Africa	Qualitative study design	32 stroke survivors living in the community
36	Mamabolo et al (2008)	Gauteng	Urban	PHC clinics	Primary Literature (peer reviewed publications)	to establish what demographic, environmental and physical factors influence functional independence post stroke.	Cross-sectional study	68 stroke patients
37	Mandizvidza (2017)	Western Cape	Urban	Level 1 2 3 hospitals	Dissertations	To describe the acute and post-acute ischaemic stroke services offered to ischaemic stroke patients in level 1, 2, and 3 hospitals in the Cape Metro Health District, compare these services to the national guideline and identify any barriers to optimum stroke	Descriptive cross-sectional study	10 doctors and 10 nurses from stroke ward and 8 doctors from emergency ward; pt records

For peer review only

						patient care.		
38	Mashau et al 2016	Limpopo	Rural and Urban	HBC organization	Primary Literature (peer reviewed publications)	To investigate the impact of caregiving on voluntary home-based caregivers.	A quantitative cross-sectional descriptive survey	190 home-based caregivers
39	Masuku et al (2018)	Gauteng	Urban	Community	Primary Literature (peer reviewed publications)	To describe the caregiving experience of female caregivers of PWA residing in Tembisa, a township situated in the east of Johannesburg	Qualitative study	14 primary caregivers of stroke survivors with Aphasia
40	Matshikiza (2019)	Western cape	Urban	Tertiary Hospital	Dissertations	to determine the pre-hospital barriers and in-hospital delays to emergency care for patients presenting to Groote Schuur Hospital (GSH) with acute stroke.	prospective, observational study	50 patients with stroke
41	Mudzi (2010)	Gauteng	Urban	Community	Primary Literature (peer reviewed publications)	To establish the impact of caregiver education on the morbidity of the stroke survivors and on the quality of life of the stroke survivors and their carers.	A stratified randomised controlled trial	200 stroke patients and caregivers
42	Mudzi et al (2013)	Gauteng	Urban	Community	Dissertations	to establish the extent of community participation and the barriers and facilitators to the participation for stroke patients after their discharge.	longitudinal study	200 patients with first-time ischaemic stroke
43	Ntamo (2011)	Eastern Cape	Urban	Mthatha General Hospital	Dissertations	To identify factors that influence poor attendance for outpatient physiotherapy by patients discharged from MGH with a stroke.	Mixed methods (Qualitative + Quantitative study)	85 stroke patients attending Physiotherapy at MGH
44	Parekh and Rhoda (2013)	Western Cape	Urban	Tertiary hospital	Primary Literature (peer reviewed publications)	To determine functional outcomes and factors influencing functional outcomes of stroke patients admitted to a South African tertiary hospital	Longitudinal Pre and Post test design	100 stroke patients
45	Parekh	Western	Urban	Tertiary	Dissertations	to identify factors influencing	A descriptive,	66 stroke patients

For peer review only

	(2011)	Cape		hospital		functional outcome of stroke patients admitted to a South African tertiary hospital	observational, longitudinal quantitative study design	
46	Posner (2015)	Gauteng	Urban	Community	Dissertations	to explore the experiences and perceived needs of employed caregivers working for patients who have suffered from a stroke within home settings in South Africa.	Qualitative research design with Interviews + FGDs	15 employed caregivers working at the homes of stroke survivors FGDs with 10 participants 5 in each group
47	Ras (2009)	Western Cape	Urban	NGO Run Hospital booth	Dissertations	to assess the quality of the stroke rehabilitation services at Booth Memorial Hospital.	Cross-sectional audit of records	NA
48	Rhoda (2009)	Western Cape	Peri - Urban	CHC	Primary Literature (peer reviewed publications)	to determine the structure and process of rehabilitation of stroke patients at Community Health Centres (CHCs) in the Western Cape	Quantitative cross-sectional survey	100 first time stroke patients and 16 therapists
49	Rhoda et al (2011)	Western Cape	Peri - Urban	CHC	Primary Literature (peer reviewed publications)	to determine the activity limitations of stroke patients receiving rehabilitation at out-patient Community health Centres (ChCs)	Longitudinal observational study	100 patients with stroke
50	Rhoda (2014)	Western Cape	Peri - Urban	CHC	Primary Literature (peer reviewed publications)	to determine the quality of life and factors influencing quality of life of community-dwelling stroke patients living in low-income, peri-urban areas in the Western Cape, South Africa.	Observational, longitudinal study	100 first time stroke patients
51	Rhoda et al (2015)	South Africa (Eastern Cape) Tanzania and Rwanda	Undefined	Provincial hospital	Primary Literature (peer reviewed publications)	the provision of inpatient rehabilitation and the post discharge challenges of stroke survivors in specific African countries.	Retrospective survey and interviews	168 SA stroke patients 145 Tanzanian and 130 Rwandan stroke patients 9 SA patients, 10 TP and 10 RP for qualitative study
52	Rouillard et al (2012)	Western Cape	Urban	WCRC rehab centre	Primary Literature (peer reviewed publications)	To determine activity limitations, participation restrictions, health-related quality of life and caregiver strain in community-dwelling	Longitudinal and descriptive study	46 stroke patients 41 caregivers

For peer review only

						stroke survivors discharged from an intensive inpatient rehabilitation programme at 6 months post stroke.		
53	SA-CSRG 2019	National	NA	NA	Primary literature (Peer-reviewed, Clinical Guideline)	contextualised development of stroke rehabilitation guideline	Guideline	NA
54	Scheffler and Mash 2019	Western Cape	Rural	Community	Primary Literature (peer reviewed publications)	To describe and analyze the outcomes of patients with stroke from a rural PHC setting in the Western Cape, South Africa.	Longitudinal survey	93 stroke patients
55	Smith (2019)	Western Cape	Western Cape	Urban and Rural	Community	To explore the self-management strategies employed by stroke survivors in the Western Cape, South Africa	Exploratory qualitative design (In-depth Interviews)	14 stroke survivors
56	Taylor & Ntusi (2019)	South Africa	South Africa	Undefined	Undefined	To improve management of stroke in South Africa	Editorial (Review)	NA
57	Thomas & Greenop (2008)	Gauteng	Gauteng	Urban	Community	To investigate into the complexities of caregiving, including both perceptions and experiences of the healthcare system.	Qualitative design (interviews)	6 caregivers of stroke survivors
58	Viljoen (2014)	Western Cape	Western Cape	Urban	Groote Schuur Hospital	To determine the cost of stroke care and to identify factors associated with increased expense, as well as to evaluate the quality of stroke care in general medical wards	Review of records	261 stroke patient (records)
59	Wasserman et al 2009	KwaZulu-Natal	KwaZulu-Natal	Rural	Community	To assess discharge planning of stroke patients and to evaluate integration and continuity of stroke care between hospital and community	Quantitative design (Survey)	30 stroke patients

For peer review only

Supplementary file S4: Components assessed in included studies

Author (year)	Governance /Regulation	Resources	Service Delivery	[illegible]	Re-organisation of care	Community engagement
Arowoiya (2014)			X	X		
Bham & Ross 2005			X	X		
Biggs (2005)		X	X	X		X
Biggs & Rhoda (2008)				X		
Blackwell & Littlejohns (2010)			X			
Botha (2008)					X	X
Bryer (2009)		X	X			
Bryer et al (2010)	X					
Burton 2016					X	X
Cawood 2012		X	X			X
Cawood& Visagie (2015)			X			X
Cawood & Visagie (2016)			X			X
Cawood et al (2016)						x
Connor (2005)	X	X		X		
Cunningham (2012)		X	X	X		X
Cunningham & Rhoda (2014)						X
De la Cornillère (2007)			X	X		X
De Villiers et al (2009)			X			
De Villiers (2011)		X	X			
Elloker (2015)						X
Faux (2006)					X	
Felemengas (2005)			X	X		X
Groenewald & Rhoda (2017)			X			
Groenewald (2018)			X		X	
Hassan et al (2011)				X		
Hilton (2011)			X	X		
Hossain (2016)			X		X	
Joseph (2012)			X			X
Kleineibst (2007)		X	X	X		
Kotsokoane et al (2018)			X			X
Kusambiza-Kiingi (2016)			X	X		
Leichtfuss (2009)		X	X			X
Mabunda (2015)			X			
Makganye (2015)		X	X	X		
Maleka et al (2012)		X		X		X
Mamabolo et al (2008)						X
Mandizvidza (2017)	X	X	X			
Mashau et al (2016)			X			
Masuku et al (2018)			X	X		
Matshikiza (2019)		X	X			
Mudzi (2010)			X	X		X
Mudzi et al (2013)			X	X		

Ntamo (2011)		X	X	X		X
Parekh and Rhoda (2013)			X			
Parekh (2011)			X			
Posner (2015)			X			
Ras (2009)		X	X			
Rhoda (2009)		X	X			
Rhoda et al (2011)			X			
Rhoda (2014)			X	X		
Rhoda et al (2015)			X	X		
Rouillard et al (2012)			X	X		
SA-CSRG	X					
Scheffler and Mash (2019)			X			
Smith (2019)			X	X		
Taylor & Ntusi (2019)			X			
Thomas & Greenop (2008)			X	X		
Viljoen (2016)		X	X			
Wasserman et al (2009)			X			
**Total**	**4**	**16**	**47**	**24**	**5**	**19**

\]

For peer review only

Preferred Reporting Items for Systematic reviews and Meta-Analyses extension for Scoping Reviews (PRISMA-ScR) Checklist

SECTION	ITEM	PRISMA-ScR CHECKLIST ITEM	REPORTED ON PAGE #
**TITLE**			
Title	1	Identify the report as a scoping review.	1
**ABSTRACT**			
Structured summary	2	Provide a structured summary that includes (as applicable): background, objectives, eligibility criteria, sources of evidence, charting methods, results, and conclusions that relate to the review questions and objectives.	2
**INTRODUCTION**			
Rationale	3	Describe the rationale for the review in the context of what is already known. Explain why the review questions/objectives lend themselves to a scoping review approach.	4
Objectives	4	Provide an explicit statement of the questions and objectives being addressed with reference to their key elements (e.g., population or participants, concepts, and context) or other relevant key elements used to conceptualize the review questions and/or objectives.	4
**METHODS**			
Protocol and registration	5	Indicate whether a review protocol exists; state if and where it can be accessed (e.g., a Web address); and if available, provide registration information, including the registration number.	6
Eligibility criteria	6	Specify characteristics of the sources of evidence used as eligibility criteria (e.g., years considered, language, and publication status), and provide a rationale.	6
Information sources*	7	Describe all information sources in the search (e.g., databases with dates of coverage and contact with authors to identify additional sources), as well as the date the most recent search was executed.	6
Search	8	Present the full electronic search strategy for at least 1 database, including any limits used, such that it could be repeated.	6
Selection of sources of evidence†	9	State the process for selecting sources of evidence (i.e., screening and eligibility) included in the scoping review.	6
Data charting process‡	10	Describe the methods of charting data from the included sources of evidence (e.g., calibrated forms or forms that have been tested by the team before their use, and whether data charting was done independently or in duplicate) and any processes for obtaining and confirming data from investigators.	6
Data items	11	List and define all variables for which data were sought and any assumptions and simplifications made.	6-7
Critical appraisal of individual sources of evidence§	12	If done, provide a rationale for conducting a critical appraisal of included sources of evidence; describe the methods used and how this information was used in any data synthesis (if appropriate).	7
Synthesis of results	13	Describe the methods of handling and summarizing the data that were charted.	7

St. Michael's
Inspired Care.
Inspiring Science.

For peer review only - http://bmjopen.bmj.com/site/about/guidelines.xhtml

SECTION	ITEM	PRISMA-ScR CHECKLIST ITEM	REPORTED ON PAGE #
**RESULTS**			
Selection of sources of evidence	14	Give numbers of sources of evidence screened, assessed for eligibility, and included in the review, with reasons for exclusions at each stage, ideally using a flow diagram.	7-17
Characteristics of sources of evidence	15	For each source of evidence, present characteristics for which data were charted and provide the citations.	7-17
Critical appraisal within sources of evidence	16	If done, present data on critical appraisal of included sources of evidence (see item 12).	7-17
Results of individual sources of evidence	17	For each included source of evidence, present the relevant data that were charted that relate to the review questions and objectives.	7-17
Synthesis of results	18	Summarize and/or present the charting results as they relate to the review questions and objectives.	7-17
**DISCUSSION**			
Summary of evidence	19	Summarize the main results (including an overview of concepts, themes, and types of evidence available), link to the review questions and objectives, and consider the relevance to key groups.	17
Limitations	20	Discuss the limitations of the scoping review process.	21
Conclusions	21	Provide a general interpretation of the results with respect to the review questions and objectives, as well as potential implications and/or next steps.	21
**FUNDING**			
Funding	22	Describe sources of funding for the included sources of evidence, as well as sources of funding for the scoping review. Describe the role of the funders of the scoping review.	22

JBI = Joanna Briggs Institute; PRISMA-ScR = Preferred Reporting Items for Systematic reviews and Meta-Analyses extension for Scoping Reviews.
* Where *sources of evidence* (see second footnote) are compiled from, such as bibliographic databases, social media platforms, and Web sites.
† A more inclusive/heterogeneous term used to account for the different types of evidence or data sources (e.g., quantitative and/or qualitative research, expert opinion, and policy documents) that may be eligible in a scoping review as opposed to only studies. This is not to be confused with *information sources* (see first footnote).
‡ The frameworks by Arksey and O'Malley (6) and Levac and colleagues (7) and the JBI guidance (4, 5) refer to the process of data extraction in a scoping review as data charting.
§ The process of systematically examining research evidence to assess its validity, results, and relevance before using it to inform a decision. This term is used for items 12 and 19 instead of "risk of bias" (which is more applicable to systematic reviews of interventions) to include and acknowledge the various sources of evidence that may be used in a scoping review (e.g., quantitative and/or qualitative research, expert opinion, and policy document).

From: Tricco AC, Lillie E, Zarin W, O'Brien KK, Colquhoun H, Levac D, et al. PRISMA Extension for Scoping Reviews (PRISMAScR): Checklist and Explanation. Ann Intern Med. 2018;169:467–473. doi: 10.7326/M18-0850.

For peer review only - http://bmjopen.bmj.com/site/about/guidelines.xhtml

BMJ Open

Towards universal health coverage for people with stroke in South Africa: a scoping review

Journal:	*BMJ Open*
Manuscript ID	bmjopen-2021-049988.R2
Article Type:	Original research
Date Submitted by the Author:	02-Oct-2021
Complete List of Authors:	Sjan-Mari, van Niekerk; Stellenbosch University, Division of Physiotherapy, Department of Health and Rehabilitation Sciences<br>Sureshkumar, K; Public Health Foundation of India, SACDIR; London School of Hygiene & Tropical Medicine, International Center for Evidence in Disability<br>Gakeemah, Inglis-Jassiem; Stellenbosch University, Division of Physiotherapy, Department of Health and Rehabilitation Sciences<br>Charumbira, Maria; Stellenbosch University Faculty of Medicine and Health Sciences, Rehabilitation Sciences<br>Fernandes, Silke; London School of Hygiene and Tropical Medicine,<br>Webster, Jayne; London School of Hygiene and Tropical Medicine,<br>English, Rene; Stellenbosch University Faculty of Medicine and Health Sciences, Global Health<br>Louw, QA; Stellenbosch University, Physiotherapy; Stellenbosch University,<br>Smythe, Tracey; London School of Hygiene and Tropical Medicine, Clinical Research
**Primary Subject Heading**:	Public health
Secondary Subject Heading:	Health policy, Health services research, Rehabilitation medicine, Research methods
Keywords:	Stroke < NEUROLOGY, PUBLIC HEALTH, Health policy < HEALTH SERVICES ADMINISTRATION & MANAGEMENT, Rehabilitation medicine < INTERNAL MEDICINE, PRIMARY CARE

SCHOLARONE™
Manuscripts

I, the Submitting Author has the right to grant and does grant on behalf of all authors of the Work (as defined in the below author licence), an exclusive licence and/or a non-exclusive licence for contributions from authors who are: i) UK Crown employees; ii) where BMJ has agreed a CC-BY licence shall apply, and/or iii) in accordance with the terms applicable for US Federal Government officers or employees acting as part of their official duties; on a worldwide, perpetual, irrevocable, royalty-free basis to BMJ Publishing Group Ltd ("BMJ") its licensees and where the relevant Journal is co-owned by BMJ to the co-owners of the Journal, to publish the Work in this journal and any other BMJ products and to exploit all rights, as set out in our licence.

The Submitting Author accepts and understands that any supply made under these terms is made by BMJ to the Submitting Author unless you are acting as an employee on behalf of your employer or a postgraduate student of an affiliated institution which is paying any applicable article publishing charge ("APC") for Open Access articles. Where the Submitting Author wishes to make the Work available on an Open Access basis (and intends to pay the relevant APC), the terms of reuse of such Open Access shall be governed by a Creative Commons licence – details of these licences and which Creative Commons licence will apply to this Work are set out in our licence referred to above.

Other than as permitted in any relevant BMJ Author's Self Archiving Policies, I confirm this Work has not been accepted for publication elsewhere, is not being considered for publication elsewhere and does not duplicate material already published. I confirm all authors consent to publication of this Work and authorise the granting of this licence.

For peer review only

Towards universal health coverage for people with stroke in South Africa: a scoping review

Sjan-Mari van Niekerk,[1] *Sureshkumar Kamalakannan ,[2,3] Gakeemah Inglis-Jassiem,[1] Maria Y Charumbira[1], Silke Fernandes,[4] Jayne Webster,[5] René English,[6] Quinette A Louw,[1] Tracey Smythe[3]

*Corresponding Author

Dr Sureshkumar Kamalakannan
International Centre for Evidence in Disability,
London School of Hygiene and Tropical Medicine, London, UK
suresh.kumar@lshtm.ac.uk

1 Division of Physiotherapy, Department of Health and Rehabilitation Sciences, Stellenbosch University, Cape Town, South Africa

2 Public Health Foundation of India, Indian Institute of Public Health Hyderabad, SACDIR, India

3 International Centre for Evidence in Disability, London School of Hygiene and Tropical Medicine, London, UK

4 Department of Global Health and Development, Faculty of Public Health and Policy, London School of Hygiene and Tropical Medicine, London, UK

5 Department of Disease Control, Faculty of Infectious and Tropical Diseases, London School of Tropical Health and Medicine, London, UK

6 Division of Health Systems and Public Health, Department of Global Health, Faculty of Medicine and Health Sciences, Stellenbosch University, Cape Town, South Africa

For peer review only

Total word count – 4566 without abstract, tables, figure, and references

Keywords – Stroke; Universal Health Coverage; Health System; South Africa; Scoping Review

For peer review only - http://bmjopen.bmj.com/site/about/guidelines.xhtml

ABSTRACT – word count 299

Objectives: To explore the opportunities and challenges within the health system to facilitate the achievement of Universal Health Coverage (UHC) for people with stroke (PWS) in South Africa (SA).

Setting: South Africa

Design: Scoping Review

Search Methods: We conducted a scoping review of opportunities and challenges to achieve UHC for PWS in SA. Global and Africa-specific databases and grey literature were searched in July 2020. We included studies of all designs that described the health care system for PWS. Two frameworks, the Health Systems Dynamics Framework and WHO Framework, were used to map data on governance and regulation, resources, service delivery, context, re-orientation of care, and community engagement. A narrative approach was used to synthesise results.

Results

Fifty-nine articles were included in the review. Over half (n=31, 52.5%) were conducted in Western Cape province and most (n=41, 69.4%) were conducted in urban areas. Studies evaluated a diverse range of health system categories and various outcomes. The most common reported component was service delivery (n=46, 77.9%), and only four studies (6.7%) evaluated governance and regulation. Service delivery factors for stroke care were frequently reported as poor and compounded by context-related limiting factors. Governance and regulations for stroke care in terms of government support, investment in policy, treatment guidelines, resource distribution, and commitment to evidence-based solutions were limited. Promising supporting factors included adequately equipped and staffed urban tertiary facilities, the emergence of Stroke units, prompt assessment by health professionals, positive staff attitudes and care, two clinical care guidelines, and educational and information resources being available.

Conclusion

This review fills a gap in the literature by providing the range of opportunities and challenges to achieve health for all PWS in SA. It highlights some health system areas that show

For peer review only

encouraging trends to improve service delivery including comprehensiveness, quality, and perceptions of care.

STRENGTHS AND LIMITATIONS OF THIS STUDY

- A comprehensive search strategy was developed, and the search was carried out in global, national, and continental-specific databases.
- The scoping review methodology included double data extraction and data review to synthesise the state of the evidence on the topic.
- The use of a combination of two frameworks, the Health Systems Dynamics and Integrated People-Centred Health Services contributed to rigorous evaluation.
- There was no limitation on study design or exclusion based on methodological appraisal for the inclusion of records.
- Comparison of studies was challenged by heterogeneity, especially regarding design and aim.

For peer review only

INTRODUCTION

Stroke is a leading cause of death and disability worldwide (1). In South Africa (SA) stroke is the second most common cause of death after HIV/AIDS and a significant cause of morbidity (2–5). It is estimated that 75 000 people experience a stroke each year in SA, contributing to 564 000 stroke-related disability-adjusted life-years (6). Furthermore, stroke incidence in rural areas of SA is increasing; an estimated 33,500 strokes occurred in these areas in 2011, contributing to half of the national stroke burden (7). However, these data are likely underestimated due to the absence of a national stroke database or registry and the paucity of studies that were undertaken in a few parts of the country.

Stroke is the leading cause of disability in adults in SA, placing strain on social and health services (8). Increased prevalence of heart disease, hypertension, diabetes mellitus, behavioural factors such as smoking, and structural factors such as unchecked industrialisation and urbanisation, contribute to this epidemiological transition of stroke in many low and middle-income countries (LMICs)(9), including SA (2). The SA government has committed to the World Health Organisation (WHO) vision of achieving equitable, evidence-based rehabilitation for all by 2030 (10). South Africa's Constitution guarantees every citizen to have access to health services (section 27 of the Bill of Rights). The SA health system comprises the public sector (the government managed) and the private sector. Public health services are divided into primary, secondary, and tertiary institutions managed by provincial Departments of Health, with the National Ministry of Health being responsible for policy development and coordination (11). Individuals can access either public or private health services, with access to private health dependant on an individual's ability to pay for services. The majority of South Africans (84%), access health services through government-run public clinics and hospitals (12). SA, stroke care, including rehabilitation, occurs across a range of settings, from tertiary hospitals to remote community primary healthcare facilities, and can be provided individually or in a group setting, at home, in a community environment, or a specialist centre (2). Whilst public health policy in SA ascribes to primary health care and a decentralised approach, many stroke care, and rehabilitation services remain centralised at district and specialist rehabilitation hospitals (13). It is not clear how many people access rehabilitation services following stroke, what this rehabilitation entails, and how effective this rehabilitation is (14). Therefore, achieving key global health targets and development goals will be challenging, including Universal Health Coverage (UHC) (15,16).

For peer review only - http://bmjopen.bmj.com/site/about/guidelines.xhtml

UHC is achieved when every person receives essential services, such as health promotion, prevention, treatment, rehabilitation (including assistive technology), and palliative care, according to their needs and without financial hardship (17). Accessible, responsive, and quality stroke care services within a strengthened local health system will contribute to UHC for people with stroke (PWS) in SA. The extent to which UHC is currently achieved for PWS in SA is unknown (18). We aimed to describe the health system-related factors that will facilitate UHC for PWS and the shortcomings that currently limit the implementation of UHC for stroke care in SA.

METHODS

A scoping review was conducted according to the five-step approach recommended by Levac et al.(19) as outlined in our published protocol (20): 1) identifying the research question, 2) identifying relevant studies, 3) selecting the studies, 4) charting the data, and 5) collating, summarising and reporting the results. The results are reported according to the Preferred Reporting Items for Systematic Reviews and Meta-Analyses extension for Scoping Reviews (PRISMA-ScR) guidelines (21).

Patient and public involvement

No patients and/or public were involved in the design, conduct, reporting, or dissemination plans of this research.

Analytical framework

This review was guided by an analytical framework adapted from the Health Systems Dynamics Framework (HSDF)(22) and WHO Framework on integrated people-centred health services (IPCHS)(23). Our analytical framework includes all the HSDF components and two components from the IPCHS: 1) Re-orientation of care and 2) Enabling environment, which is appropriate to the SA context and population (Figure 1). 'Resources' and 'Enabling environment' were combined and titled 'Resources' as the data items described under each were similar.

For peer review only - http://bmjopen.bmj.com/site/about/guidelines.xhtml

For peer review only

Figure 1: Analytic framework for health system-related factors that limit or support UHC, incorporating components from the Health Systems Dynamics Framework (22) and WHO Framework on Integrated People-Centred health Services (23).

Identifying the research question

To answer the question 'what are the opportunities and challenges within the SA health system to facilitate achieving UHC for people with stroke?' the review objectives were to:

1. describe the health system-related factors that support and guide achieving universal stroke care in SA
2. describe the health system-related factors that limit achieving universal stroke care in SA
3. identify driving factors with the potential to bring change required to achieve universal stroke care in SA

Identifying relevant studies

In line with the purpose of scoping reviews, our approach was broad, with emphasis on studies that investigated any aspect of the health care system regarding stroke care in SA.

Search Strategy

We conducted a comprehensive search, according to the methodology described in our published protocol (20) and an example of the search strategy is available as a supplementary file (S1). Grey literature was identified through the National Electronic Thesis and Dissertation portal, and websites of relevant government and service provider agencies. Field experts were contacted to identify additional relevant evidence regarding stroke care in SA. Saturation was the point at which no new records were found for inclusion.

Eligibility criteria

Full text, SA-based studies on stroke care of any design that addressed at least one framework component were included (20).

Evidence selection

Two reviewers (SvN, SK) independently screened the titles and abstracts of identified studies. A third reviewer (GIJ) checked the results for accuracy. Results of the initial screening were compared, and full-text records were obtained for articles deemed eligible by at least one reviewer. Two reviewers (SvN, SK) independently screened the full texts using

For peer review only - http://bmjopen.bmj.com/site/about/guidelines.xhtml

the eligibility criteria. Any discrepancies were resolved by discussion with a third reviewer (GIJ). Data were managed with Covidence (https://support.covidence.org/help) and Excel (version 365).

Data charting

The six framework components were divided between three reviewers (SvN; SK; MC) who extracted, collated, and summarised relevant data into a purpose-built Microsoft Excel database. We considered the six components using the descriptions as outlined in Supplementary file 2 (S2) and data on the following study components were extracted:

- General study information, including author and year of publication
- Study design, sampling, and recruitment methods
- Study settings and dates conducted
- Population characteristics
- Study measures
- Research outcomes related to the framework components

The three reviewers compared their results and reached a consensus on the organisation of extracted data. The final data and analysis were evaluated by a research team member (TS), to ensure that interpretations were credible and valid.

Data synthesis and analysis

We summarised the study characteristics and the study designs. We used a framework analysis approach to deductively analyse data of the included studies, which consisted of five key steps as described by Ritchie et al. (24). The framework in Figure 1 was used as a dynamic tool to aid this synthesis and data was managed with Atlas.ti (version 8) and Microsoft Excel (version 365).

The final synthesis of themes was confirmed following a critical discussion between all the authors. We undertook a narrative synthesis of the findings, highlighting supporting and limiting factors to achieving UHC for PWS in SA. The range of opportunities and challenges to achieve health for all PWS in SA was synthesised and included in the framework diagram.

RESULTS

For peer review only - http://bmjopen.bmj.com/site/about/guidelines.xhtml

We identified a total of 4,133 records and screened the abstracts of 508. After reviewing 75 full-text records, we included a total of 59 full texts in our review. A PRISMA flow diagram summarised the study selection process (Figure 2).

Figure 2: PRISMA flow chart

Study characteristics

The majority (n=41, 69.4%) of studies were conducted in urban areas, and over half of them (n=31, 52.5%) were undertaken in the Western Cape province. No studies were found from four of the nine provinces in SA (Free State, Mpumalanga, Northern Cape, or the northwest Provinces). The most common study design was quantitative (n=22, 37,2%), followed by mixed methods (n=14, 23.7%) and qualitative (n=10, 16.9%). Eighteen (30%) studies were community-based whilst the remaining studies recruited participants from clinics (n=12, 20.3%) or hospitals (n=16, 27.1%). The most commonly reported framework component was Service Delivery and (n=46, 77.9%) and the least reported was Governance and Regulation (n=4, 6.7%) (Table 1).

Supplementary file 3 (S3) provides a detailed summary of included records and Supplementary file 4 (S4) provides information on components reported per included record.

Variable	Category	N (%)
Province	Western Cape	31 (52.5%)
	Gauteng	12 (20.3%)
	National	6 (10.1 %)
	Eastern Cape	4 (6.7%)
	KwaZulu-Natal	2 (3.3%)
	Limpopo	1 (1.6%)
	Limpopo and Gauteng	1 (1.6%)
	Free State	0 (0%)
	Mpumalanga	0 (0%)
	Northern Cape	0 (0%)
	North West	0 (0%)
	Undefined	2 (3.3%)
Area	Urban	41 (69.4%)
	Rural and Urban	3 (5.0%)
	Peri-Urban	3 (5.0%)
	Rural	2 (3.3%)
	Undefined	10 (16.9%)
Levels of care	Community	18 (30.5%)
	Hospital	16 (27.1%)
	Primary Healthcare (Clinics; Community Health Centres)	12 (20.3%)

For peer review only - http://bmjopen.bmj.com/site/about/guidelines.xhtml

For peer review only

	Rehabilitation centres	6 (10.1%)
	Undefined	7 (11.8%)
Study design	Quantitative	28 (47.4%)
	Mixed methods	14 (23.7%)
	Qualitative measures	10 (16.9%)
	Review	2 (3.3%)
	Editorial	3 (5.0%)
	Guideline	2 (3.3%)
Record description	Primary Literature(publications)	34 (57.6%)
	Grey Literature: Dissertations	25 (42.3%)
Included population	PWS	34 (57.6 %)
	Editorials and reviews	8 (13.5%)
	Caregiver	6 (10.1 %)
	PWS + Caregiver	5 (8.4 %)
	PWS + HCP	3 (5.0 %)
	HCP	1 (1.6 %)
	PWS + HCP+ Experts	1 (1.6 %)
	Traditional healers + Caregivers	1 (1.6 %)
	Policy makers	0 (0 %)

PWS = people with stroke; HCP = health care provider

Table 1: Characteristics of included records (N=59)

Twenty-one articles (35.5%) reported on a single framework component, of which Service Delivery (n=12/21, 57.1%) was the most commonly described. The majority of articles included a combination of components (n=38, 64.4%); 24 articles (40.6%) reported on two framework components, and fourteen articles (23.7%) reported on three or more. Of the combination of components, Context was most commonly combined with Service Delivery (n = 11/38, 28.9%) followed by Resources and Service Delivery (n=5/38, 13.1%).

Service Delivery

Comprehensiveness

A comprehensive multi-disciplinary team (MDT), defined as consisting of five or more different types of health care professionals working together in a coordinated manner, was reported in nine studies (25-33). Two studies indicated that MDTs were either absent, limited, or inefficient (34,35).

For peer review only - http://bmjopen.bmj.com/site/about/guidelines.xhtml

Continuity of Care

Continuity of care was limited by poorly defined referral pathways, bed capacity for inpatient care, coordination of care and communication (among healthcare providers and with patients) in regards to care and discharge planning as well as follow-up systems. One study indicated that poor understanding of faith-based medicine by medical professionals and reciprocal lack of trust between medical and faith-based medicine practitioners may hinder adequate stroke care (36). At the community level, referral to support groups lacked coordination and stroke survivors lacked knowledge of care options (29,37–39). Two studies conducted in a rural part of the Western Cape reported that 30% (n=19) of 64 patients referred for home-based care, did not receive rehabilitation from community health workers following an assessment and treatment plan designed by a district therapist. Long waiting times contributed to a paucity of therapy sessions. Those who did receive therapy had a median of three visits that lasted 20 minutes each (2,40). Waiting time for investigations such as magnetic resonance imaging or computerised tomography scans and general stroke care was lengthy (34,38,41). Consequently, delays in investigations were found to be associated with a significant increase in length of stay (42) and doctor-led models, where a doctor is solely responsible for the patient's care and flow of information, leading to delays in investigations and/or treatments (40,43,44).

Timeliness of Care

Bed shortages (30,35,38,41,45) resulted in pressure to discharge patients from hospitals, which precluded rehabilitation and delayed post-discharge rehabilitation (31,35,46,47). In addition, doctor-led models of care were reported to lead to delays in care as staff waited for instruction or referral from a doctor before conducting investigations or administering treatment (31,37). Four studies (31,46–48) reported that patients were discharged when medically stable (average stay was 5-10 days at secondary or tertiary hospitals (30,39,46,49) despite functional deficits (29,30,46,50,51). Cunningham (2012) (47) reviewed 168 stroke patient acute care records from the Eastern Cape province and found only 15% were referred for physiotherapy on the day of or a day before discharge (47). Over weekends, 13% of acute-care patients did not receive any therapy (47). Difficulty with securing follow-up appointments and cancellations influenced the timeliness of post-discharge care (2,34,40,52,53).

For peer review only - http://bmjopen.bmj.com/site/about/guidelines.xhtml

Quality of Care

Four studies conducted in the Western Cape found that patients received between one and five rehabilitation sessions during acute care in hospital, except for the specialised sub-acute, in-patient Rehabilitation Centre where patients typically received 17 sessions (28,31,54-55). Length of stay was typically 5 - 10 days and approximately 30 days in rehabilitation facilities (42,56–59). One study reported that prompt assessment by rehabilitation professionals was associated with a shorter length of stay (42).

Perceptions of Care

There was conflicting evidence regarding perceptions of care. Ten studies reported positive staff attitudes (32,34,40,44,59–64) while nine studies reported negative staff behaviour and attitudes (33,36,40,52,60,65–68). A further four studies found that PWS were dissatisfied with the healthcare service along the entire continuum of care, which was driven by a lack of information about their treatment and further referral (34, 36,68). Leichtfuss (2009) (33) highlighted the significant discrepancy (p-value = 0.00438) between doctors' understanding and patients' perception of the effectiveness of the doctors' communication; 80% (n=28) of doctors compared to 50% (n=24) of patients thought that sufficient information was communicated (33). The study also found that patients perceived nursing services as inefficient and inadequate, which was supported by doctors who expressed the need for nursing staff who were trained in stroke care (33). Caregiver support and training were found to be lacking (39,65,67,69) and resulted in caregiver burnout (66). Caregivers indicated the need for additional training and help, particularly with toileting and bath transfers, and requested more home visits by therapists (39). Table 2 outlines measures and study findings that target Service Delivery.

Table 2: Supportive and limiting factors influencing different components of Service Delivery (N=46).

	**Service Delivery**	**Source of evidence: Author (year)**
**Comprehensiveness of Care**		
Facilitators	Comprehensive multi-disciplinary teams consisting of five or more different healthcare professionals in Western Cape province	Groenewald and Rhoda (2017); Rhoda et al. (2015); Joseph (2012); Rouillard et al., (2012); Leichtfuss (2009) Ras (2009); Wasserman et al, (2009); Rhoda (2009); De la Cornillère (2007)

For peer review only - http://bmjopen.bmj.com/site/about/guidelines.xhtml

Barriers	Limited/absent multi-disciplinary team consisting of less than five different healthcare professionals	Cawood (2012); De Villiers (2011)
**Continuity of Care***		
Barriers	Poor referral pathways (community; hospital)	Masuku (2018); Mandizvidza (2017); Cawood & Visagie (2016); Joseph (2012); De la Cornillère (2007); Kleinheibst (2007)
	Poor follow-up and referral post-discharge	Rhoda (2014); Rouillard (2012); Bham & Ross (2005); Scheffler and Mash (2019);
	Lack of reciprocal respect and understanding and coordination between traditional and medical healthcare professionals	Bham & Ross (2005)
**Timeliness of Care***		
Barriers	Long queues in hospitals, community health clinics, and outpatient clinics	Cawood (2012); Mudzi (2013)
	Long waiting times for follow-up appointments	Arowoiya (2014)
	Long waiting times for inpatients to receive specialised health services	Matshikiza (2019); Mandizvidza (2017); Parekh & Rhoda (2013); Cawood (2012); Bryer (2009)
	Doctor-led model of care	Cawood & Visagie (2015); Cawood (2012)
	Poor collaboration between health care providers	Cawood (2012); Parekh (2011)
	Inadequate/no rehabilitation during hospital stays	Cunningham (2012); Hilton (2011); De Villiers (2009); Rhoda (2009)
**Quality of Care**		
Facilitators	Prompt assessment by an allied health professional significantly decreases the length of stay	Viljoen (2014)
Barriers	Lack of appropriate care due to lack of stroke-specific knowledge	Mandizvidza (2017); Leichfust (2009); Ras (2009)
	Insufficient number of in-patient rehabilitation sessions	Groenewald & Rhoda (2017); Parekh (2011) ; Rhoda et al (2011);Rhoda (2009)
	Short length of stay at all levels of care except for specialist rehabilitation facilities	Groenewald (2018); Mabunda (2015); Rhoda (2014); Viljoen (2014); Hilton (2011); Parekh (2011); Blackwell & Littlejohn (2010); Mudzi (2010); Ras (2009); Kleinhebst (2007); Felemengas (2004)

For peer review only - http://bmjopen.bmj.com/site/about/guidelines.xhtml

**Perceptions of Care**		
Facilitators	Positive staff attitudes and care	Taylor & Ntusi (2019); Groenewald (2018); Kotsokoane (2018); Hossain (2016); Kusambiza-Kiingi (2016); Cawood & Visagie (2015); Bham & Ross (2005); Cawood (2012); Ntamo (2011); De la Cornillère (2007)
Barriers	Negative staff attitudes and behaviour e.g., impersonal care; inappropriate support; poor communication; lack of cultural sensitivity, rudeness, and delayed assistance with patient's personal hygiene	Smith (2019); Cawood & Visagie (2015); Makganye (2015); Posner (2015); Arowoiya (2014); Leichtfuss (2009); Thomas & Greenop (2008); Bham & Ross (2005); Biggs (2005)
	Dissatisfaction with health care received	Arowoiya (2014) ; Cawood (2012); Bham & Ross (2005) Ntamo (2011); Kleineibst (2007)
	Lack of caregiver training	Kusambiza-Kiingi (2017); Mashau et (2016); Mudzi (2010); Kleineibst (2007); Rouilliard (2012); Felemengas (2004)

*No supporting factors reported.

Resources

Infrastructure

A mixed-method study by Ntamo et al.(63) reported that substantial traveling distances were required to access rural healthcare facilities. This was echoed in Bryer's editorial on the need for community-based stroke care (45). Makganye et al.(60) reported that 71% of 85 rural patients (n = 60) lived over 25 km away from their nearest hospital (60). Furthermore, more specialised services often remained inaccessible (30,31,45) as their geographic location required even longer travel times. Physical access for people with a disability was further limited by poor building infrastructure (e.g. no ramps, vast distances between departments) or/and uneven terrains (70).

Three articles (longitudinal study, cross-sectional study, and editorial) reported a lack of diagnostic equipment in rural facilities (26,38,45) in contrast with well-resourced urban facilities. (30,31). . A mixed-methods study (63) and editorial by Taylor and Ntusi's 2019 s editorial reported frequent stock-outs of basic medication at the primary care level, which resulted in additional expenses and patients' reluctance to return to rural clinics.

For peer review only - http://bmjopen.bmj.com/site/about/guidelines.xhtml

Human Resources

Adequately equipped urban rehabilitation centres were described in two studies (30; 31). Six studies found that high bed demand and rehabilitation workforce shortages led to high healthcare provider workloads (30,31,34,38,45,60). Therapists reportedly treated 2-3 times more patients than the daily recommendation (30). Mandizvidza (2017) (38) reported that nursing shortage at all healthcare levels in rural KwaZulu Natal negatively impacted basic stroke care. However, better-resourced urban tertiary hospitals in the Western Cape were also reported to experience staff shortages (38). A quantitative cross-sectional study found that rehabilitation services are severely limited at the primary care level with half of the community health centres in the Western Cape providing rehabilitation services, and only two offering speech therapy (31). Stroke care was often provided by healthcare professionals without specific stroke-related training (30,33,38) (Table 3).

None of the included articles reported on financial allocations for stroke care.

Table 3. Facilitators and barriers influencing different components of Resources (n = 16)

	**Resources**	**Source of evidence: Author (year)**
**Infrastructure**		
Facilitators	Adequate equipment (urban rehabilitation centre setting)	Ras (2009); Rhoda et al (2009)
Barriers	Lack of equipment (rural setting)	Mandizvidza (2017); Cawood (2012); Cunningham (2012); Rhoda et al (2009)
	Inadequate number of ambulances; ineffective systems to request an ambulance	Mandizvidza (2017); Biggs (2005)
	Poor accessibility of health centres due to location, building structure, or terrain surrounding the health facility	Maleka (2012); Ntamo (2011); Bryer (2009); Rhoda (2009)
	Insufficient number of beds or hospitals due to fiscal problems	Matshikiza (2019); Mandizvidza (2017); De Villiers (2011); Bryer (2009); Ras (2009)
	Inadequate special investigations and	Mandizvidza (2017); Viljoen (2014)

For peer review only - http://bmjopen.bmj.com/site/about/guidelines.xhtml

	infrastructure for diagnosis and management	(2016); Bryer (2009)
	Frequent medication outages	Taylor & Ntusi (2019); Ntamo (2011)
**Human Resources** *		
Barriers	Staff shortages	Mandizvidza (2017); Makganye (2015) ; Cawood (2012); Bryer (2009), Ras (2009); Connor (2005)
	Lack of stroke-care specific training for staff	Mandizvidza (2017); Leichfust (2009); Ras (2009); Kleineibst (2007)

*no supporting factors reported

Context

Wellbeing and caregiver factors

Two longitudinal studies and one retrospective survey reported mental health problems such as anxiety and depression among PWS and caregivers (26,27,71). PWS also described feelings that related to confinement, personality changes, imposed family adjustments, and caregiving burden (50,57,72). Gender bias in caregiving roles was reported where women commonly left employment to assume caregiving responsibilities of male partners or parents (46) or children cared for women with stroke (47,57,70).

Financial implications

Financial burden was found to increase when spouses became primary caregivers (without gainful employment) or through the employment of additional caregivers (57). Costs post-stroke were high due to additional caregiving expenses (60,73) and studies found that there was limited access to disability-, old age- or child-support grants (52,65). The financial burden among rural stroke survivors was compounded by low income before the stroke, difficulty in obtaining social grants due to limited awareness of eligibility criteria and the application processes, and lack of transport to submit grant applications (53,66). Poverty impacted access and utilisation of rehabilitation as available finances were preferentially used to meet basic needs such as food (74).

For peer review only - http://bmjopen.bmj.com/site/about/guidelines.xhtml

Access to Transport

Six studies reported transport being a limiting factor to access care due to expensive private transport, unreliable public transport, and inflated costs of a trip to accommodate assistive devices (32,37,39,63,65,75).

Cultural beliefs and Health Literacy

Two qualitative case studies reported that PWS in SA held cultural beliefs regarding the cause and recovery of strokes, such as ascribing stroke to witchcraft or religious beliefs (36, 60). Poor health literacy (60,66,68) and these beliefs further affected the care-seeking ability of communities. Bham and Ross (2005) (36) reported that healthcare professionals needed greater awareness of cultural practices, such as the inclusion of extended family in decision-making procedures, adaption of communication style when interviewing older persons, and sensitivity to religious and traditional beliefs, to facilitate the inclusion and full participation of marginalised communities (36).

Community Engagement

Self-efficacy

Leichtfuss (2009) (33) found that PWS and/or their caregivers believed that they were not involved in decision-making with regard their care. Felemengas (2004) (57) and Cawood (2012) (34) reported that PWS were neither confident with self-health management nor satisfied with pre-discharge training and information (34,57). A large mixed methods study (65) that included a survey (N= 418) reported that PWS and caregivers lacked awareness of the availability and benefit of rehabilitation services or support groups and this was echoed by Burton's (2016) editorial (76). Cawood et al (43) found that nearly half (n=53; 47%) of the participants in their cross-sectional study indicated via a survey that they did not receive assistance from stroke organisations (40). Low participation in a peer support program was found (29) despite patients who attended stroke support reporting better self-efficacy and feeling supported (34,65).

Community Integration

People with stroke were not fully re-integrated into their communities (61,77) due to negative attitudes of family, friends, and society (34). Inaccessible community activities (28.3%), poor mental health (18.9%)(78), financial constraints (45.3%)(77), and inaccessible transport (65) contributed to limited community integration. Fear of stigmatisation (70), functional

For peer review only - http://bmjopen.bmj.com/site/about/guidelines.xhtml

dependency especially due to incontinence (32,37,50,63,79), and fear of becoming a victim of crime (40) also limited integration.

Homecare resources

A Stroke Home Care booklet (in different languages) was developed for the SA context (80). In focus group discussions, seven-stroke survivors (n=15; 46%) demonstrated improved knowledge, confidence, and ability to communicate information about their stroke after using the booklet (80). However, the sample included in this study was small and the booklet was only available in English when acceptability was tested.. The stories and pictures were found to be culturally sensitive (80).

Reorganisation of Care

Educational and information resources

Two educational resources were available via institutional websites for the public: The stroke Home Care booklet (80) and the SA contextualised Bridges Stroke Self-management intervention workbook (59). The MyStroke website (www.mystroke.co.za) was developed following a public health awareness campaign and lists available stroke care centres and services for better coordination (76). The mySOS app is an e-health initiative that directs and connects users with emergency care, potentially improving the timeliness of care. In rural settings, telemedicine was used to connect with specialist services (81). However, none of these resources included efficacy trials or determined the usage of the website or application.

Stroke Unit

At a central hospital in Western Cape, the stroke unit was associated with reduced mortality and increased rehabilitation referral, staff training, and family involvement in treatment decisions (48). Stroke units were recommended in evidence-based SA stroke care guidelines (13,82).

Palliative care integration

Findings based on focus groups of patients recommended that palliative care should be incorporated into stroke care. However, better education of all stakeholders on palliative care benefits was needed (44).

Governance and Regulation

For peer review only - http://bmjopen.bmj.com/site/about/guidelines.xhtml

Two-stroke clinical care guidelines for SA were identified (13,82). One focussed on acute and post-acute stroke care (82), and the other on stroke rehabilitation (13). Mandizvizda (2017) (38) evaluated the level of adherence to the acute stroke care guideline in all levels of care in the Western Cape province and reported poor adherence in primary, secondary, and tertiary hospitals (general wards), with the two Stroke Units (situated in tertiary hospitals), being the most compliant (38). Challenges to adherence of the guidelines included staff shortages, limited access to diagnostic investigations, and delays in patients presenting to healthcare facilities (38).

There were no national stroke-specific policies. Whilst many people with disabilities are reliant on financial support from the government through grants, there was no specific policy on financial support for PWS or their caregivers. Poor intersectoral coordination between government departments was found with regard the responsibility for policy concerning persons with disabilities (83). Governance and Regulations was the most limited component reported, which demonstrates a deficit in leadership and policy for how stroke care should be implemented and conducted at all levels of care.

Limiting and supporting factors

Health system limitations and factors that support the achievement of UHC for PWS in SA are presented in Figure 3. Findings of each health system component of the framework are mapped and identify challenges and opportunities that speak to stroke care in the public sector.

Figure 3: Limiting and supporting factors toward achieving UHC

DISCUSSION

This scoping review summarises the available evidence of achieving health for all PWS in SA. Included articles evaluated a diverse range of health system categories and various outcomes, with the majority of studies reporting on two or more framework components. There were several key limiting factors toward achieving UHC, which included a lack of governmental regulation in terms of stroke policies and guidelines poor timeliness of care, a lack of the continuity of care and a lack of a comprehensive multi-disciplinary team at rural health facilities. Furthermore, bed and staff shortages and a lack of stroke-specific training, poor access to acute care and diagnostic equipment contributed to limiting UHC. Regular

For peer review only - http://bmjopen.bmj.com/site/about/guidelines.xhtml

medication stockouts, lack of caregiver training and negative perceptions of care were also found to be important limiting factors. There were also many supporting factors toward achieving UHC for PWS in SA, which included adequately equipped and staffed urban tertiary facilities, the emergence of Stroke Units in urban areas, prompt assessment by rehabilitation professionals, and positive staff attitudes and care. Resources that were available to support achieving UHC include two clinical care guidelines, and educational and information resources being available online. Drivers to achieve UHC for PWS in SA may include better governance and regulation to mitigate fiscal shortfall that has resulted in infrastructure and human resource limiting factors, intersectoral collaboration between government departments to assist with access to social support, and reliable and affordable transport to access healthcare.

Limiting factors

A key finding of this review is a lack of adequate Governance and Regulations in terms of government support and investment in policy and treatment guidelines, resource distribution, and commitment to evidence-based solutions (e.g. stroke units). Equity for people with disabilities, including PWS, requires a concerted commitment from the SA government to ensure that UHC for all is achieved (84). Opportunities to facilitate these renewed efforts include administrative interventions by both government and hospital management to address system-based limiting factors, such as access to patients' medical records and obtaining appointments. Addressing staff shortages and improving stroke-specific training may mitigate the excessive workload of healthcare workers and improve service delivery, as was achieved during a pilot program in Namibia, where an increase in the number of nurses resulted in improved service delivery (85). However, attracting and retaining health professionals in rural and remote areas is multi-factorial (86,87) and contextual strategies to attract and retain health professionals in these areas are needed (86). Dedicated stroke units in hospitals have reduced stroke mortality, increased access to rehabilitation from multidisciplinary teams, and have resulted in improved discharge planning services at these stroke units compared to managing PWS in general medical wards (48). Political and leadership support for these units may contribute to better stroke outcomes and improve community re-integration and return to work for PWS in SA.

Service Delivery and Context related factors were most frequently reported in combination and were consistently reported as poor. Findings of poor timeliness of care, a lack of

For peer review only - http://bmjopen.bmj.com/site/about/guidelines.xhtml

For peer review only

continuity of care, and absence of comprehensive multidisciplinary teams in rural areas are similar to health system weaknesses found in Rwanda and Malawi (88). The main hindrances affecting service delivery in SA related to training, resources and communication channels. Poor referral networks and few rural rehabilitation facilities were compounded by inadequate caregiver training, lack of stroke-specific staff training, bed shortages, and diagnostic equipment. As a result, many PWS are lost to follow-up care leading to poor management of comorbidities and potentially placing patients at risk of recurrence and secondary complications such as spasticity, pressure sores, aspiration pneumonia, and mobility difficulties (89, 90).

Access to equitable and affordable health care for PWS may be affected by contextual factors outside the healthcare system. Social determinants of health (poverty, education) and general safety can be addressed through intersectoral collaboration. Social service and health sector collaboration may ensure that eligible PWS are aware and have access to social grant support. This was echoed as an international need in a scoping review which included studies from North America, the United Kingdom, and Europe (88). Cooperation between both private and public transport services, and the health sector is needed to find a solution for accessible, affordable, and reliable transport for PWS and their caregivers. Whilst there is strong evidence of the link between lack of access to transport and negative effects on health care, research on possible solutions and the effectiveness thereof is scarce.

Supporting Factors

Despite the many limiting factors that were described, there are promising supporting factors to achieve UHC for PWS in SA. Well-equipped rehabilitation facilities in urban areas, comprehensive multi-disciplinary teams in urban, tertiary hospitals and a stroke unit in an urban area are already in place. There were also two clinical care guidelines and educational and information resources were available. Although some PWS reported their dissatisfaction with the care they received several studies reported patient and caregiver satisfaction, as well as positive staff attitudes, which were perceived to facilitate physical improvement through rehabilitation compliance. This was consistent with findings where the attitude and emotional approach of health professionals, as well as caregivers, affected the level of motivation for rehabilitation attendance in PWS in an inner city teaching hospital in a high income setting (91). Maclean et al.(91) found that a positive rapport between patients and healthcare

For peer review only - http://bmjopen.bmj.com/site/about/guidelines.xhtml

providers resulted in increased motivation and easy transmission of information about rehabilitation.

Implications for future research

The limited supporting factors and a multitude of limiting factors reported in the included articles of this scoping review highlight the gaps that remain and present opportunities for future research. Key questions include the effect of continuity and timeliness of care, and perceptions of care on the improvement of service delivery, as well as the effect of resources (such as staffing, bed allocation and access to diagnostic equipment) and the impact of stroke-related training on service delivery. The distribution of research as reported in this review was found to be disproportionate with just over half of the studies being conducted in a single province (Western Cape) and largely in urban areas, with four of the nine provinces not being reported on at all. Insights regarding barriers and facilitators to UHC for PWS residing in these unreported provinces are warranted.

Future research may focus on:

- Strategies to coordinate care for multi-morbidity (e.g., combined appointments with different health professionals) to minimise financial hardship on healthcare users and to evaluate effective and efficient holistic management of health, compared to silo treatment approaches.
- Extension of research on stroke services in the under-reported provinces.
- Evaluation of accessible, quality services beyond urban areas.
- Development and testing of stroke-specific capacity development for staff that is evidence-based, patient-centered, and holistic. Factors to highlight in training may include cultural responsiveness and awareness of the social determinants of health.
- Strategies to improve and implement person-centred discharge planning, which should include caregiver training and support before and after discharge.
- Development and evaluation of sustainable strategies to provide peer support groups either in person or on a digital platform, for both PWS and their caregivers, to provide ongoing support.
- Innovative public health campaigns via social media, television, or radio to increase the awareness of stroke signs and the urgency of seeking help. The impact, reach, and process evaluation of such campaigns should monitor effectiveness.

For peer review only - http://bmjopen.bmj.com/site/about/guidelines.xhtml

Strengths and limitations of the scoping review

We used a comprehensive search strategy that followed PRISMA guidelines, and robust methods that included double data extraction and review to produce a comprehensive state of the evidence. Our framework for analysis included a people-centered framework that acknowledged that health service provision should be coordinated around people's needs and preferences and provided in a way that is safe, effective, timely, affordable, and of acceptable quality. The framework also acknowledged the political context and the social and economic determinants of health. However, this review has limitations. The disproportioned distribution of where research on stroke care services was conducted may have limited generalisability. We included research articles, dissertations, and commentaries, and may have missed evidence indexed in health or government websites.

CONCLUSION

Stroke is the leading cause of disability in adults in SA, which places strain on national social and healthcare services and the SA government has committed to the WHO vision of achieving equitable, evidence-based healthcare for all by 2030. However, his review highlights health system components such as Governance, that requires strengthening, to enhance readiness for UHC for PWS,

Despite the available guidance on the best strategies to support healthcare systems in delivering stroke care services, the main findings of this review show that the stroke care services for PWS in SA are limited with a strong urban bias. The findings of this review have highlighted health systems challenges that speak to inequitable stroke care in the public sector. Health system strengthening driven by good governance & regulation of health services, continuity and timeliness of care, accessible facilities and well-equipped rehabilitation services is urgently needed. Health system limitations are compounded by contextual factors, highlighting the need for health system strengthening strategies that are tailored for the local context.

This scoping review highlights some health system areas that show encouraging trends to improve service delivery including comprehensiveness, quality and perceptions of care. The results of this review can be used to inform policymakers and healthcare professionals of healthcare system challenges and opportunities to effectively move towards UHC for PWS in SA. Governments should be held accountable for stroke care in terms of financial resource

For peer review only - http://bmjopen.bmj.com/site/about/guidelines.xhtml

allocation, and prioritise this marginalised group in the proposed national health insurance scheme.

DECLARATIONS

Ethics and dissemination Ethical approval were not required for this scoping review, as it only included published and publicly available data. The findings of this review are published in an open-access, peer-reviewed journal and developed an accessible summary of the results for website posting and stakeholder meetings.

Contributors SvN and SK in consultation with all authors constructed the search. SvN, SK, and MC extracted all data in consultation with all authors. SvN, SK, GI-J, JW, QAL, and TS analysed the extracted data. SvN drafted and revised the paper. SK, GI-J, MC, SF, RE, JW, QAL, and TS reviewed the manuscript and provided feedback on the drafts. All authors read and approved the final manuscript.

Competing interests None declared.

Funding This research was commissioned by the National Institute for Health Research (NIHR) Global Health Policy and Systems Research Development Award using UK aid from the UK government. Grant number NIHR130180.

Data availability Statement All data relevant to the study are included in the article or uploaded as supplementary file

Disclaimer The views expressed in this publication are those of the author(s) and not necessarily those of the NIHR or the Department of Health and Social Care.

Patient consent for publication Not required.

For peer review only

For peer review only - http://bmjopen.bmj.com/site/about/guidelines.xhtml

REFERENCES

Bibliography

1. Naghavi M, Abajobir AA, Abbafati C, Abbas KM, Abd-Allah F, Abera SF, et al. Global, regional, and national age-sex specifc mortality for 264 causes of death, 1980-2016: A systematic analysis for the Global Burden of Disease Study 2016. Lancet. 2017

2. Scheffler E, Mash R. Surviving a stroke in South Africa: outcomes of home-based care in a low-resource rural setting. Top Stroke Rehabil. 2019 Aug 18;26(6):423–34.

3. Pillay-van Wyk V, Msemburi W, Laubscher R, Dorrington RE, Groenewald P, Glass T, et al. Mortality trends and differentials in South Africa from 1997 to 2012: second National Burden of Disease Study. Lancet Glob Heal. 2016

4. Maredza M, Bertram MY, Gómez-Olivé XF, Tollman SM. Burden of stroke attributable to selected lifestyle risk factors in rural South Africa. BMC Public Health [Internet]. 2016 Jan 1;16:143. Available from: http://search.ebscohost.com/login.aspx?direct=true&db=awn&AN=26869067&site=ehost-live&scope=site

5. Coovadia H, Jewkes R, Barron P, Sanders D, McIntyre D. The health and health system of South Africa: historical roots of current public health challenges. The Lancet. 2009.

6. Bertram MY, Katzenellenbogen J, Vos T, Bradshaw D, Hofman KJ. The disability adjusted life years due to stroke in South Africa in 2008 [Internet]. Vol. 8 Suppl A1, International journal of stroke: official journal of the International Stroke Society. 2013. p. 76–80. Available from: http://search.ebscohost.com/login.aspx?direct=true&db=awn&AN=23295022&site=ehost-live&scope=site

7. Maredza M, Bertram MY, Tollman SM. Disease burden of stroke in rural South Africa: An estimate of incidence, mortality and disability adjusted life years. BMC Neurol. 2015 Apr 12;15(1).

8. Eksteen G, Mungal-Singh V. Salt intake in South Africa: A current perspective. Journal of Endocrinology, Metabolism and Diabetes of South Africa. 2015.

9. Kabudula CW, Houle B, Collinson MA, Kahn K, Gómez-Olivé FX, Clark SJ, et al. Progression of the epidemiological transition in a rural South African setting: findings from population surveillance in Agincourt, 1993-2013. BMC Public Health. 2017 May 10;17(1).

10. WHO, The World Bank. Tracking Universal Health Coverage: 2017 Global Monitoring Report. World Health Organisation. 2017.

11. Mahlathi, P. and Dlamini J. Minimum data sets for human resources for health and the

For peer review only - http://bmjopen.bmj.com/site/about/guidelines.xhtml

surgical workforce in South Africa's health system: a rapid analysis of stock and migration. African Inst Heal Leadersh Dev. 2015

12. Naidoo S. The South African National Health Insurance: a revolution in health care delivery! J Public Health (Bangkok). 2012;34(1):149–150.

13. South African Contextualised stroke rehabilitation guideline (SACSRG), 2019. 2019;

14. Vallabhjee K D V. Western Cape Government. Healthcare 2030: the road to wellness. Cape Town: Western Cape Department of Health. 2014.

15. Kuper H, Hanefeld J. Debate: can we achieve universal health coverage without a focus on disability? BMC Health Serv Res. 2018

16. Hashemi G, Kuper H, Wickenden M. SDGs, Inclusive Health and the path to Universal Health Coverage. Disabil Glob South. 2017

17. Evans DB, Etienne C. Health systems financing and the path to universal coverage. Bulletin of the World Health Organization. 2010.

18. Louw Q, Twizeyemariya A, Grimmer K, Leibbrandt D. Estimating the costs and benefits of stroke rehabilitation in South Africa. J Eval Clin Pract. 2020 Aug 1;26(4):1181–7.

19. Levac D, Colquhoun H, O'Brien KK. Scoping studies: Advancing the methodology. Implement Sci. 2010 Sep 20;5(1).

20. van Niekerk S-M, Inglis-Jassiem G, Kamalakannan S, Fernandes S, Webster J, English R, et al. Achieving universal health coverage for people with stroke in South Africa: protocol for a scoping review. BMJ Open. 2020 Oct;10(10):e041221.

21. Tricco AC, Lillie E, Zarin W, O'Brien KK, Colquhoun H, Levac D, et al. PRISMA extension for scoping reviews (PRISMA-ScR): Checklist and explanation. Annals of Internal Medicine. 2018.

22. Olmen J Van, Criel B, Bhojani U, Marchal B, Belle S Van, Chenge MF, et al. The Health System Dynamics Framework: The introduction of an analytical model for health system analysis and its application to two case-studies. Heal Cult Soc. 2012;2(1):1–21.

23. World Health Organization (WHO). Framework on integrated, people-centred health services: Report by the Secretariat. World Heal Assem. 2016

24. Ritchie J, Lewis J, Nicholls C, Ormston R. Qualitative research practice: A guide for social science students and researchers [Internet]. 2013 [cited 2021 Jan 10]. Available from: https://books.google.co.za/books?hl=en&lr=&id=EQSIAwAAQBAJ&oi=fnd&pg=PP1&dq=Ritchie+J,+Lewis+J,+Nicholls+C,+Ormston+R&ots=l-QMhvTv3P&sig=Hz7vuHbw4WR5vGMtiWPszn_KSy4

For peer review only

25. Wasserman D. Community-based care of stroke patients in a rural African setting. South African Med J [Internet]. 2009 Jan 1;99(8):13. Available from: http://search.ebscohost.com/login.aspx?direct=true&db=awn&AN=803237&site=ehost-live&scope=site

26. Rouillard S, De Weerdt W, De Wit L, Jelsma J. Functioning at 6 months post stroke following discharge from inpatient rehabilitation. South African Med J. 2012;102(6):545–8.

27. Rhoda A, Cunningham N, Azaria S, Urimubenshi G. Provision of inpatient rehabilitation and challenges experienced with participation post discharge: quantitative and qualitative inquiry of African stroke patients. BMC Health Serv Res [Internet]. 2015 Jan 1;15:423. Available from: http://search.ebscohost.com/login.aspx?direct=true&db=awn&AN=26412081&site=ehost-live&scope=site

28. Groenewald R, Rhoda AJ. Multidisciplinary rehabilitation outcomes of stroke patients in the Western Cape of South Africa [Internet]. Vol. 23, African Journal for Physical, Health Education, Recreation and Dance. 2017. p. 267–76. Available from: http://search.ebscohost.com/login.aspx?direct=true&db=awn&AN=ajpherd-164230&site=ehost-live&scope=site

29. Joseph C. Determining the process of rehabilitation and the outcomes of patients at a specialised in-patient centre in the Western Cape. 2011;2–216. Available from: http://etd.uwc.ac.za/xmlui/handle/11394/2973

30. Ras T. An audit of geriatric stroke rehabilitation services at a post-acute hospital (Booth Memorial) in urban Cape Town, South Africa. Diss Univ Cape T. 2009;

31. Rhoda A, Mpofu R, DeWeerdt W. The rehabilitation of stroke patients at community health centres in the Western Cape. South African J Physiother. 2009;65(3).

32. De la Cornillère W. Participants' experience of the Bishop Lavis Rehabilitation Centre stroke group [Internet]. 2007. Available from: http://search.ebscohost.com/login.aspx?direct=true&db=awn&AN=NX0185175&site=ehost-live&scope=site

33. Leichtfuss U. Aspects of stroke rehabilitation in private acute-care hospitals. Master Philos (mphil) Fac Heal Sci. 2009

34. Cawood J. Rehabilitation outcomes of uninsured stroke survivors in the Helderberg Basin [Internet]. 2012. Available from: http://search.ebscohost.com/login.aspx?direct=true&db=awn&AN=NX0217654&site=ehost-live&scope=site

35. de Villiers L, Badri M, Ferreira M, Bryer A. Stroke outcomes in a socio-economically disadvantaged urban community. S Afr Med J. 2011 May;101(5):345–8.

For peer review only - http://bmjopen.bmj.com/site/about/guidelines.xhtml

36. Bham Z, Ross E. Traditional and western medicine: cultural beliefs and practices of South African Indian Muslims with regard to stroke [Internet]. Vol. 15, Ethnicity & Disease. 2005. p. 548–54. Available from: http://search.ebscohost.com/login.aspx?direct=true&db=awn&AN=16259475&site=ehost-live&scope=site

37. Cawood J, Visagie S. Stroke management and functional outcomes of stroke survivors in an urban Western Cape Province setting. South African J Occup Ther. 2016;46(3):21–6.

38. Mandizvidza V. Quality of current ischaemic stroke care practices in the Cape Metro Health District, South Africa. Diss Univ Cape T. 2017.

39. Kleineibst LJ. The effectiveness of a caregiver support programme to address the needs of primary caregivers of stroke patients in a low socio-economic community [Internet]. 2007. Available from: http://search.ebscohost.com/login.aspx?direct=true&db=awn&AN=NX0185273&site=ehost-live&scope=site

40. Cawood J, Visagie S. Environmental factors influencing participation of stroke survivors in a Western Cape setting. African J Disabil. 2015;4(1):1–9

41. Matshikiza. Barriers to acute stroke care at a tertiary Hospital in the Western Cape. 2019

42. Viljoen C. Audit of the quality and cost of acute inpatient stroke care in the general medical wards at Groote Schuur Hospital. 2014

43. Cawood J, Visagie S, Mji G. Impact of post-stroke impairments on activities and participation as experienced by stroke survivors in a Western Cape setting. South African J Occup Ther. 2016;46(2):10–5.

44. Hossain M. To investigate the Need for Palliative care in Cerebrovascular Accident (stroke) patients at Ladysmith Regional Hospital. 2014

45. Bryer A. The need for a community-based model for stroke care in South Africa. South African Med J [Internet]. 2009 Jan 1;99(8):10. Available from: http://search.ebscohost.com/login.aspx?direct=true&db=awn&AN=803234-1&site=ehost-live&scope=site

46. Hilton J. Factors that influence the quality of life of a caregiver caring for a patient with stroke. 2011

47. Cunningham NL. The profile and outcomes of stroke patients discharged from a hospital in the Eastern Cape [Internet]. 2012. Available from: http://search.ebscohost.com/login.aspx?direct=true&db=awn&AN=NX0215155&site=ehost-live&scope=site

48. de Villiers L, Kalula SZ, Burch VC. Does multidisciplinary stroke care improve

For peer review only

outcome in a secondary-level hospital in South Africa? [Internet]. Vol. 4, International journal of stroke: official journal of the International Stroke Society. 2009. p. 89–93. Available from: http://search.ebscohost.com/login.aspx?direct=true&db=awn&AN=19383048&site=ehost-live&scope=site

49. Rhoda A, Smith M, Putman K, Mpofu R, DeWeerdt W, DeWit L. Motor and functional recovery after stroke: a comparison between rehabilitation settings in a developed versus a developing country [Internet]. Vol. 14, BMC Health Services Research. 2014. p. 82. Available from: http://search.ebscohost.com/login.aspx?direct=true&db=awn&AN=24559193&site=ehost-live&scope=site

50. Mudzi W. Impact of caregiver education on stroke survivors and their caregivers. Dissertation. 2010

51. Parekh I, Rhoda A. Functional outcomes of stroke patients admitted to a tertiary hospital in the Western Cape, South Africa [Internet]. Vol. 69, South African Journal of Physiotherapy. 2013. p. 14. Available from: http://search.ebscohost.com/login.aspx?direct=true&db=awn&AN=722250&site=ehost-live&scope=site

52. Arowoiya A. Participation restrictions of stroke patients living in the community at selected community health centres in the metropole districts in the Western Cape, South Africa. Diss Univ West Cape. 2014

53. Mudzi W, Stewart A, Musenge E. Community participation of patients 12 months post-stroke in Johannesburg, South Africa. African J Prim Heal Care Fam Med. 2013;5(1):1–9.

54. Parekh IS. Factors influencing functional outcome of stroke patients admitted to a tertiary hospital [Internet]. 2012. Available from: http://search.ebscohost.com/login.aspx?direct=true&db=awn&AN=NX0214919&site=ehost-live&scope=site

55. Rhoda A, Mpofu R, De Weerdt W. Activity limitations of patients with stroke attending out-patient facilities in the Western Cape, South Africa [Internet]. Vol. 67, South African Journal of Physiotherapy. 2011. p. 16–22. Available from: http://search.ebscohost.com/login.aspx?direct=true&db=awn&AN=702551&site=ehost-live&scope=site

56. Blackwell Z, Littlejohns P. A review of the management of dysphagia: a South African perspective [Internet]. Vol. 42, Journal of Neuroscience Nursing. 2010. p. 61–70. Available from: http://search.ebscohost.com/login.aspx?direct=true&db=awn&AN=20422791&site=ehost-live&scope=site

For peer review only

57. Felemengas M. Caregiver experiences and perceptions of the effects of stroke in the family [Internet]. 2004. Available from: http://search.ebscohost.com/login.aspx?direct=true&db=awn&AN=NX0150149&site=ehost-live&scope=site

58. Mabunda SA. An Evaluation of the Role of an Intermediate Care Facility in the Continuum of Care in Western Cape, South Africa. Dissertation [Internet]. 2015;53(July):227. Available from: http://library.wur.nl/WebQuery/wurpubs/fulltext/353506

59. Groenewald RC. The Adaptation and Contextualization of the Bridges Stroke Self-Management Intervention for Patients Living With Stroke in the Western Cape, South Africa. 2018;(August). Available from: http://etd.uwc.ac.za/ii

60. Makganye TM. The experience of patients and caregivers following a stroke. 2015;(December).

61. Kotsokoane FM, Tshabalala MD, Nukeri AL, Mkhacwa WB. The level of integration of stroke survivors receiving rehabilitation services in Soshanguve clinics, South Africa [Internet]. Vol. 24, African Journal for Physical, Health Education, Recreation and Dance. 2018. p. 564–78. Available from: http://search.ebscohost.com/login.aspx?direct=true&db=awn&AN=ajpherd-181896&site=ehost-live&scope=site

62. Kusambiza-kiingi A. Community reintegration and satisfaction of survivors of stroke receiving physiotherapy services in the community health centres within the Johannesburg area. 2016;1–2.

63. Ntamo PN. Poor attendance of physiotherapy treatment by stroke out-patients in Mthatha General Hospital [Internet]. 2011. Available from: http://search.ebscohost.com/login.aspx?direct=true&db=awn&AN=NX0207219&site=ehost-live&scope=site

64. Taylor A, Ntusi NAB. Guest Editorial: Evolving concepts of stroke and stroke management in South Africa: Quo vadis? [Internet]. Vol. 109, South African Medical Journal. 2019. p. 69–71. Available from: http://search.ebscohost.com/login.aspx?direct=true&db=awn&AN=samj-184307&site=ehost-live&scope=site

65. Biggs D. Health Promotion Needs of Stroke Patients Accessing Community Health Centres in the Metropole Region of the Western Cape Health Promotion Needs of Stroke Patients Accessing Community Health Centres in the. 2005;(November).

66. Thomas M, Greenop K. Caregiver Experiences and Perceptions of Stroke. Heal SA Gesondheid [Internet]. 2008 Jan 1;13(1):29–40. Available from: http://search.ebscohost.com/login.aspx?direct=true&db=awn&AN=IM-009014&site=ehost-live&scope=site

For peer review only

67. Posner G. the Experiences of Employed Caregivers, Working in Private South African Homes, With Patients Who Have Suffered From a Stroke. 2015; Available from: http://wiredspace.wits.ac.za/jspui/bitstream/10539/19598/2/MASTERS DISSERTATION G POSNER.pdf

68. Smith JL. Self-management strategies employed by stroke survivors in the Western Cape, South Africa. Diss Univ Cape T. 2019

69. Mashau NS, Netshandama VO, Mudau MJ. Self-reported impact of caregiving on voluntary home-based caregivers in Mutale Municipality, South Africa. African J Prim Heal Care Fam Med. 2016;8(2):1–5.

70. Maleka M, Stewart AS, Hale L. The experience of living with stroke in low urban and rural socioeconomic areas of South Africa [Internet]. South African Journal of Physiotherapy. 2012. p. 5–29. Available from: http://search.ebscohost.com/login.aspx?direct=true&db=awn&AN=705374&site=ehost-live&scope=site

71. Rhoda AJ. Health-related quality of life of patients six months poststroke living in the Western Cape, South Africa. African J Disabil. 2014;3(1):1–6.

72. Kusambiza-Kiingi A, Maleka D, Ntsiea V. Stroke survivors' levels of community reintegration, quality of life, satisfaction with the physiotherapy services and the level of caregiver strain at community health centres within the Johannesburg area. African J Disabil. 2017;6:1–8.

73. Hassan S, Visagie S, Mji G. Strain experienced by caregivers of stroke survivors in the Western Cape [Internet]. Vol. 67, South African Journal of Physiotherapy. 2011. p. 4–8. Available from: http://search.ebscohost.com/login.aspx?direct=true&db=awn&AN=702553&site=ehost-live&scope=site

74. Masuku KP, Mophosho M, Tshabalala M. "I felt pain. Deep pain...": Experiences of primary caregivers of stroke survivors with aphasia in a South African township. African J Disabil. 2018;7:1–7.

75. Biggs D, Rhoda A. Health risk behaviours of stroke patients in the Western Cape, South Africa. South African J Physiother [Internet]. 2008 Jan 1;64(1):38–42. Available from: http://search.ebscohost.com/login.aspx?direct=true&db=awn&AN=577099&site=ehost-live&scope=site

76. Burton A. South Africa: stroke units out of the blue [Internet]. Vol. 15, The Lancet. Neurology. 2016. p. 359–60. Available from: http://search.ebscohost.com/login.aspx?direct=true&db=awn&AN=26971657&site=ehost-live&scope=site

77. Elloker T. Social support and participation restrictions in patients living with stroke in

For peer review only - http://bmjopen.bmj.com/site/about/guidelines.xhtml

the Western Cape, SOuth Africa. Dissertation. 2015

78. Cunningham N, Rhoda A. Outcomes of stroke patients discharged from an in-patient facility in the Eastern Cape, South Africa: a mixed methods design [Internet]. Vol. 70, South African Journal of Physiotherapy. 2014. p. 26–31. Available from: http://search.ebscohost.com/login.aspx?direct=true&db=awn&AN=722129&site=ehost-live&scope=site

79. Mamabolo V. The Influence of Demographic, Environmental and Physical Factors on Functional Independence Post Stroke Correspondence to : 2008;(011):19–22.

80. Botha JH. The refinement of a booklet on stroke care at home. 2008

81. Faux S. Managing stroke survivors int he community. Mod Med South Africa [Internet]. 2006 Jan 1;31(1 January):8–14. Available from: http://search.ebscohost.com/login.aspx?direct=true&db=awn&AN=D-18283&site=ehost-live&scope=site

82. Bryer A, Connor M, Haug P, Cheyip B, Staub H, Tipping B, et al. South African guideline for management of ischaemic stroke and transient ischaemic attack 2010: a guideline from the South African Stroke Society (SASS) and the SASS Writing Committee [Internet]. Vol. 100, S A M J South African Medical Journal. 2010. p. 747–78. Available from: http://search.ebscohost.com/login.aspx?direct=true&db=awn&AN=21081029&site=ehost-live&scope=site

83. Hussey M, Maclachlan M, Mji G. Barriers to the Implementation of the Health and Rehabilitation Articles of the United Nations Convention on the Rights of Persons with Disabilities in South Africa. Kerman Univ Med Sci [Internet]. 2017;6(4):207–218. Available from: http://dx.doi.org/10.15171/ijhpm.2016.117

84. National Department of Health. Strengthening the South African health system: Towards an integrated and unified health system. Pres Heal Summit 2018.

85. Vindigni SM, Riley PL, Kimani F, Willy R, Warutere P, Sabatier JF, et al. Kenya's emergency-hire nursing programme: A pilot evaluation of health service delivery in two districts. Hum Resour Health. 2014

86. Lehmann U, Dieleman M, Martineau T. Staffing remote rural areas in middle- and low-income countries: A literature review of attraction and retention. BMC Health Serv Res. 2008

87. Hatcher AM, Onah M, Kornik S, Peacocke J, Reid S. Placement, support, and retention of health professionals: National, cross-sectional findings from medical and dental community service officers in South Africa. Hum Resour Health. 2014

88. Chimatiro GL, Rhoda AJ. Scoping review of acute stroke care management and rehabilitation in low and middle-income countries. BMC Health Serv Res. 2019

For peer review only - http://bmjopen.bmj.com/site/about/guidelines.xhtml

Nov;19(1):789.

89. Connor M. Stroke management in South Africa - who is responsible?: guest editorial [Internet]. Vol. 8, South African Psychiatry Review. 2005. p. 125–6. Available from: http://search.ebscohost.com/login.aspx?direct=true&db=awn&AN=381538&site=ehost-live&scope=site

90. Bates B, Choi JY, Duncan PW, Glasberg JJ, Graham GD, Katz RC, et al. Veterans Affairs/Department of Defense Clinical Practice Guideline for the Management of Adult Stroke Rehabilitation Care: Executive summary. Stroke. 2005.

91. Maclean N, Pound P, Wolfe C, Rudd A. Qualitative analysis of stroke patients' motivation for rehabilitation. Br Med J. 2000

LEGEND

Figure 1: Analytic framework for health system-related factors that limit or support UHC, incorporating components from the Health Systems Dynamics Framework Components of the analytical framework that incorporates components from the Health Systems Dynamics Framework (22) and WHO Framework on Integrated People-Centred health Services and WHO Framework on integrated people-centred health services (23)

Figure 2: PRISMA flow chart

Figure 3: Limiting and supporting factors toward achieving UHC

Supplementary File

Supplementary File S1: Search Strategy

Supplementary File S2: Framework component definitions

Supplementary file S3: Basic demographic information of included records (N=59)

Supplementary file S4: Components assessed in included studies

For peer review only

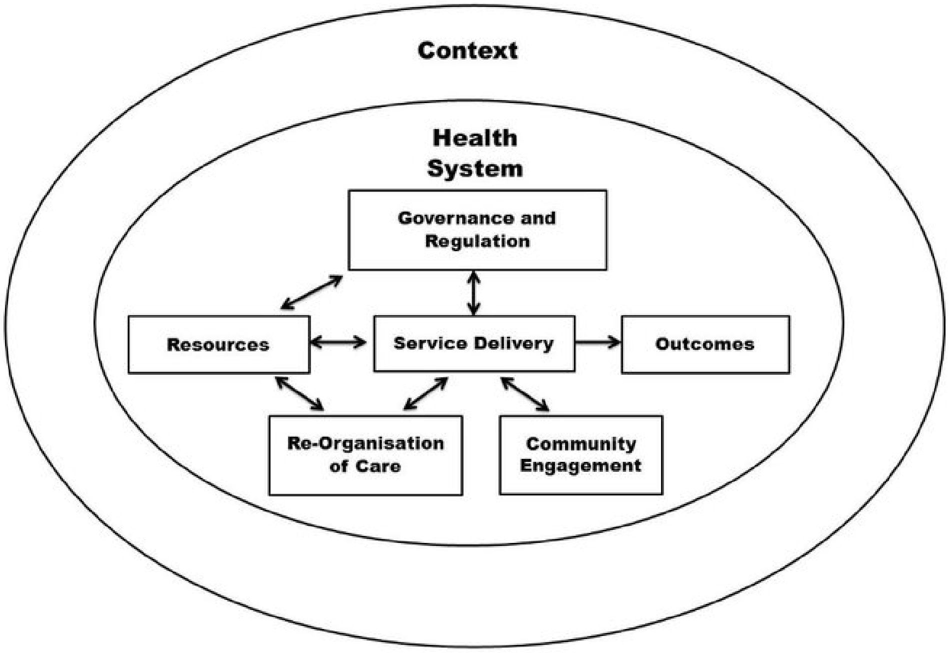

Figure 1: Analytic framework for health system-related factors that limit or support UHC, incorporating components from the Health Systems Dynamics Framework Components of the analytical framework that incorporates components from the Health Systems Dynamics Framework (22) and WHO Framework on Integrated People-Centred health Services and WHO Framework on integrated people-centred health services (23)

182x136mm (96 x 96 DPI)

Figure-2: PRISMA flowchart of the scoping review.

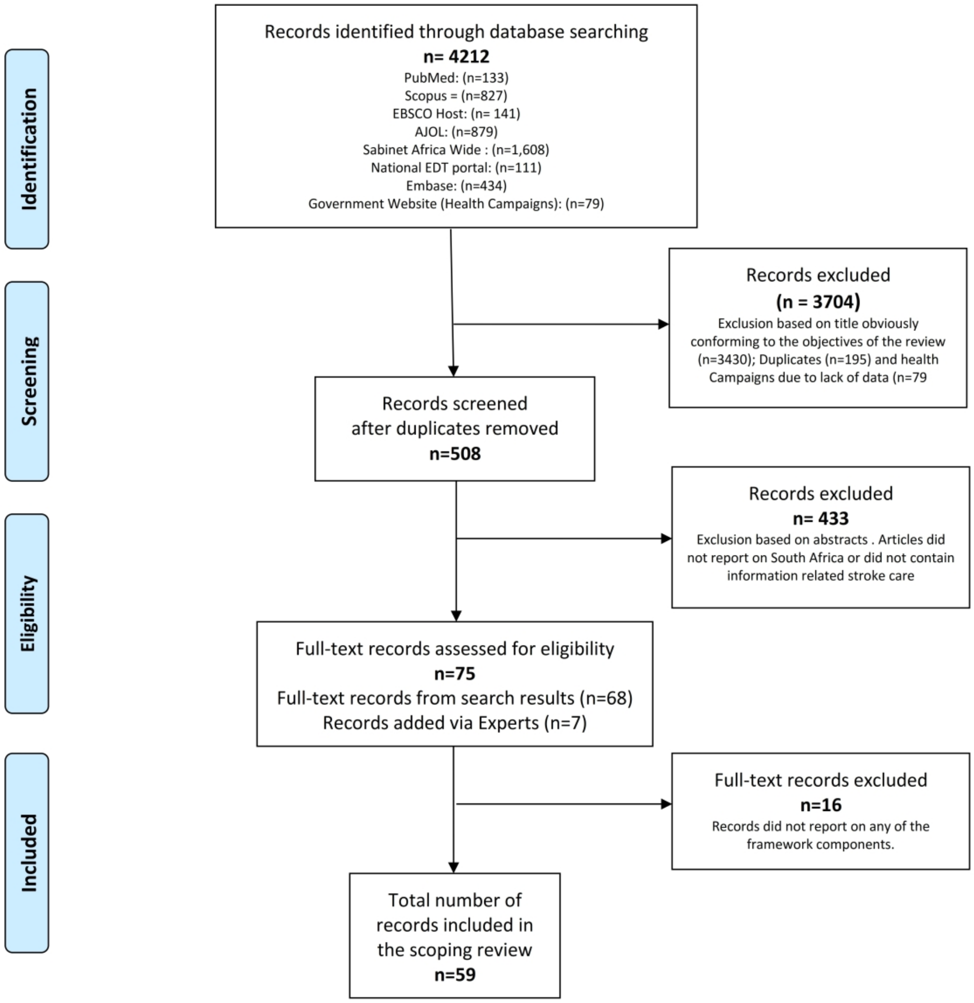

Figure 2: PRISMA flow chart

191x201mm (600 x 600 DPI)

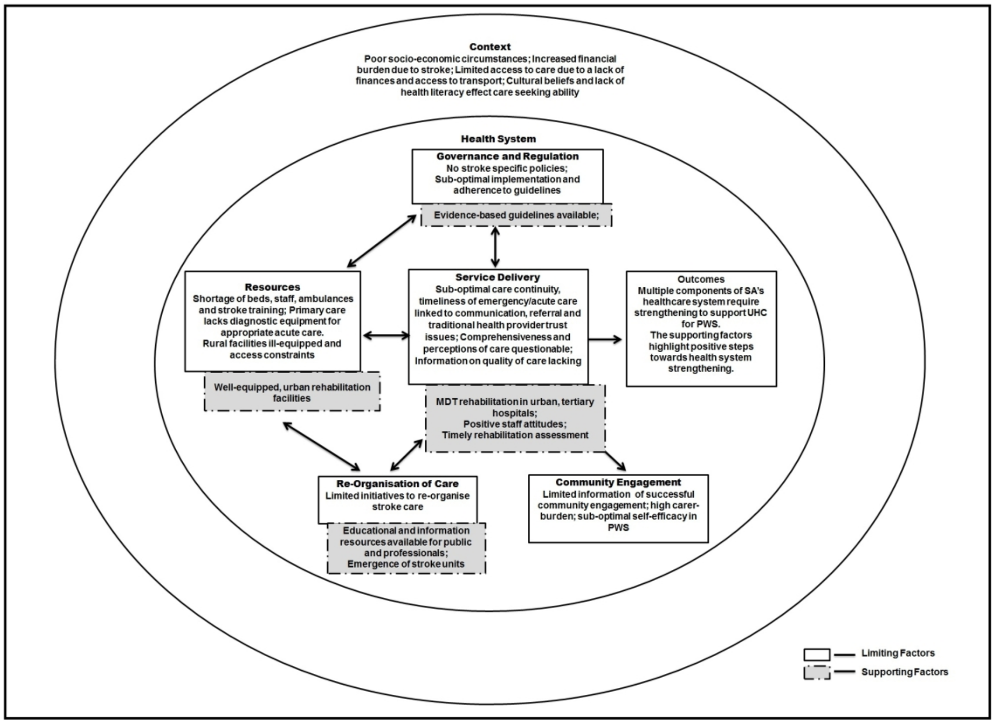

Figure 3: Limiting and supporting factors toward achieving UHC

531x388mm (96 x 96 DPI)

Supplemantary File - 1

Achieving Universal Health Coverage for people with stroke in South Africa:

OVID Search

Database: Embase <1980 to 2020 Week 24>, Global Health <1910 to 2020 Week 23>, Journals@Ovid Full Text <June 17, 2020>, APA PsycExtra <1908 to June 08, 2020>, APA PsycInfo <1806 to June Week 2 2020>, LSHTM Journals@Ovid, Econlit <1886 to June 11, 2020>, Ovid MEDLINE(R) and Epub Ahead of Print, In-Process & Other Non-Indexed Citations, Daily and Versions(R) <1946 to June 16, 2020>, Social Policy and Practice <202004>
Search Strategy:
----

1 Stroke.mp. [mp=ti, ab, hw, tn, ot, dm, mf, dv, kw, fx, dq, bt, id, cc, tx, sh, ct, tc, tm, mh, nm, kf, ox, px, rx, an, ui, sy, pt] (1263546)
2 cerebro vascular accident.mp. [mp=ti, ab, hw, tn, ot, dm, mf, dv, kw, fx, dq, bt, id, cc, tx, sh, ct, tc, tm, mh, nm, kf, ox, px, rx, an, ui, sy, pt] (560)
3 ischaemia.mp. [mp=ti, ab, hw, tn, ot, dm, mf, dv, kw, fx, dq, bt, id, cc, tx, sh, ct, tc, tm, mh, nm, kf, ox, px, rx, an, ui, sy, pt] (140097)
4 hemorrhage.mp. [mp=ti, ab, hw, tn, ot, dm, mf, dv, kw, fx, dq, bt, id, cc, tx, sh, ct, tc, tm, mh, nm, kf, ox, px, rx, an, ui, sy, pt] (923222)
5 Universal health coverage.mp. [mp=ti, ab, hw, tn, ot, dm, mf, dv, kw, fx, dq, bt, id, cc, tx, sh, ct, tc, tm, mh, nm, kf, ox, px, rx, an, ui, sy, pt] (9467)
6 universal access.mp. [mp=ti, ab, hw, tn, ot, dm, mf, dv, kw, fx, dq, bt, id, cc, tx, sh, ct, tc, tm, mh, nm, kf, ox, px, rx, an, ui, sy, pt] (12850)
7 Universal health care.mp. [mp=ti, ab, hw, tn, ot, dm, mf, dv, kw, fx, dq, bt, id, cc, tx, sh, ct, tc, tm, mh, nm, kf, ox, px, rx, an, ui, sy, pt] (7370)
8 universal health access.mp. [mp=ti, ab, hw, tn, ot, dm, mf, dv, kw, fx, dq, bt, id, cc, tx, sh, ct, tc, tm, mh, nm, kf, ox, px, rx, an, ui, sy, pt] (64)
9 stroke disability.mp. [mp=ti, ab, hw, tn, ot, dm, mf, dv, kw, fx, dq, bt, id, cc, tx, sh, ct, tc, tm, mh, nm, kf, ox, px, rx, an, ui, sy, pt] (1581)
10 stroke rehabilitation.mp. [mp=ti, ab, hw, tn, ot, dm, mf, dv, kw, fx, dq, bt, id, cc, tx, sh, ct, tc, tm, mh, nm, kf, ox, px, rx, an, ui, sy, pt] (30385)
11 stroke care.mp. [mp=ti, ab, hw, tn, ot, dm, mf, dv, kw, fx, dq, bt, id, cc, tx, sh, ct, tc, tm, mh, nm, kf, ox, px, rx, an, ui, sy, pt] (18395)
12 treatment access.mp. [mp=ti, ab, hw, tn, ot, dm, mf, dv, kw, fx, dq, bt, id, cc, tx, sh, ct, tc, tm, mh, nm, kf, ox, px, rx, an, ui, sy, pt] (6626)
13 health systems.mp. [mp=ti, ab, hw, tn, ot, dm, mf, dv, kw, fx, dq, bt, id, cc, tx, sh, ct, tc, tm, mh, nm, kf, ox, px, rx, an, ui, sy, pt] (111350)
14 South Africa.mp. [mp=ti, ab, hw, tn, ot, dm, mf, dv, kw, fx, dq, bt, id, cc, tx, sh, ct, tc, tm, mh, nm, kf, ox, px, rx, an, ui, sy, pt] (235927)
15 Eastern Cape.mp. [mp=ti, ab, hw, tn, ot, dm, mf, dv, kw, fx, dq, bt, id, cc, tx, sh, ct, tc, tm, mh, nm, kf, ox, px, rx, an, ui, sy, pt] (4355)
16 Free State.mp. [mp=ti, ab, hw, tn, ot, dm, mf, dv, kw, fx, dq, bt, id, cc, tx, sh, ct, tc, tm, mh, nm, kf, ox, px, rx, an, ui, sy, pt] (14110)
17 Gauteng.mp. [mp=ti, ab, hw, tn, ot, dm, mf, dv, kw, fx, dq, bt, id, cc, tx, sh, ct, tc, tm, mh, nm, kf, ox, px, rx, an, ui, sy, pt] (4356)
18 KwaZulu-Natal.mp. [mp=ti, ab, hw, tn, ot, dm, mf, dv, kw, fx, dq, bt, id, cc, tx, sh, ct, tc, tm, mh, nm, kf, ox, px, rx, an, ui, sy, pt] (11901)

For peer review only

19 Limpopo.mp. [mp=ti, ab, hw, tn, ot, dm, mf, dv, kw, fx, dq, bt, id, cc, tx, sh, ct, tc, tm, mh, nm, kf, ox, px, rx, an, ui, sy, pt] (3368)
20 Mpumalanga.mp. [mp=ti, ab, hw, tn, ot, dm, mf, dv, kw, fx, dq, bt, id, cc, tx, sh, ct, tc, tm, mh, nm, kf, ox, px, rx, an, ui, sy, pt] (1992)
21 Northern Cape.mp. [mp=ti, ab, hw, tn, ot, dm, mf, dv, kw, fx, dq, bt, id, cc, tx, sh, ct, tc, tm, mh, nm, kf, ox, px, rx, an, ui, sy, pt] (997)
22 North West.mp. [mp=ti, ab, hw, tn, ot, dm, mf, dv, kw, fx, dq, bt, id, cc, tx, sh, ct, tc, tm, mh, nm, kf, ox, px, rx, an, ui, sy, pt] (39969)
23 Western Cape.mp. [mp=ti, ab, hw, tn, ot, dm, mf, dv, kw, fx, dq, bt, id, cc, tx, sh, ct, tc, tm, mh, nm, kf, ox, px, rx, an, ui, sy, pt] (8316)
24 1 or 2 or 3 or 4 (2162628)
25 5 or 6 or 7 or 8 (28371)
26 9 or 10 or 11 or 12 (54898)
27 15 or 16 or 17 or 18 or 19 or 20 or 21 or 22 or 23 (83478)
28 14 or 27 (286527)
29 13 or 25 (135636)
30 24 and 29 (7926)
31 24 and 26 and 29 (655)
32 24 and 28 and 29 (433)

****

For peer review only

UHC for Stroke care in SA - Scopus Search results

(Stroke OR Universal health coverage) AND (Health system) AND (LIMIT-TO (AFFILCOUNTRY,"South Africa")) AND (LIMIT-TO (PUBYEAR,2020) OR LIMIT-TO (PUBYEAR,2019) OR LIMIT-TO (PUBYEAR,2018) OR LIMIT-TO (PUBYEAR,2017) OR LIMIT-TO (PUBYEAR,2016) OR LIMIT-TO (PUBYEAR,2015) OR LIMIT-TO (PUBYEAR,2014) OR LIMIT-TO (PUBYEAR,2013) OR LIMIT-TO (PUBYEAR,2012) OR LIMIT-TO (PUBYEAR,2011) OR LIMIT-TO (PUBYEAR,2010) OR LIMIT-TO (PUBYEAR,2009) OR LIMIT-TO (PUBYEAR,2008) OR LIMIT-TO (PUBYEAR,2007) OR LIMIT-TO (PUBYEAR,2006) OR LIMIT-TO (PUBYEAR,2004) OR LIMIT-TO (PUBYEAR,2003) OR LIMIT-TO (PUBYEAR,2002) OR LIMIT-TO (PUBYEAR,2000)) AND (LIMIT-TO (openaccess,1)) AND (LIMIT-TO (SUBJAREA,"MEDI") OR LIMIT-TO (SUBJAREA,"SOCI") OR LIMIT-TO (SUBJAREA,"MULT") OR LIMIT-TO (SUBJAREA,"NURS") OR LIMIT-TO (SUBJAREA,"HEAL") OR LIMIT-TO (SUBJAREA,"ENVI") OR LIMIT-TO (SUBJAREA,"PSYC") OR LIMIT-TO (SUBJAREA,"ECON") OR LIMIT-TO (SUBJAREA,"ARTS") OR LIMIT-TO (SUBJAREA,"NEUR")) AND (LIMIT-TO (PUBSTAGE,"final") OR LIMIT-TO (PUBSTAGE,"aip"))

For peer review only

Print Search History: EBSCOhost

		Narrow by SubjectThesaurus: - qualitative research Narrow by SubjectThesaurus: - primary care Narrow by SubjectThesaurus: - evaluation research Narrow by SubjectThesaurus: - health behavior Narrow by SubjectThesaurus: - community health services Narrow by SubjectThesaurus: - health outcome assessment Narrow by SubjectThesaurus: - government policy Narrow by SubjectThesaurus: - world health Narrow by SubjectThesaurus: - quality of life Narrow by SubjectThesaurus: - health services accessibility Narrow by SubjectThesaurus: - health promotion Narrow by SubjectThesaurus: - public health Search modes - Boolean/Phrase		
S5	(stroke or cerebrovascular accident or cva or cerebral vascular event or cve or transient ischaemic attack or tia) OR (stroke rehabilitation or stroke recovery) OR stroke	Limiters - Published Date: 20050101-20200631 Expanders - Apply related words; Apply equivalent subjects Narrow by Language: - english	Interface - EBSCOhost Research Databases Search Screen - Advanced Search Database - Academic Search Complete	16,739

prevention OR stroke patients AND universal health care OR universal health coverage OR universal health coverage in south africa AND health system OR health systems strengthening OR (health system or health services) OR health systems management AND south africa	Narrow by SubjectThesaurus: - public health surveillance Narrow by SubjectThesaurus: - primary health care Narrow by SubjectThesaurus: - national health services Narrow by SubjectThesaurus: - health insurance Narrow by SubjectThesaurus: - health disparities Narrow by SubjectThesaurus: - health care reform Narrow by SubjectThesaurus: - health programs Narrow by SubjectThesaurus: - chronic diseases Narrow by SubjectThesaurus: - economics Narrow by SubjectThesaurus: - systematic reviews (medical research) Narrow by SubjectThesaurus: - qualitative research Narrow by SubjectThesaurus: - primary care Narrow by SubjectThesaurus: - evaluation research Narrow by SubjectThesaurus: - health behavior Narrow by SubjectThesaurus: - community health services Narrow by SubjectThesaurus: -

		health outcome assessment<br>Narrow by SubjectThesaurus: - government policy<br>Narrow by SubjectThesaurus: - world health<br>Narrow by SubjectThesaurus: - quality of life<br>Narrow by SubjectThesaurus: - health services accessibility<br>Narrow by SubjectThesaurus: - health promotion<br>Narrow by SubjectThesaurus: - public health<br>Search modes - Boolean/Phrase		
S4	(stroke or cerebrovascular accident or cva or cerebral vascular event or cve or transient ischaemic attack or tia) OR (stroke rehabilitation or stroke recovery) OR stroke prevention OR stroke patients AND universal health care OR universal health coverage OR universal health coverage in south africa AND health system OR health systems strengthening OR (health system or health services) OR health systems management AND south africa	Limiters - Published Date: 20050101-20200631<br>Expanders - Apply related words; Apply equivalent subjects<br>Narrow by SubjectThesaurus: - public health surveillance<br>Narrow by SubjectThesaurus: - primary health care<br>Narrow by SubjectThesaurus: - national health services<br>Narrow by SubjectThesaurus: - health insurance<br>Narrow by SubjectThesaurus: - health disparities<br>Narrow by SubjectThesaurus: - health care reform<br>Narrow by SubjectThesaurus: -	Interface - EBSCOhost Research Databases<br>Search Screen - Advanced Search<br>Database - Academic Search Complete	17,251

Print Search History: EBSCOhost

health programs
Narrow by SubjectThesaurus: - chronic diseases
Narrow by SubjectThesaurus: - economics
Narrow by SubjectThesaurus: - systematic reviews (medical research)
Narrow by SubjectThesaurus: - qualitative research
Narrow by SubjectThesaurus: - primary care
Narrow by SubjectThesaurus: - evaluation research
Narrow by SubjectThesaurus: - health behavior
Narrow by SubjectThesaurus: - community health services
Narrow by SubjectThesaurus: - health outcome assessment
Narrow by SubjectThesaurus: - government policy
Narrow by SubjectThesaurus: - world health
Narrow by SubjectThesaurus: - quality of life
Narrow by SubjectThesaurus: - health services accessibility
Narrow by SubjectThesaurus: - health promotion
Narrow by SubjectThesaurus: -

		public health Search modes - Boolean/Phrase		
S3	(stroke or cerebrovascular accident or cva or cerebral vascular event or cve or transient ischaemic attack or tia) OR (stroke rehabilitation or stroke recovery) OR stroke prevention OR stroke patients AND universal health care OR universal health coverage OR universal health coverage in south africa AND health system OR health systems strengthening OR (health system or health services) OR health systems management AND south africa	Limiters - Published Date: 20050101-20200631 Expanders - Apply related words; Apply equivalent subjects Narrow by SubjectThesaurus: - public health Search modes - Boolean/Phrase	Interface - EBSCOhost Research Databases Search Screen - Advanced Search Database - Academic Search Complete	84,912
S2	(stroke or cerebrovascular accident or cva or cerebral vascular event or cve or transient ischaemic attack or tia) OR (stroke rehabilitation or stroke recovery) OR stroke prevention OR stroke patients AND universal health care OR universal health coverage OR universal health coverage in south africa AND health system OR health systems strengthening OR (health system or health services) OR health systems management AND south africa	Limiters - Published Date: 20050101-20200631 Expanders - Apply related words; Apply equivalent subjects Search modes - Boolean/Phrase	Interface - EBSCOhost Research Databases Search Screen - Advanced Search Database - Academic Search Complete	878,271
S1	(stroke or cerebrovascular accident or cva or cerebral	Limiters - Published Date: 20050101-20200631	Interface - EBSCOhost Research Databases Search Screen - Advanced	878,271

vascular event or cve or transient ischaemic attack or tia) OR (stroke rehabilitation or stroke recovery) OR stroke prevention OR stroke patients AND universal health care OR universal health coverage OR universal health coverage in south africa AND health system OR health systems strengthening OR (health system or health services) OR health systems management OR health system financing	Expanders - Apply related words; Apply equivalent subjects Search modes - Boolean/Phrase	Search Database - Academic Search Complete

Supplementary file S2

FRAMEWORK COMPONENT	DESCRIPTION (data items)
**Governance/Regulation**	**Description:** a wide range of steering and rulemaking-related functions carried out by governments/decisions makers as they seek to achieve national and/or provincial health policy objectives that are conducive to UHC<br>**Data Items:** Healthcare policies at national or provincial levels; resource allocation policies; accountability monitoring; coordination and regulations; clinical treatment guidelines.
**Resources**	**Description:** All resources specific to the health care facility – including the physical structure and resources enabling or hindering delivery of health services.<br>**Data Items:**<br>**Infrastructure:** Accessibility of the health care facilities; maintenance of infrastructure; availability of equipment/testing facilities (e.g. CT Scans)<br>**Human resources:** Availability; health workforce distribution – health professions/experience or specialisation/gender; role definitions; undergraduate & continuous training; workload; patient vs therapist ratio.<br>**Financial resources:** Finance allocation and affordability; funding sources; healthcare packages; salaries/fair wages.; sustainability.
**Service Delivery**	**Description:** Delivery of different health services as well as user experience.<br>**Data Items:** Level of care; comprehensiveness; quality and/or perceptions of care; multi-professional health teams; continuity of care, timeliness of care; health services and service providers (private/public; for-profit or not–for-profit, formal or informal, professional or non-professional, allopathic or traditional, remunerated or voluntary).
**Context**	**Description:** All contextual factors influencing the patient/community access of the health care system.<br>**Data Items:** Social determinants of health: socio-economic, education, health literacy, technological, cultural, political and environmental environments.
**Re-orientation of care**	**Description:** New and innovate health care solutions to improve coordination of health services and continuous health care; and intersectoral coordination.<br>**Data Items:** New technologies and strategies (eHealth; shared electronic medical records; telemedicine; m-health)
**Community engagement**	**Description:** Engaging and empowering individuals, families, communities and informal caregivers to facilitate common decision-making and self-efficacy. Reaching underserved and marginalised communities.

Application of the key characteristics of the analytical framework

For peer review only

FRAMEWORK COMPONENT	DESCRIPTION (data items)
**Governance/Regulation**	**Description:** a wide range of steering and rulemaking-related functions carried out by governments/decisions makers as they seek to achieve national and/or provincial health policy objectives that are conducive to UHC<br>**Data Items:** Healthcare policies at national or provincial levels; resource allocation policies; accountability monitoring; coordination and regulations; clinical treatment guidelines.
**Resources**	**Description:** All resources specific to the health care facility – including the physical structure and resources enabling or hindering delivery of health services.<br>**Data Items:**<br>**Infrastructure:** Accessibility of the health care facilities; maintenance of infrastructure; availability of equipment/testing facilities (e.g. CT Scans)<br>**Human resources:** Availability; health workforce distribution – health professions/experience or specialisation/gender; role definitions; undergraduate & continuous training; workload; patient vs therapist ratio.<br>**Financial resources:** Finance allocation and affordability; funding sources; healthcare packages; salaries/fair wages.; sustainability.
**Service Delivery**	**Description:** Delivery of different health services as well as user experience.<br>**Data Items:** Level of care; comprehensiveness; quality and/or perceptions of care; multiprofessional health teams; referral systems; service delivery models; health services and service providers (private/public; for-profit or not–for-profit, formal or informal, professional or non-professional, allopathic or traditional, remunerated or voluntary).
**Context**	**Description:** All contextual factors influencing the patient/community access of the health care system.<br>**Data Items:** Social determinants of health: socio-economic, education, health literacy, technological, cultural, political and environmental environments.
**Re-orientation of care**	**Description:** New and innovate health care solutions to improve coordination of health services and continuous health care; and intersectoral coordination.<br>**Data Items:** New technologies and strategies (eHealth; shared electronic medical records; telemedicine; m-health)
**Community engagement**	**Description:** Engaging and empowering individuals, families, communities and informal caregivers to facilitate common decision-making and self-efficacy. Reaching underserved and marginalised communities.

For peer review only

Supplementary file S3. Detailed demographic information of all included records (N=59)

S.no	Author (year)	Province	Area	Level of care	Literature	Aim	Study design	Sample characteristics
1	Arowoiya (2014)	Western Cape	Urban	Primary Healthcare (CHC)	Dissertations	To determine and explore the participation restrictions experienced by stroke patients	Mixed Methods (Survey + FGDs)	120 stroke patients receiving Physiotherapy for survey & 2 FGDs with 17 stroke patients
2	Bham & Ross 2005	Not reported	Undefined	Community	Primary Literature (peer reviewed publications)	To investigate the beliefs of caregivers and traditional healers within the South African Indian Muslim community regarding the etiology and treatment of stroke and the persons likely to be consulted in this regard	Descriptive case study design (Qualitative)	10 SAIM caregivers of people who had sustained strokes, as well as 10 SAIM traditional healers, who had treated stroke patients.
3	Biggs (2005)	Western Cape	Urban	Primary Healthcare (CHC)	Dissertations	To determine the health promotion needs of stroke patients accessing selected Community Health Centres in the Metropole region of the Western Cape.	Mixed Methods (Survey + In-depth interview)	418 stroke patients, representing each of the health districts of the Metropole region of the Western Cape for the survey and 12 stroke patients for Interviews
4	Biggs & Rhoda 2008	Western Cape	Urban	Primary Healthcare (CHC)	Primary Literature (peer reviewed publications)	To determine the health risk behaviours and factors that influence these behaviours of stroke patients in the Metropole Region of the Western Cape, South Africa	Mixed Methods (Survey + In-depth interview)	417 stroke patients – survey 12 stroke patients for Interviews
5	Blackwell & Littlejohns 2010	Gauteng,	Undefined	Undefined	Primary Literature (peer reviewed publications)	To measure the prevalence and review the assessment and management strategies related to dysphagia in three private rehabilitation clinics in South Africa	Review of records and thematic analysis	30 stroke patient records from three private rehabilitation clinics – total 90 records
6	Botha (2008)	Western Cape	Undefined	Community	Dissertations	To refine and pilot a training booklet for caregivers of stroke survivors for further	Mixed methods (Literature review +	Sample – 1: 11 Stroke patients in WCRC Sample – 2: 1Family members /other

For peer review only

						implementation	Checklist development + survey + FGDs)	carers of Stroke patients in WCRC<br>Sample – 3: 4 Home based carers and 22 nursing assistants attending carer training at WCRC<br>Sample – 4: 4 Stroke patients and their 4 caregivers participating in home-based care programme at university of Western Cape in Nyanga<br>Sample -5: Stroke patients in WCRC<br>Total 15 stroke patients and 31 caregivers
7	Bryer (2009)	South Africa	Undefined	Undefined	Primary Literature (peer reviewed publications)	There is an urgent need to develop a model of community-based stroke care with appropriate<br>Rehabilitation facilities and trained professionals<br>In South Africa, particularly in under-resourced<br>areas	Editorial	NA
8	Bryer et al (2010)	South Africa	NA	NA	Primary Literature (peer reviewed publications, Clinical Guideline)	The objective was to update the guideline published in 2000, to place the recommendations within the current South African context, and to grade evidence according to the level of scientific rigour for management of ischaemic stroke and transient ischaemic attack 2010	SASS writing committee Guidelines	NA
9	Burton 2016	South Africa	Undefined	Undefined	Primary Literature (peer reviewed publications)	To investigate the efforts of a woman with a talent for getting things done for bringing stroke units out of the blue for South Africa	Editorial	NA
10	Cawood 2012	Western Cape	Urban	Community	Dissertations	To determine if uninsured stroke survivors living in the Helderberg Basin (Western Cape) reached their optimal	A descriptive, mixed methods study (Surev + Interview)	53 stroke survivors (quantitative)<br>5 Stroke survivors (qualitative)

For peer review only

						rehabilitation outcome levels and if not, what environmental barriers contributed to this.		
11	Cawood& Visagie (2015)	Western Cape	Urban	Community	Primary Literature (peer reviewed publications)	To determine environmental barriers and facilitators to participation experienced by a group of stroke survivors in the Western Cape province of South Africa.	A descriptive, mixed methods study (Surev + Interview)	53 stroke survivors (quantitative)<br>5 Stroke survivors (qualitative)
12	Cawood & Visagie (2016)	Western Cape	Urban	Community	Primary Literature (peer reviewed publications)	To describe the functional outcomes achieved by stroke survivors in an urban Western Cape Province setting to add to the information on stroke management	A descriptive, mixed methods study (Surev + Interview)	53 stroke survivors (quantitative)<br>5 Stroke survivors (qualitative)
13	Cawood et al (2016)	Western Cape	Urban	Community	Primary Literature (peer reviewed publications)	To explore causal connections between impairments, activity limitations and participation restrictions after stroke.	Cross-sectional Study	53 stroke survivors
14	Connor (2005)	Sub Saharan Africa	Undefined	Undefined	Primary Literature (peer reviewed publications)	To understand the burden of stroke in black populations in sub-Saharan Africa	Systematic Review	All articles relevant to stroke in Sub Saharan Africa
15	Cunningham (2012)	Eastern cape	Urban	Uitenhage Provincial Hospital	Dissertations	To determine and explore the outcomes of stroke patients admitted to Uitenhage provincial hospital	Mixed Methods (quantitative survey + secondary data analysis + Semi-structured interviews)	168 stroke patient records for secondary data analysis, 24 stroke patients for prospective survey and 9 stroke patients for the qualitative study
16	Cunningham & Rhoda 2014	Eastern Cape	Urban	Community	Primary Literature (peer reviewed publications)	To determine the outcome of stroke patients in Eastern cape	Concurrent Mixed Methods design	24 Stroke Patients (Quantitative Survey)<br>9 Stroke patients (Qualitative interviews)
17	De la Cornillère (2007)	Western cape	Urban	BLRC Rehab centre	Dissertations	To describe the range of experiences of stroke patients relating to attendance or non-attendance of those referred to the Bishop Lavis Rehabilitation centre stroke group	Mixed Methods Descriptive study	20 participants with stroke for questionnaire survey and 6 stroke participants for FGD.

For peer review only

18	De Villiers et al 2009	Cape Town	Urban	Secondary hospital	Primary Literature (peer reviewed publications)	To examine the impact of multidisciplinary stroke care on the in-hospital mortality, resource utilization, and access to inpatient rehabilitation facilities for stroke patients admitted in Stroke units at a secondary hospital in Cape Town, South Africa	Cross-sectional pre and post study design	195 stroke patients
19	De Villiers 2011	Cape Town	Urban	District hospital	Primary Literature (peer reviewed publications)	To determine survival, disability and functional outcomes of stroke patients following their discharge from an acute stroke unit in an urban community with limited rehabilitative resources	Cross-sectional pre and post study design	196 stroke patients
20	Elloker (2015)	Western Cape	Urban	CHC	Dissertations	To determine participation restrictions and social support in patients with stroke, living in the Western Cape.	Mixed methods (Systematic Review + Quantitative survey)	106 stroke patients
21	Faux (2006)	Non-specific	Undefined	Undefined	Primary Literature (peer reviewed publications)	To provide a practical guide to helping stroke survivors who have a persistent disability maintain and enhance the gains made in rehabilitation	Narrative review recommendation	NA
22	Felemengas (2005)	Johannesburg Gauteng	Urban	Academic hospital	Dissertations	To investigate the family dynamics within the family system, as well as how these have evolved or changed following a stroke.	Qualitative	6 primary caregivers of stroke survivors
23	Groenewald & Rhoda 2017	Western Cape	Urban	Non-Governmental facility	Primary Literature (peer reviewed publications)	To determine outcomes of stroke patients managed by a multidisciplinary team at a step-down facility in the Western Cape.	A longitudinal observational study	68 stroke patients
24	Groenewald (2018)	Western Cape	Urban	Step down rehabilitation facility	Dissertations	To adapt and contextualize the original UK Bridges stroke SMI workbook for implementation with the South African stroke	A qualitative exploratory study -Interview, FGD, Expert	13 Health care professionals<br>12 Stroke patients<br>Expert Panel

For peer review only

						population	consultation	
25	Hassan et al (2011)	Western cape	Urban	Western Cape Rehabilitation centre	Primary Literature (peer reviewed publications)	To explore levels of strain experienced by caregivers and the variables that impact on their strain.	Concurrent, mixed method, descriptive design	57 caregivers of stroke survivors
26	Hilton (2011)	Johannesburg	Urban	Community	Dissertations	To establish the functional level of patients, the level of strain and quality of life of the caregiver six to 36 months post-stroke, and the influence of demographic factors, caregiver strain and patient's functional ability on quality of life of the caregiver.	Cross-sectional study	35 stroke patients and their caregivers
27	Hossain (2016)	Kwa-Zulu-Natal	Urban	Ladysmith Regional Hospital	Dissertations	To investigate the Need for Palliative care in Cerebrovascular Accident (stroke) patients at Ladysmith Regional Hospital	Mixed Methods (qualitative and quantitative)	72 stroke patients for quantitative study and<br>10 stroke patients for qualitative study
28	Joseph 2012	Western cape	Urban	WCRC – Rehabilitation centre	Dissertations	To determine the process of rehabilitation and the outcome of patients following in-patient rehabilitation at a facility in the Western Cape	A descriptive, observational, longitudinal design	76 Spinal Cord Injury patients and 67 stroke patients. Total patients (including drop outs) 130.
29	Kleineibst (2007)	Western Cape	Urban	Community	Dissertations	To determine the effectiveness of a caregiver support intervention programme to address the need for primary caregivers of stroke survivors in Bishop Lavis Community	Prospective descriptive qualitative study	29 caregivers of stroke survivors
30	Kotsokoane et al (2018)	Gauteng	Urban	CHC	Primary Literature (peer reviewed publications)	To determine the level of integration of stroke survivors at Soshanguve community clinics.	Retrospective quantitative research design	114 stroke survivors
31	Kusambiza-Kiingi (2016)	Johannesburg Gauteng	Urban	CHC	Primary Literature (peer reviewed publications)	To determine stroke survivors' levels of community reintegration, quality of life (QOL),<br>satisfaction with the physiotherapy services and the	Cross-sectional study	108 stroke survivors and 45 caregivers

For peer review only

						level of caregiver strain at community health centres within the Johannesburg		
32	Leichtfuss (2009)	Western Cape	Urban	Private acute care hospitals	Dissertations	To examine the practice of doctors with regards to stroke rehabilitation in private acute-care hospitals private acute-care hospitals and to evaluate information shared between doctors and pts i.r.t prognosis, severity, discharge, referral, timing of discharge planning and decision making. in the Western Cape Metropole	Retrospective and descriptive design	37 doctors treating and discharging stroke patients<br>48 pts
33	Mabunda (2015)	Western Cape	Urban	Private not for profit Intermediate care Facility	Dissertations	To describe the model of service provision at an IC facility and the role it plays in the continuity of care in Cape Town.	Cross sectional survey	68 stroke patients<br>70 clinical staff
34	Makganye (2015)	Gauteng?	Urban	CHC	Dissertations	To investigate the physical, psychological, social, religious	Qualitative design	5 stroke patients and 5 caregivers
35	Maleka et al (2012)	Limpopo and Gauteng	Urban and rural	Community	Primary Literature (peer reviewed publications)	To establish the experience of people living with stroke in low socioeconomic urban and rural areas of South Africa	Qualitative study design	32 stroke survivors living in the community
36	Mamabolo et al (2008)	Gauteng	Urban	PHC clinics	Primary Literature (peer reviewed publications)	to establish what demographic, environmental and physical factors influence functional independence post stroke.	Cross-sectional study	68 stroke patients
37	Mandizvidza (2017)	Western Cape	Urban	Level 1 2 3 hospitals	Dissertations	To describe the acute and post-acute ischaemic stroke services offered to ischaemic stroke patients in level 1, 2, and 3 hospitals in the Cape Metro Health District, compare these services to the national guideline and identify any barriers to optimum stroke	Descriptive cross-sectional study	10 doctors and 10 nurses from stroke ward and 8 doctors from emergency ward; pt records

For peer review only

						patient care.		
38	Mashau et al 2016	Limpopo	Rural and Urban	HBC organization	Primary Literature (peer reviewed publications)	To investigate the impact of caregiving on voluntary home-based caregivers.	A quantitative cross-sectional descriptive survey	190 home-based caregivers
39	Masuku et al (2018)	Gauteng	Urban	Community	Primary Literature (peer reviewed publications)	To describe the caregiving experience of female caregivers of PWA residing in Tembisa, a township situated in the east of Johannesburg	Qualitative study	14 primary caregivers of stroke survivors with Aphasia
40	Matshikiza (2019)	Western cape	Urban	Tertiary Hospital	Dissertations	to determine the pre-hospital barriers and in-hospital delays to emergency care for patients presenting to Groote Schuur Hospital (GSH) with acute stroke.	prospective, observational study	50 patients with stroke
41	Mudzi (2010)	Gauteng	Urban	Community	Primary Literature (peer reviewed publications)	To establish the impact of caregiver education on the morbidity of the stroke survivors and on the quality of life of the stroke survivors and their carers.	A stratified randomised controlled trial	200 stroke patients and caregivers
42	Mudzi et al (2013)	Gauteng	Urban	Community	Dissertations	to establish the extent of community participation and the barriers and facilitators to the participation for stroke patients after their discharge.	longitudinal study	200 patients with first-time ischaemic stroke
43	Ntamo (2011)	Eastern Cape	Urban	Mthatha General Hospital	Dissertations	To identify factors that influence poor attendance for outpatient physiotherapy by patients discharged from MGH with a stroke.	Mixed methods (Qualitative + Quantitative study)	85 stroke patients attending Physiotherapy at MGH
44	Parekh and Rhoda (2013)	Western Cape	Urban	Tertiary hospital	Primary Literature (peer reviewed publications)	To determine functional outcomes and factors influencing functional outcomes of stroke patients admitted to a South African tertiary hospital	Longitudinal Pre and Post test design	100 stroke patients
45	Parekh	Western	Urban	Tertiary	Dissertations	to identify factors influencing	A descriptive,	66 stroke patients

For peer review only

	(2011)	Cape		hospital		functional outcome of stroke patients admitted to a South African tertiary hospital	observational, longitudinal quantitative study design	
46	Posner (2015)	Gauteng	Urban	Community	Dissertations	to explore the experiences and perceived needs of employed caregivers working for patients who have suffered from a stroke within home settings in South Africa.	Qualitative research design with Interviews + FGDs	15 employed caregivers working at the homes of stroke survivors FGDs with 10 participants 5 in each group
47	Ras (2009)	Western Cape	Urban	NGO Run Hospital booth	Dissertations	to assess the quality of the stroke rehabilitation services at Booth Memorial Hospital.	Cross-sectional audit of records	NA
48	Rhoda (2009)	Western Cape	Peri - Urban	CHC	Primary Literature (peer reviewed publications)	to determine the structure and process of rehabilitation of stroke patients at Community Health Centres (CHCs) in the Western Cape	Quantitative cross-sectional survey	100 first time stroke patients and 16 therapists
49	Rhoda et al (2011)	Western Cape	Peri - Urban	CHC	Primary Literature (peer reviewed publications)	to determine the activity limitations of stroke patients receiving rehabilitation at out-patient Community health Centres (ChCs)	Longitudinal observational study	100 patients with stroke
50	Rhoda (2014)	Western Cape	Peri - Urban	CHC	Primary Literature (peer reviewed publications)	to determine the quality of life and factors influencing quality of life of community-dwelling stroke patients living in low-income, peri-urban areas in the Western Cape, South Africa.	Observational, longitudinal study	100 first time stroke patients
51	Rhoda et al (2015)	South Africa (Eastern Cape) Tanzania and Rwanda	Undefined	Provincial hospital	Primary Literature (peer reviewed publications)	the provision of inpatient rehabilitation and the post discharge challenges of stroke survivors in specific African countries.	Retrospective survey and interviews	168 SA stroke patients 145 Tanzanian and 130 Rwandan stroke patients 9 SA patients, 10 TP and 10 RP for qualitative study
52	Rouillard et al (2012)	Western Cape	Urban	WCRC rehab centre	Primary Literature (peer reviewed publications)	To determine activity limitations, participation restrictions, health-related quality of life and caregiver strain in community-dwelling	Longitudinal and descriptive study	46 stroke patients 41 caregivers

For peer review only

						stroke survivors discharged from an intensive inpatient rehabilitation programme at 6 months post stroke.		
53	SA-CSRG 2019	National	NA	NA	Primary literature (Peer-reviewed, Clinical Guideline)	contextualised development of stroke rehabilitation guideline	Guideline	NA
54	Scheffler and Mash 2019	Western Cape	Rural	Community	Primary Literature (peer reviewed publications)	To describe and analyze the outcomes of patients with stroke from a rural PHC setting in the Western Cape, South Africa.	Longitudinal survey	93 stroke patients
55	Smith (2019)	Western Cape	Western Cape	Urban and Rural	Community	To explore the self-management strategies employed by stroke survivors in the Western Cape, South Africa	Exploratory qualitative design (In-depth Interviews)	14 stroke survivors
56	Taylor & Ntusi (2019)	South Africa	South Africa	Undefined	Undefined	To improve management of stroke in South Africa	Editorial (Review)	NA
57	Thomas & Greenop (2008)	Gauteng	Gauteng	Urban	Community	To investigate into the complexities of caregiving, including both perceptions and experiences of the healthcare system.	Qualitative design (interviews)	6 caregivers of stroke survivors
58	Viljoen (2014)	Western Cape	Western Cape	Urban	Groote Schuur Hospital	To determine the cost of stroke care and to identify factors associated with increased expense, as well as to evaluate the quality of stroke care in general medical wards	Review of records	261 stroke patient (records)
59	Wasserman et al 2009	KwaZulu-Natal	KwaZulu-Natal	Rural	Community	To assess discharge planning of stroke patients and to evaluate integration and continuity of stroke care between hospital and community	Quantitative design (Survey)	30 stroke patients

Supplementary file S4: Components assessed in included studies

Author (year)	Governance /Regulation	Resources	Service Delivery	[illegible]	Re-organisation of care	Community engagement
Arowoiya (2014)			X	X		
Bham & Ross 2005			X	X		
Biggs (2005)		X	X	X		X
Biggs & Rhoda (2008)				X		
Blackwell & Littlejohns (2010)			X			
Botha (2008)					X	X
Bryer (2009)		X	X			
Bryer et al (2010)	X					
Burton 2016					X	X
Cawood 2012		X	X			X
Cawood& Visagie (2015)			X			X
Cawood & Visagie (2016)			X			X
Cawood et al (2016)						x
Connor (2005)	X	X		X		
Cunningham (2012)		X	X	X		X
Cunningham & Rhoda (2014)						X
De la Cornillère (2007)			X	X		X
De Villiers et al (2009)			X			
De Villiers (2011)		X	X			
Elloker (2015)						X
Faux (2006)					X	
Felemengas (2005)			X	X		X
Groenewald & Rhoda (2017)			X			
Groenewald (2018)			X		X	
Hassan et al (2011)				X		
Hilton (2011)			X	X		
Hossain (2016)			X		X	
Joseph (2012)			X			X
Kleineibst (2007)		X	X	X		
Kotsokoane et al (2018)			X			X
Kusambiza-Kiingi (2016)			X	X		
Leichtfuss (2009)		X	X			X
Mabunda (2015)			X			
Makganye (2015)		X	X	X		
Maleka et al (2012)		X		X		X
Mamabolo et al (2008)						X
Mandizvidza (2017)	X	X	X			
Mashau et al (2016)			X			
Masuku et al (2018)			X	X		
Matshikiza (2019)		X	X			
Mudzi (2010)			X	X		X
Mudzi et al (2013)			X	X		

For peer review only

Ntamo (2011)		X	X	X		X
Parekh and Rhoda (2013)			X			
Parekh (2011)			X			
Posner (2015)			X			
Ras (2009)		X	X			
Rhoda (2009)		X	X			
Rhoda et al (2011)			X			
Rhoda (2014)			X	X		
Rhoda et al (2015)			X	X		
Rouillard et al (2012)			X	X		
SA-CSRG	X					
Scheffler and Mash (2019)			X			
Smith (2019)			X	X		
Taylor & Ntusi (2019)			X			
Thomas & Greenop (2008)			X	X		
Viljoen (2016)		X	X			
Wasserman et al (2009)			X			
**Total**	**4**	**16**	**47**	**24**	**5**	**19**

\]

For peer review only

Preferred Reporting Items for Systematic reviews and Meta-Analyses extension for Scoping Reviews (PRISMA-ScR) Checklist

SECTION	ITEM	PRISMA-ScR CHECKLIST ITEM	REPORTED ON PAGE #
**TITLE**			
Title	1	Identify the report as a scoping review.	1
**ABSTRACT**			
Structured summary	2	Provide a structured summary that includes (as applicable): background, objectives, eligibility criteria, sources of evidence, charting methods, results, and conclusions that relate to the review questions and objectives.	2
**INTRODUCTION**			
Rationale	3	Describe the rationale for the review in the context of what is already known. Explain why the review questions/objectives lend themselves to a scoping review approach.	4
Objectives	4	Provide an explicit statement of the questions and objectives being addressed with reference to their key elements (e.g., population or participants, concepts, and context) or other relevant key elements used to conceptualize the review questions and/or objectives.	4
**METHODS**			
Protocol and registration	5	Indicate whether a review protocol exists; state if and where it can be accessed (e.g., a Web address); and if available, provide registration information, including the registration number.	6
Eligibility criteria	6	Specify characteristics of the sources of evidence used as eligibility criteria (e.g., years considered, language, and publication status), and provide a rationale.	6
Information sources*	7	Describe all information sources in the search (e.g., databases with dates of coverage and contact with authors to identify additional sources), as well as the date the most recent search was executed.	6
Search	8	Present the full electronic search strategy for at least 1 database, including any limits used, such that it could be repeated.	6
Selection of sources of evidence†	9	State the process for selecting sources of evidence (i.e., screening and eligibility) included in the scoping review.	6
Data charting process‡	10	Describe the methods of charting data from the included sources of evidence (e.g., calibrated forms or forms that have been tested by the team before their use, and whether data charting was done independently or in duplicate) and any processes for obtaining and confirming data from investigators.	6
Data items	11	List and define all variables for which data were sought and any assumptions and simplifications made.	6-7
Critical appraisal of individual sources of evidence§	12	If done, provide a rationale for conducting a critical appraisal of included sources of evidence; describe the methods used and how this information was used in any data synthesis (if appropriate).	7
Synthesis of results	13	Describe the methods of handling and summarizing the data that were charted.	7

For peer review only - http://bmjopen.bmj.com/site/about/guidelines.xhtml

SECTION	ITEM	PRISMA-ScR CHECKLIST ITEM	REPORTED ON PAGE #
**RESULTS**			
Selection of sources of evidence	14	Give numbers of sources of evidence screened, assessed for eligibility, and included in the review, with reasons for exclusions at each stage, ideally using a flow diagram.	7-17
Characteristics of sources of evidence	15	For each source of evidence, present characteristics for which data were charted and provide the citations.	7-17
Critical appraisal within sources of evidence	16	If done, present data on critical appraisal of included sources of evidence (see item 12).	7-17
Results of individual sources of evidence	17	For each included source of evidence, present the relevant data that were charted that relate to the review questions and objectives.	7-17
Synthesis of results	18	Summarize and/or present the charting results as they relate to the review questions and objectives.	7-17
**DISCUSSION**			
Summary of evidence	19	Summarize the main results (including an overview of concepts, themes, and types of evidence available), link to the review questions and objectives, and consider the relevance to key groups.	17
Limitations	20	Discuss the limitations of the scoping review process.	21
Conclusions	21	Provide a general interpretation of the results with respect to the review questions and objectives, as well as potential implications and/or next steps.	21
**FUNDING**			
Funding	22	Describe sources of funding for the included sources of evidence, as well as sources of funding for the scoping review. Describe the role of the funders of the scoping review.	22

JBI = Joanna Briggs Institute; PRISMA-ScR = Preferred Reporting Items for Systematic reviews and Meta-Analyses extension for Scoping Reviews.

* Where *sources of evidence* (see second footnote) are compiled from, such as bibliographic databases, social media platforms, and Web sites.

† A more inclusive/heterogeneous term used to account for the different types of evidence or data sources (e.g., quantitative and/or qualitative research, expert opinion, and policy documents) that may be eligible in a scoping review as opposed to only studies. This is not to be confused with *information sources* (see first footnote).

‡ The frameworks by Arksey and O'Malley (6) and Levac and colleagues (7) and the JBI guidance (4, 5) refer to the process of data extraction in a scoping review as data charting.

§ The process of systematically examining research evidence to assess its validity, results, and relevance before using it to inform a decision. This term is used for items 12 and 19 instead of "risk of bias" (which is more applicable to systematic reviews of interventions) to include and acknowledge the various sources of evidence that may be used in a scoping review (e.g., quantitative and/or qualitative research, expert opinion, and policy document).

From: Tricco AC, Lillie E, Zarin W, O'Brien KK, Colquhoun H, Levac D, et al. PRISMA Extension for Scoping Reviews (PRISMAScR): Checklist and Explanation. Ann Intern Med. 2018;169:467–473. doi: 10.7326/M18-0850.

For peer review only - http://bmjopen.bmj.com/site/about/guidelines.xhtml
